# Scalable Synthesis of a Masked Thiophenol as an Air-Stable, Odorless Intermediate to the Aurora Kinase Inhibitor Tozasertib (VX-680)

**DOI:** 10.1021/acs.oprd.6c00143

**Published:** 2026-08-06

**Authors:** Richard Herzog, Olivia L. Munns, Valentin Magne, Stephen P. Argent, Liam T. Ball

**Affiliations:** † Institute of Organic Chemistry, 9174 Albert-Ludwigs-Universität Freiburg, Albertstraße 21, 79104 Freiburg im Breisgau, Germany; ‡ School of Chemistry, 1980 University of Bristol, Bristol BS8 1TS, U.K.; § Université de Toulouse, CNRS, CEMES, Toulouse 31062, France; ∥ School of Chemistry, 6123 University of Nottingham, Nottingham NG7 2RD, U.K.

**Keywords:** thiophenols, C–S cross-coupling, Ni catalysis, nucleophilic aromatic substitution, design of experiments, thermal hazard assessment

## Abstract

The use of thiophenols on process scales is fraught with challenges relating to their toxicity, malodor, and air sensitivity. We previously demonstrated that the Ni-catalyzed C–S coupling of aryl iodides and thiourea provides a discovery-scale route to *S*-aryl isothiouronium salts, which serve as bench stable, odorless, and highly crystalline surrogates of thiophenols. Here, we report the process optimization of this methodology for the synthesis of a key intermediate to kinase inhibitor tozasertib (VX-680). The corollary is a reduction in catalyst loading to 0.5 mol %, elimination of NMP solvent, and introduction of a direct crystallization, giving 95% yield on a 100 g scale.

## Graphical abstract

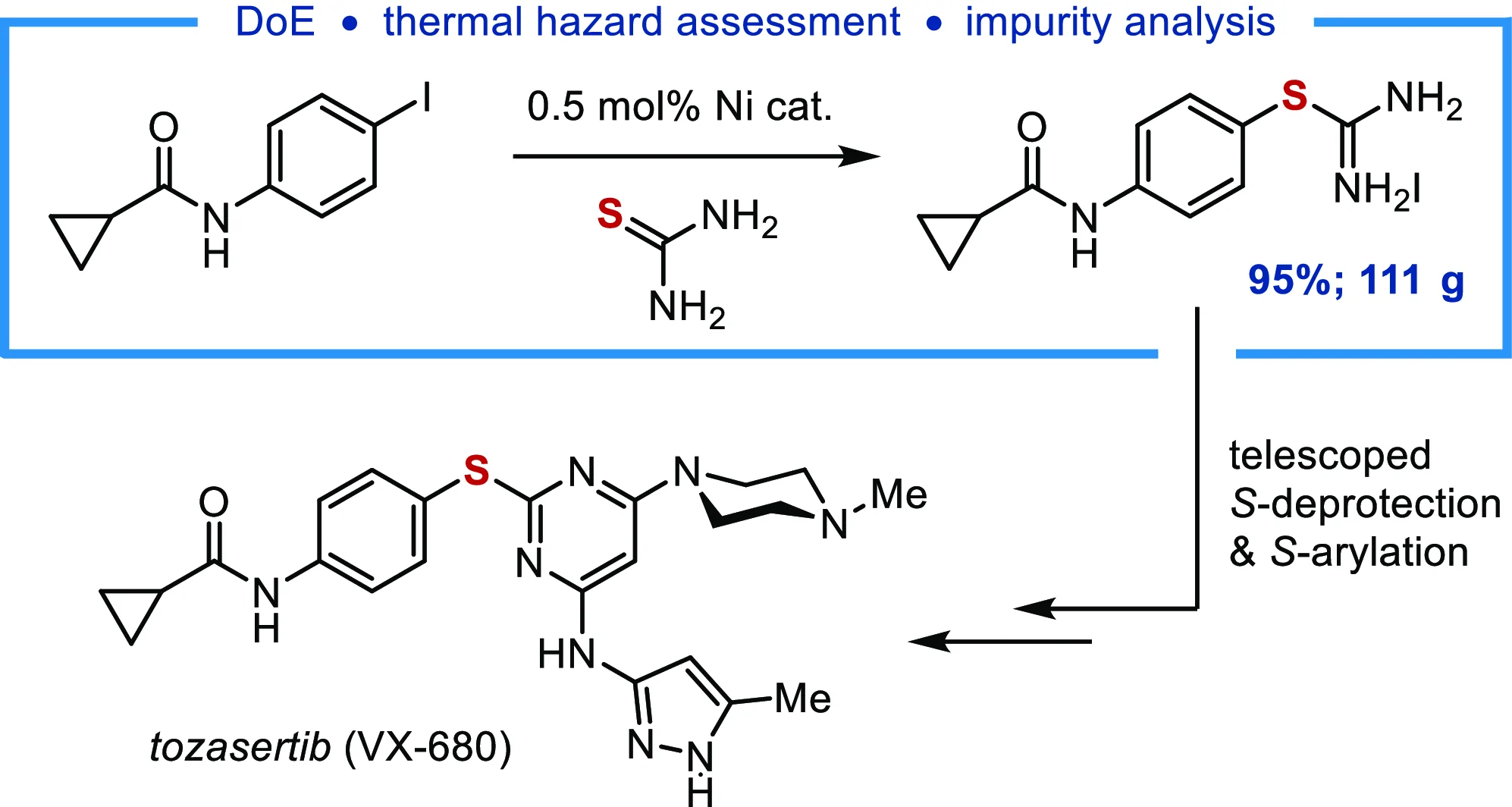

## Introduction

The aryl-sulfur substructure is found in numerous drugs, agrochemicals, flavorings, and fragrances.^1–5^ Despite the diversity of substitution patterns and oxidation states that sulfur adopts in these compounds, all such motifs are ultimately accessible from a single class of precursors: the parent thiophenol. The synthetic versatility of thiophenols is, however, underexploited at process scales due to well-documented^6–9^ issues that include air sensitivity, high toxicity,^10,11^ and extreme malodor.^12^ While these challenges can often be accommodated in discovery laboratories, they become increasingly difficult to address within the operating constraints and regulatory frameworks of the plant, and are further exacerbated by the poor availability of thiophenols in reliably high purities.

The need to procure and manipulate thiophenols can be avoided by instead preparing a masked equivalent via Migita coupling,^13,14^ then revealing and functionalizing the sulfur atom in a single, telescoped operation.^8,15^ To this end, we previously reported a general method for the synthesis of air-stable, odorless thiophenol equivalents via Ni-catalyzed cross-coupling of aryl iodides and thiourea (Scheme 1A).^16^ The C–S coupling—which builds on the seminal work of Takagi^17^—uses a cheap, air-stable, odorless S-source (thiourea) and occurs under base-free conditions that confer compatibility with a broad range of functional groups (Scheme 1B). The resulting isothiouronium salts are isolated conveniently by filtration following anion metathesis with sodium 3,5-dinitrobenzoate (DNB), and can be deprotected with mild base prior to *in situ* S-functionalization under polar^16^ or photoredox^18^ conditions. In addition to these appealing attributes, the potential suitability of our methodology for deployment on process scales is complemented by the use of a base-metal catalyst.^19–21^

**Scheme 1:**
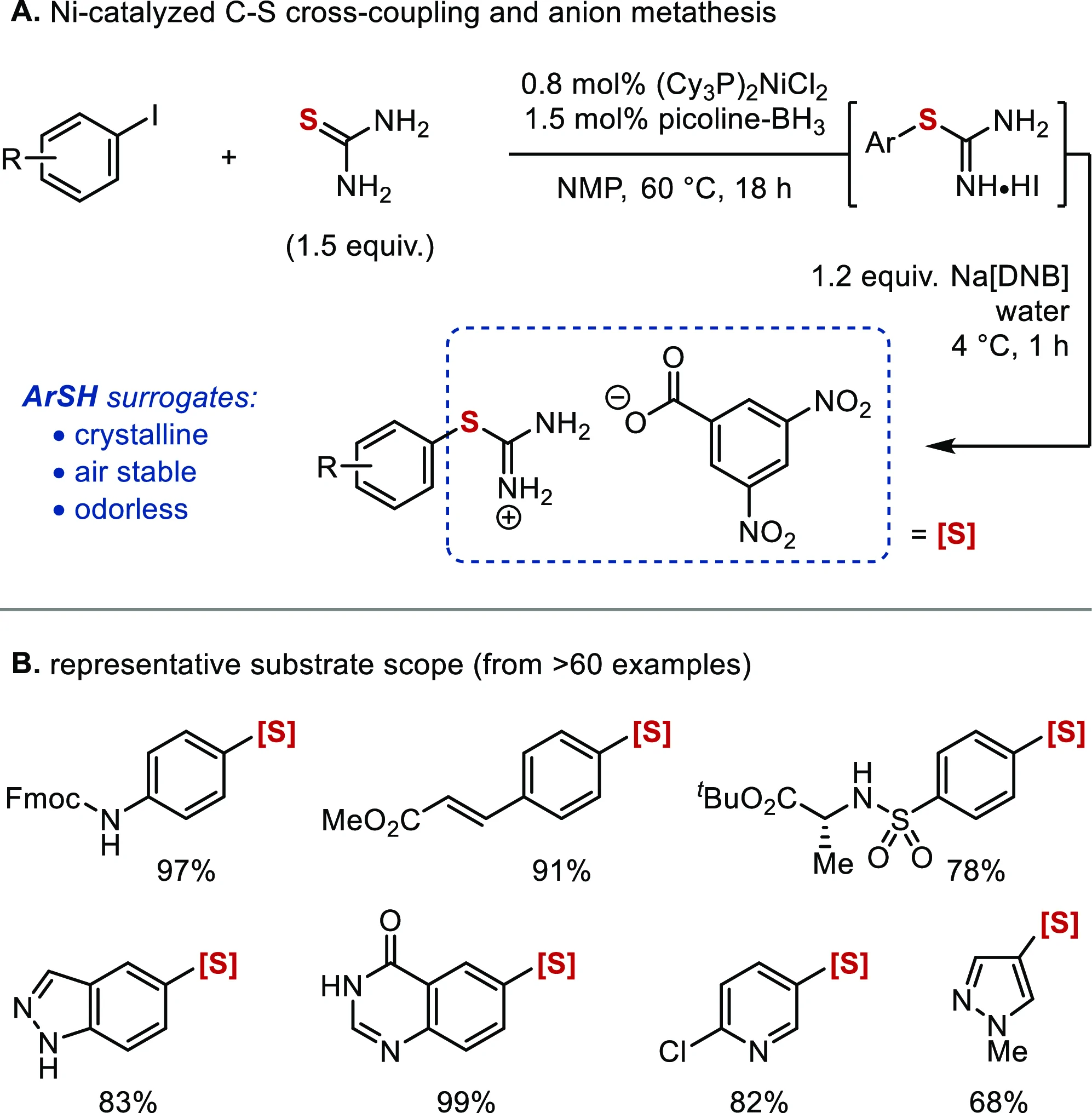
Synthesis of Thiophenol Surrogates^16^

Although we had previously demonstrated this methodology on up to a 100 mmol scale, our original optimization campaign had focused on identifying conditions that were applicable to a wide range of aryl iodides.^16^ This drive for generality came at the expense of several factors relevant to reaction scale up, including the use of:

(1) NMP as solvent. In addition to being on the substances of very high concern candidate list—and therefore highly undesirable^19,22–24^ —we found that residual NMP was occasionally difficult to remove from the isolated isothiouronium salts, such that repeat purification was required.
(2) Tricyclohexylphosphine as ligand. The low cost of the nickel metal catalyst is somewhat overshadowed by the relatively higher price of the phosphine ligand, or indeed of the preformed (Cy_3_P)_2_NiCl_2_ complex. To both improve economy, and to reduce the amount of toxic metal used, we were therefore keen to explore lower-cost ligands and lower overall loadings of catalyst.
(3) Anion metathesis with sodium 3,5-dinitrobenzoate (Na­[DNB]) to facilitate product precipitation. While this process allowed chromatography-free product isolation without case-by-case optimization, a number of issues are introduced: (a) the DNB salts are typically fine solids that suspend in the aqueous NMP mixture, which can make filtration challenging on multigram scales; (b) the iodide-for-DNB exchange reduces mass efficiency, as the counteranion is discarded in subsequent manipulations; (c) the generation of an aqueous waste stream that is contaminated with Ni, thiourea, and NMP; (d) the introduction of a nitroarene, a known toxicophore^25,26^ and a potential explosiphore.^27,28^

(4) Excess thiourea. While a convenient and low-cost S-atom source, thiourea is toxic^29^ and its use in excess is both atom inefficient and a potential source of contamination for the product.

Thus, while our method has good potential for scalability, it currently does not meet the SELECT requirements of scale up.^30^ In this communication we address the above challenges for the specific example of thioether **1** (Scheme 2A), a key intermediate in the synthesis of aurora kinase inhibitor tozasertib (VX-680).^31,32^ While clinical trials were halted due to safety concerns,^33^ tozasertib presents an interesting biological profile that includes inhibition of several cancer-related kinases,^31^ such as RIPK1 ^34,35^ and a multidrug resistant mutant of Abl.^36^ It has also served as a starting point for the development of inhibitors of the pain target TrkA.^37^

**Scheme 2:**
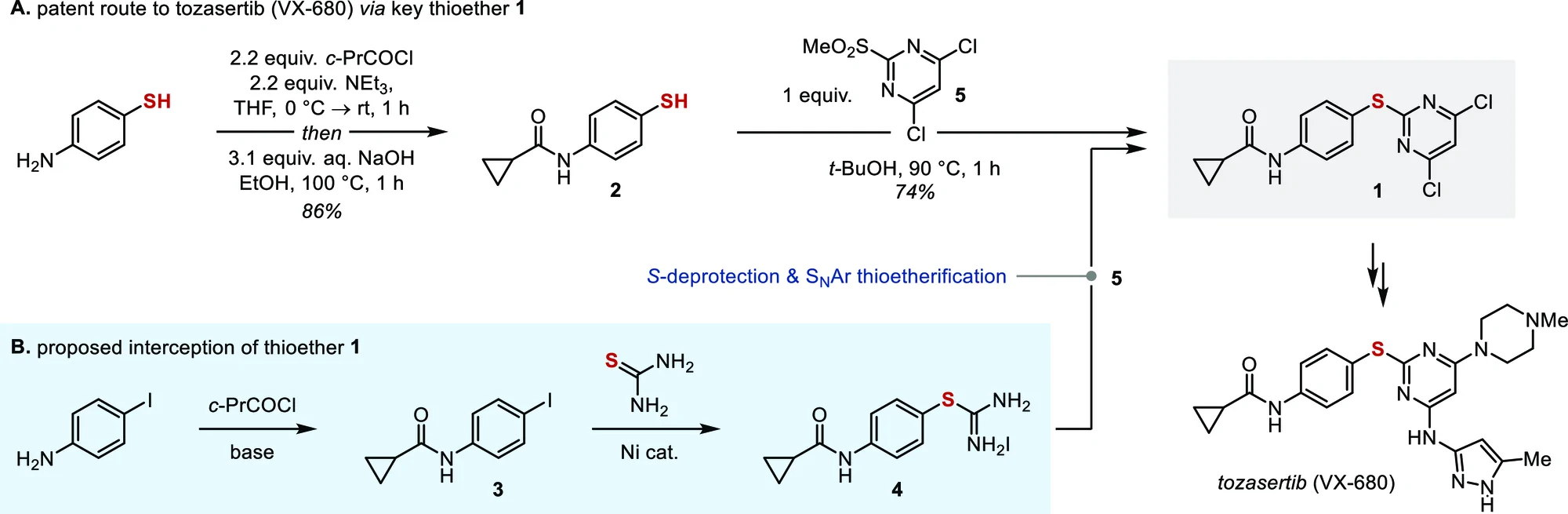
Patent Route to Tozasertib (A)^38^ and Proposed Interception as a Showcase for Ni-Catalyzed C–S Cross-Coupling (B)

The patent route^38^ to thioether **1** requires the synthesis and isolation of free thiophenol **2** (Scheme 2A), and the ultimate starting material—4-aminothiophenol—is both acutely toxic (H302) and corrosive to skin (H314).^39^ We envisaged an alternative approach in which the preparation of iodoanilide **3** precedes installation of sulfur via our C–S cross-coupling methodology (Scheme 2B). The resulting isothiouronium salt **4** would serve as a stable, crystalline, and odorless intermediate from which thiophenol **2** could be liberated *in situ* prior to the final S_N_Ar thioetherification with pyrimidine sulfone **5**.

In this communication we describe the translation of our discovery-oriented methodology to a scalable process that meets SELECT criteria. With insight from Design of Experiments and thermal hazard analysis, we demonstrate that the key C–S coupling can be performed reliably on a 100 g scale (95% yield; >99% qNMR purity), and that the resulting isothiouronium salt **4** can be used as a direct replacement for thiophenol **2** in the synthesis of tozasertib intermediate **1**. By choosing a pharmaceutically relevant target for this study, we hope to provide the process community with the confidence to employ our methodology in their own programs.

## Results and Discussion

We initiated our study by preparing the requisite iodoanilide **3** from 4-iodoaniline and cyclopropanecarbonyl chloride (Scheme 3). Straightforward product isolation was enabled by judicious choice of acetone as solvent and K_2_CO_3_ as base: upon reaction completion, addition of water served to dissolve the inorganic coproducts, hydrolyze residual acid chloride, and form a single liquid phase from which iodoanilide **3** crystallized in excellent yield.

**Scheme 3:**
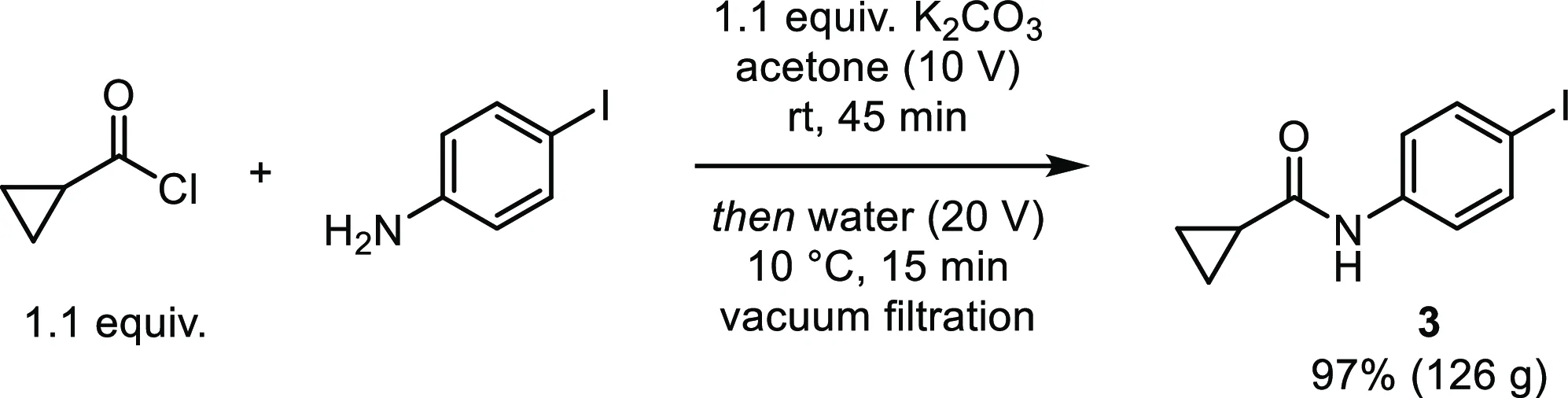
Synthesis of Iodoanilide **3**

### Optimization of C–S Cross-Coupling

The cross-coupling of iodoanilide **3** and thiourea proceeded smoothly under our previously reported conditions (Scheme 4),^16^ thereby confirming the compatibility of this substrate with the key C–S bond-forming step. Anion metathesis with aqueous sodium 3,5-dinitrobenzoate allowed for isolation of isothiouronium dinitrobenzoate **6**, albeit in modest yield. We ascribe this to the relatively small size and needle-like shape of the product particles (*ca.* 2 × 10 μm^2^; Scheme 4, inset), which rendered filtration challenging and ultimately required repeat washes to effect the complete removal of both water and NMP. Combined with safety concerns raised during subsequent thermal hazard assessment (*vide infa*), this practical challenge validated our decision to remove 3,5-dinitrobenzoate from the workflow.

**Scheme 4:**
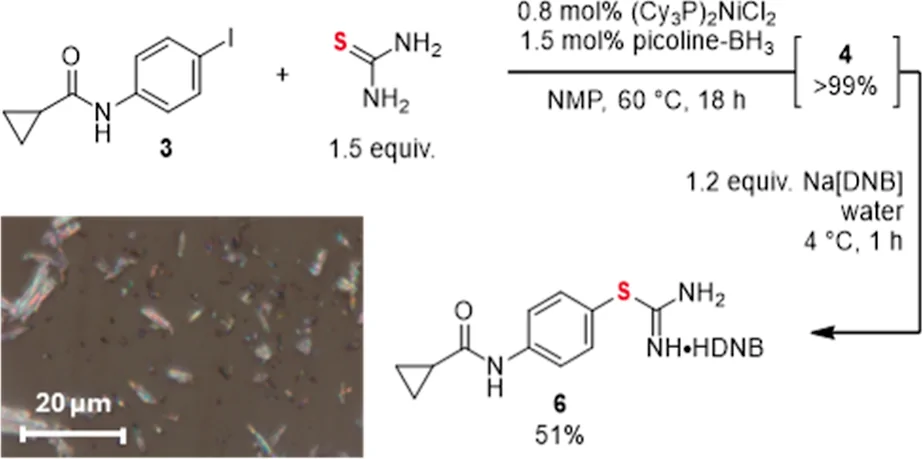
Synthesis of Isothiouronium Dinitrobenzoate **6** ^a^

a Inset image obtained by reflectance microscopy.

With the aim of facilitating purification and improving atom economy, subsequent exploratory reaction screening was performed with a reduced stoichiometry of thiourea (1.05 vs 1.5 equiv). Under otherwise unaltered conditions, the coupling proceeded smoothly with both (Cy_3_P)_2_NiCl_2_ and (Ph_3_P)_2_NiCl_2_ as precatalysts (Table 1, Entries 1 and 2). Notably, the latter is some 20 times cheaper than the former on a mole-for-mole basis (*e.g.*, Merck-Millipore, March 2026).

**Table 1:**
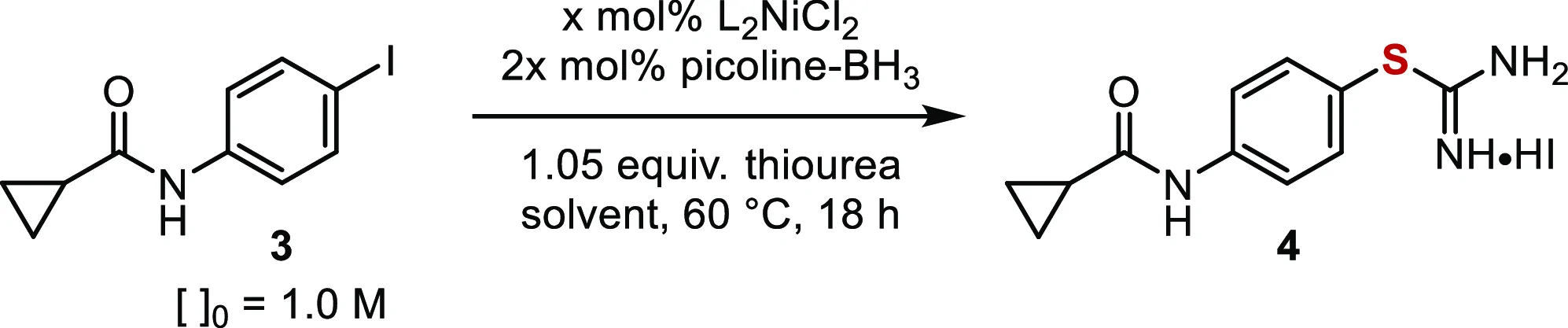
Preliminary Investigation of C–S Cross-Coupling^a^

entry	*x*	L	solvent	% yield
1	1	PCy_3_	NMP	96
2	1	PPh_3_	NMP	94
3	1	PPh_3_	PC	72
4	1	PPh_3_	*i*-PrOH	87
5	1	PPh_3_	GVL	91
6	1	PPh_3_	2-pyrrolidinone	89
7	0.7	PPh_3_	*i*-PrOH	82
8	0.7	PPh_3_	GVL	98
9	0.7	PPh_3_	2-pyrrolidinone	45
10	0.7	PPh_3_	*i*-PrOH	91^b^
11	0.7	PPh_3_	GVL	>99^b^
12	0.7	PPh_3_	2-pyrrolidinone	51^b^
13	0.5	PPh_3_	*i*-PrOH	70
14	0.5	PPh_3_	GVL	90
15	0.5	PPh_3_	2-pyrrolidinone	38

a Reactions were performed on 0.50 mmol scale in sealed crimp-cap microwave vials. Yields determined by ^1^H NMR spectroscopic analysis vs internal standard (1,3,5-trimethoxybenzene).

b Using 1.2 equiv thiourea. PC, propylene carbonate; GVL, γ-valerolactone.

Seeking to replace NMP—and informed by earlier observations that the C–S coupling performs best in polar media and at high reaction concentrations (*ca.* 1 M)^16,18^ —we investigated solvents deemed acceptable by the selection guides of multiple companies (Table 1, Entries 3–6).^24,40–42^ Propylene carbonate (PC) proved a poor solvent: iodoanilide **3** and thiourea dissolved only slowly at 60 °C, and rapid precipitation of isothiouronium iodide **4** afforded a thick slurry that halted magnetic stirring and ultimately limited the yield to 72% (Entry 3). In contrast, homogeneous solutions and significantly higher yields were obtained in *i*-PrOH, γ-valerolactone (GVL), and 2-pyrrolidinone (Entries 4–6). The reaction performed equally well in both *i*-PrOH and GVL at a reduced catalyst loading of 0.7 mol % (Entries 7 and 8), whereas the yield was suppressed in 2-pyrrolidinone (Entry 9). Although increasing the stoichiometry of thiourea led to a noticeable improvement in yield at low catalyst loadings (Entries 10–12), samples of isothiouronium iodide **4** isolated from these reactions were visibly gray. A further reduction in the catalyst loading led to a *ca.* 10% decrease in reaction yield (compare Entries 13–15 to Entries 7–9).

To develop a more comprehensive understanding of the C–S coupling, and hence identify more robust conditions, we built upon these preliminary studies using Design of Experiments.^43^ Thus, with yield as the response, the following factors were considered: (a) catalyst loading, (0.25–0.75 mol %); (b) stoichiometry of picoline-borane (1–2 equiv relative to Ni); (c) stoichiometry of thiourea (1.0–1.5 equiv); and (d) temperature (50–70 °C). Initial studies using a two-level full factorial model suggested that all factors were significant, and that a square term was needed for good *Q* ^2^ (85%, vs 77% if the square term is omitted; *Q* ^2^ is the percentage of the variation of the response predicted by the model according to cross validation). The identity of the squared factor could not, however, be determined unambiguously using a simple two-level design. In contrast, use of a three-level full factorial design (Central Composite Face-Centered, CCF) identified catalyst loading as the squared factor and gave a slightly improved *Q* ^2^ of 88%.

While the C–S coupling performs best when all factors are high (Figure 1), the response surface predicts that the detrimental effect of reducing the catalyst loading can be effectively compensated by increasing the reaction temperature. The following conditions—which are predicted to give >98% yield—were thus selected for further investigation: 0.5 mol % (Ph_3_P)_2_NiCl_2_, 1 mol % picoline-borane, 1.0 equiv thiourea, 70 °C.

**Figure 1:**
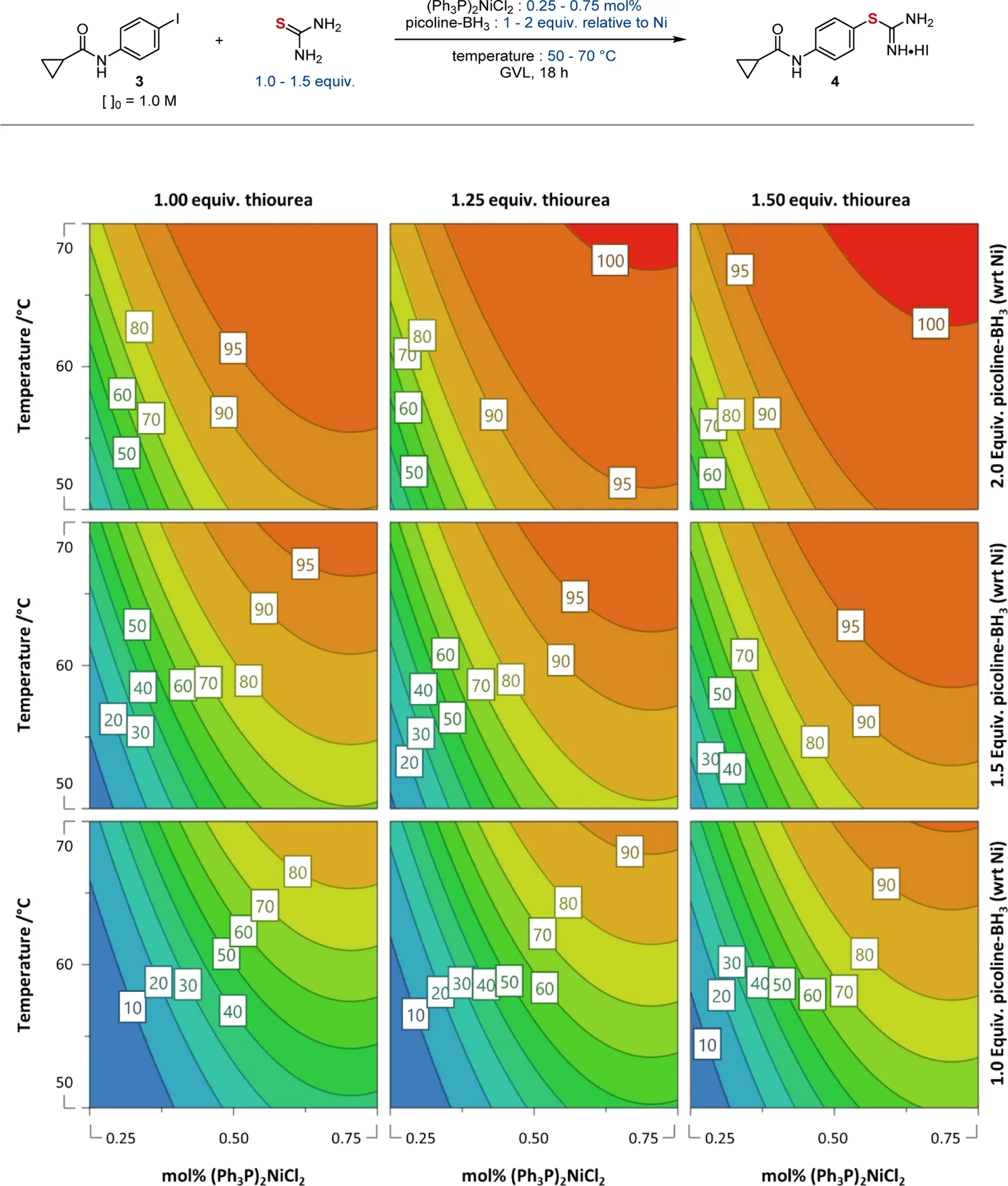
Response surfaces for C–S cross-coupling Design of Experiments illustrating the effect of thiourea stoichiometry (columns), picoline-borane stoichiometry (rows), catalyst loading (*x*-axes), and temperature (*y*-axes). Numerical values in white boxes refer to the % conversion of iodoanilide **3** to isothiouronium iodide **4**, as determined by ^1^H NMR spectroscopic analysis. Reactions were performed on 1.00 mmol scale in sealed crimp-cap microwave vials; see the Supporting Information for details of the experimental procedure, raw data, and analysis of the design.

### Scale-Up of C–S Cross-Coupling

Efficient precipitation of isothiouronium iodide **4** from the cross-coupling reaction mixture was achieved simply by adding two volume equivalents of isopropyl acetate (IPAc), then cooling to 4 °C for 16 h (Scheme 5A). Material isolated by filtration and washing with MTBE was obtained as a mixed hemisolvate containing 0.4 equiv of GVL and 0.1 equiv of IPAc; this composition was reproduced in five independent experiments and was not altered by further washing, trituration or drying *in vacuo*. The isolated solvate will henceforth be referred to as **4.solv** in order to differentiate it from the specific isothiouronium chemical entity **4**. The presence of solvent within the lattice of the isolated isothiouronium iodide was confirmed in an XRD structure of crystals grown from GVL/IPAc (Scheme 5B). The unit cell (Scheme 5B, left) contains two molecules of the salt, oriented head-to-tail such that the separation between the sulfur of one molecule and the carbonyl oxygen of the other is shorter than the sum of covalent radii (3.227 vs 3.320 Å). This short separation, and the relative geometry of the carbonyl and isothiouronium moieties, is consistent with chalcogen bonding.^44^ Extensive hydrogen bonding involving all four of the isothiouronium protons and the amide proton completes an extensive network of intermolecular interactions (Scheme 5B, right). The individual crystals of isothiouronium iodide **4.solv** (Scheme 5C, left) are markedly more block-like than the needles formed by dinitrobenzoate salt **6** (Scheme 4), and form spherical agglomerates of approximate diameter 80–120 μm (Scheme 5C, right). The physical form of the precipitate, combined with the use of a purely organic reaction mixture, presumably contributes to the ease of filtration observed for **4.solv**. Indeed, in contrast to dinitrobenzoate **6**, iodide **4.solv** exhibits higher filter rates, requires less washing, and the filter cake does not crack during drying.

**Scheme 5:**
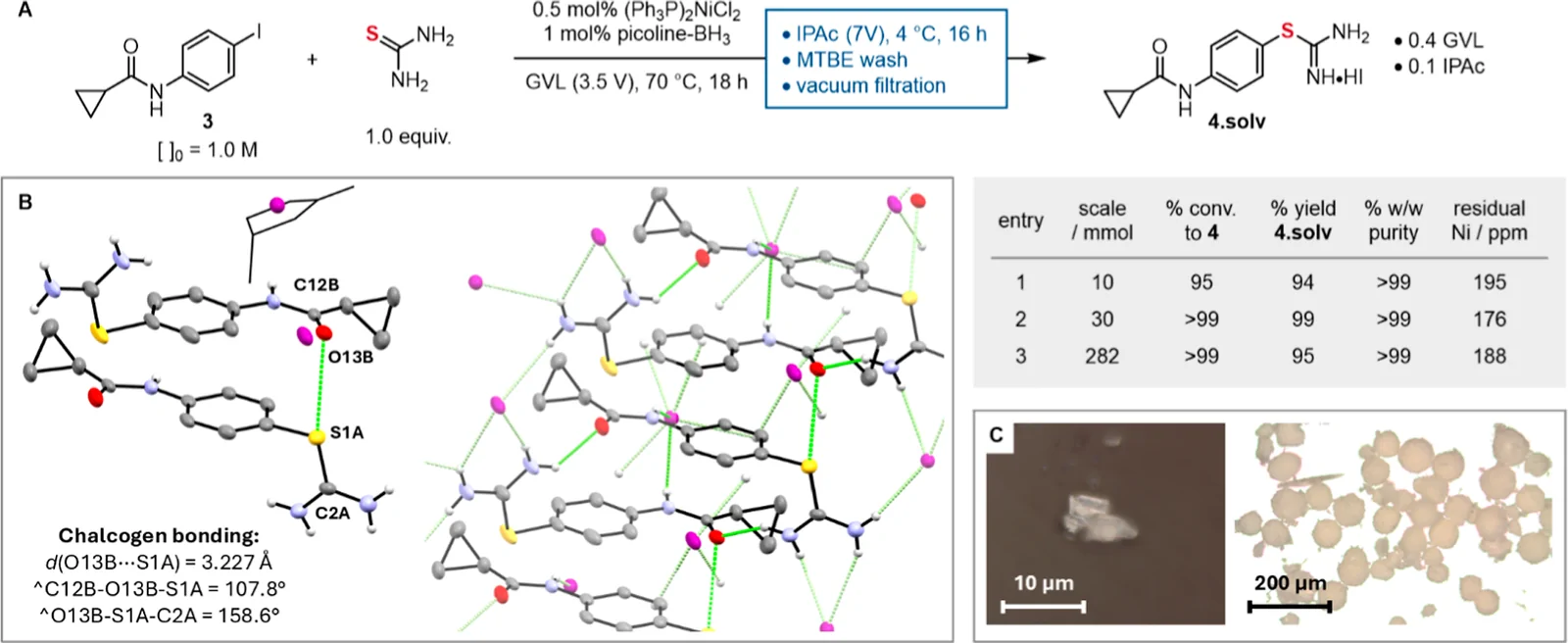
Synthesis and Isolation (A), Solid-State Structure (B), and Particle Form (C) of Isothiouronium Iodide **4.solv** ^a^

a Panel A: Conversions were determined by ^1^H NMR spectroscopic analysis of crude mixtures following dilution with DMSO-*d*_6_; yields refer to isolated material. Purity of isolated material was determined by ^1^H NMR spectroscopic analysis vs internal standard (1,3,5-trimethoxybenzene); residual Ni content determined by ICP-OES analysis (221.648 nm). Panel B: H atoms bonded to C omitted for clarity, and solvent molecules omitted from packing diagram; thermal ellipsoids shown at 50% probability; noncovalent contacts are shown in green. Panel C: Images obtained by reflectance microscopy.

Combined with the optimized cross-coupling conditions, this convenient isolation protocol allowed straightforward synthesis of isothiouronium iodide **4.solv** in reproducibly high yield and purity on increasingly large scales (Scheme 5A). This culminated with demonstration of the process on a 100 g scale (Entry 3). Residual Ni levels were determined to be below 200 ppm (ICP-OES) across all scales; while this is an order of magnitude higher than the 20 μg g^–1^ (oral intake) limit allowed under ICH guidelines,^45^ the Ni content was reduced significantly in subsequent operations.

### Thermal Hazard Assessment

At the outset of this study, we hypothesized that the nitroaryl explosiphore in isothiouronium dinitrobenzoate **6** could pose a potential safety risk, whereas isothiouronium iodide **4** would be thermally stable. The thermal properties of key compounds were therefore measured using Differential Scanning Calorimetry (DSC),^46^ and their impact sensitivity (IS) and explosive propagation (EP) potentials were calculated using the correlations developed by Yoshida^47^ and at Pfizer.^48^ Consistent with our hypothesis, isothiouronium iodide **4** exhibits a low enthalpy of decomposition, and is not identified as a thermal hazard (Table 2, Entry 1). In contrast, while isothiouronium dinitrobenzoate **6** also raises no concerns in the Yoshida correlation, it is identified as potentially shock sensitive by the more conservative Pfizer analysis (Entry 2). In addition to isothiouronium salts **4** and **6**, we also measured the thermal properties of 3,5-dinitrobenzoic acid (Entry 3): this compound is required in our original methodology^16^ for the synthesis of sodium dinitrobenzoate, and is a plausible coproduct from decomposition of isothiouronium dinitrobenzoates of type **6**. Alarmingly, 3,5-dinitrobenzoic acid fails the Yoshida test for impact sensitivity and is identified as a potential self-propagating explosive by both the Yoshida and Pfizer tests. Together, these measurements validated our initial objective of excluding the dinitrobenzoate moiety from our procedure at large scales.

**Table 2:** Thermal Properties Measured by Differential Scanning Calorimetry (DSC)^a^

						Yoshida		Pfizer	
entry	compound	*T*_onset_/°C	*T*_init_/°C	Δ*H*_d_/J g^–1^	Δ*H*_d_/kJ mol^–1^	IS	EP	IS	EP
1	isothiouronium iodide **4**	204	176	209	76	–0.90	–0.83	–0.46	–0.59
2	isothiouronium dinitrobenzoate **6**	293	212	856	383	–0.42	–0.28	0.10	–0.01
3	3,5-dinitrobenzoic acid	378	313	2536	538	–0.03	0.14	0.47	0.41

a DSC measurements made over the range 40–450 °C@10 °C min^–1^ using gold plated stainless steel capsules. *T*_init_ is the temperature at which the normalized heatflow exceeds 0.01 W g^–1^ above the baseline; *T*_onset_ is the intersection of the maximum peak gradient and the baseline; Δ*H*_d_ is the integral area of the exotherm peak; IS (impact sensitivity) and EP (explosive propagation) calculated according to Yoshida^47^ or Pfizer.^48^

### Development of S-Deprotection/S_N_Ar Thioetherification

Having developed scalable conditions for the synthesis of isothiouronium iodide **4.solv** in reproducibly high yield and purity, our final objective was to develop a robust method for its deprotection/S-arylation. This would ultimately yield thioether **1** and therefore intercept the patent route^38^ to tozasertib without the need to isolate the free thiophenol.

Initial attempts were made to perform the S_N_Ar arylation under conditions similar to the original patent literature, but with the inclusion of base to mediate the deprotection of isothiouronium salt **4**. However, both *in situ* and *a priori* deprotection/S_N_Ar failed to return thioether **1**, instead forming *N*-arylated isothiourea **7** as the only identifiable product (Scheme 6A,B).

**Scheme 6:**
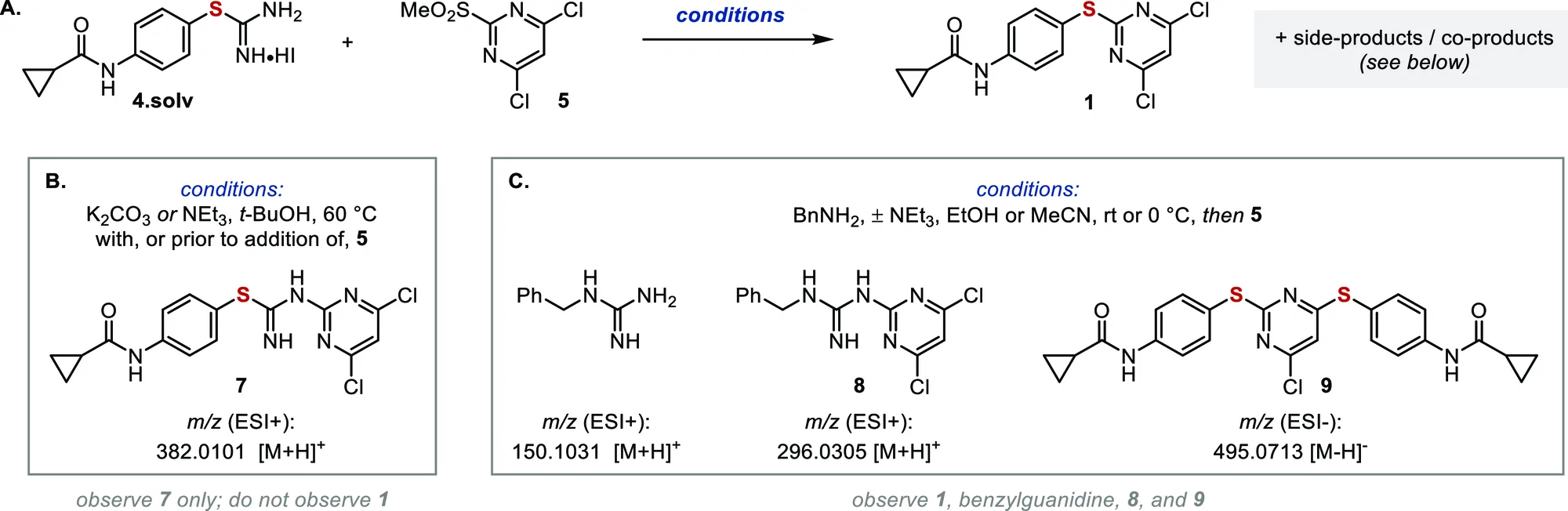
Summary of Initial Deprotection/S_N_Ar Studies (A), and Major Observed Side-Products (B, C)

The apparent stability of the free base of isothiouronium salt **4** in *t*-BuOH prompted a more detailed investigation of the S-deprotection step (Table 3), independent of the subsequent S_N_Ar. Of note, samples of thiophenol **2** isolated during these S-deprotection studies proved sensitive to aerobic oxidation, forming the corresponding disulfide both in solution and in the solid state. This propensity for oxidation highlights a key practical disadvantage of using free thiophenols in synthesis, and hence the benefit of using isothiouronium salts as bench-stable alternatives.

**Table 3:**
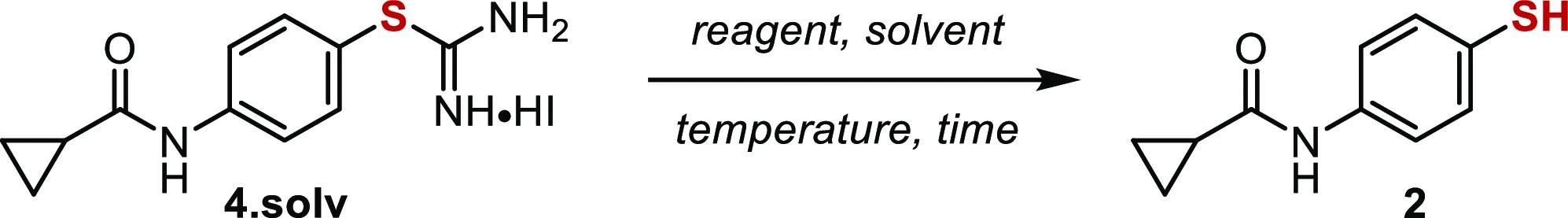
Investigation of Thiophenol Deprotection^a^

entry	reagent (equiv)	solvent	[**4**]_0_/M	temp./°C	time	% deprotection
1	NaOH (2)	*i*-PrOH	0.5	rt or 60	20 h	nd
2	NaBH_4_ (2)	EtOH	0.5	rt	4 h	80
3	BnNH_2_ (1)	EtOH	0.5	50	15 min	>95
4	BnNH_2_ (1)	EtOH	0.25	50	15 min	>95
5	BnNH_2_ (1)	EtOH	0.25	rt	15 min	>95
6	NaOH (3)	water	0.25	rt	15 min	>95

a % Deprotection determined by ^1^H NMR spectroscopic analysis as the conversion of isothiouronium iodide **4** to thiophenol **2** and the corresponding disulfide, which is formed during sampling and analysis under aerobic atmosphere.

Attempts to effect base-mediated fragmentation^49^ of the isothiouronium motif using NaOH in *i*-PrOH proved unsuccessful (Entry 1), similar to initial studies in *t*-BuOH. A reductive deprotection with NaBH_4_ ^50^ was more promising (Entry 2), but was ultimately disregarded due to the potential safety risks associated with hydrogen gas evolution at larger scales.^51^ In contrast, treatment of isothiouronium iodide **4.solv** with benzylamine resulted in rapid and quantitative conversion to thiophenol **2** (Entries 3–5). The efficacy of these reappropriated guanylation conditions^52^ as a method for S-deprotection^53^ presumably stems from the accompanying formation of benzylguanidine: this serves to sequester cyanamide and therefore drive the otherwise reversible^49^ isothiourea fragmentation to completion. However, all attempts to telescope the benzylamine-mediated deprotection with subsequent S_N_Ar on pyrimidine sulfone **5** returned *N*-arylated guanidine **8** and double addition product **9** alongside desired thioether **1** (Scheme 6A,C).

Finally, treatment of isothiouronium iodide **4.solv** with aqueous sodium hydroxide at room temperature furnished the sodium salt of **2** (Table 3, Entry 6), which precipitated quantitatively from solution in under 15 min. Due to their simplicity and efficacy, these S-deprotection conditions were carried forward for development of a telescoped S-deprotection/S_N_Ar process.

To this end, a cyclohexane wash of the aqueous thiolate slurry, followed by the addition of acetone, gave a solution (Scheme 7A). Prewashing the slurry with cyclohexane was found to remove aliphatic impurities—presumably derived from the GVL and/or *i-*PrOAc present in **4.solv**—which were otherwise hard to purge downstream. Addition of the resulting thiolate solution to pyrimidine sulfone **5** gave thioether **1**, which precipitates directly from the reaction. Selectivity for thioether **1** over side-products **9**–**11** (Scheme 7B) was achieved by (a) controlling the rate and temperature of the addition, (b) using a slight excess of pyrimidine sulfone **5**, and (c) selecting a solvent mixture that promoted precipitation of the desired product from the reaction mixture. While using pyrimidine sulfone **5** in excess also served to ensure full conversion of the thiolate nucleophile, less conservative stoichiometries resulted in significant competition from direct reaction of **5** with hydroxide (→**12**) or cyanimide (→**13**). Notably, attempts to suppress these side-reactions by using a lower initial stoichiometry of NaOH resulted in incomplete S-deprotection of isothiouronium salt **4** and incomplete consumption of pyrimidine sulfone **5**, which proved challenging to remove from the final product.

**Scheme 7:**
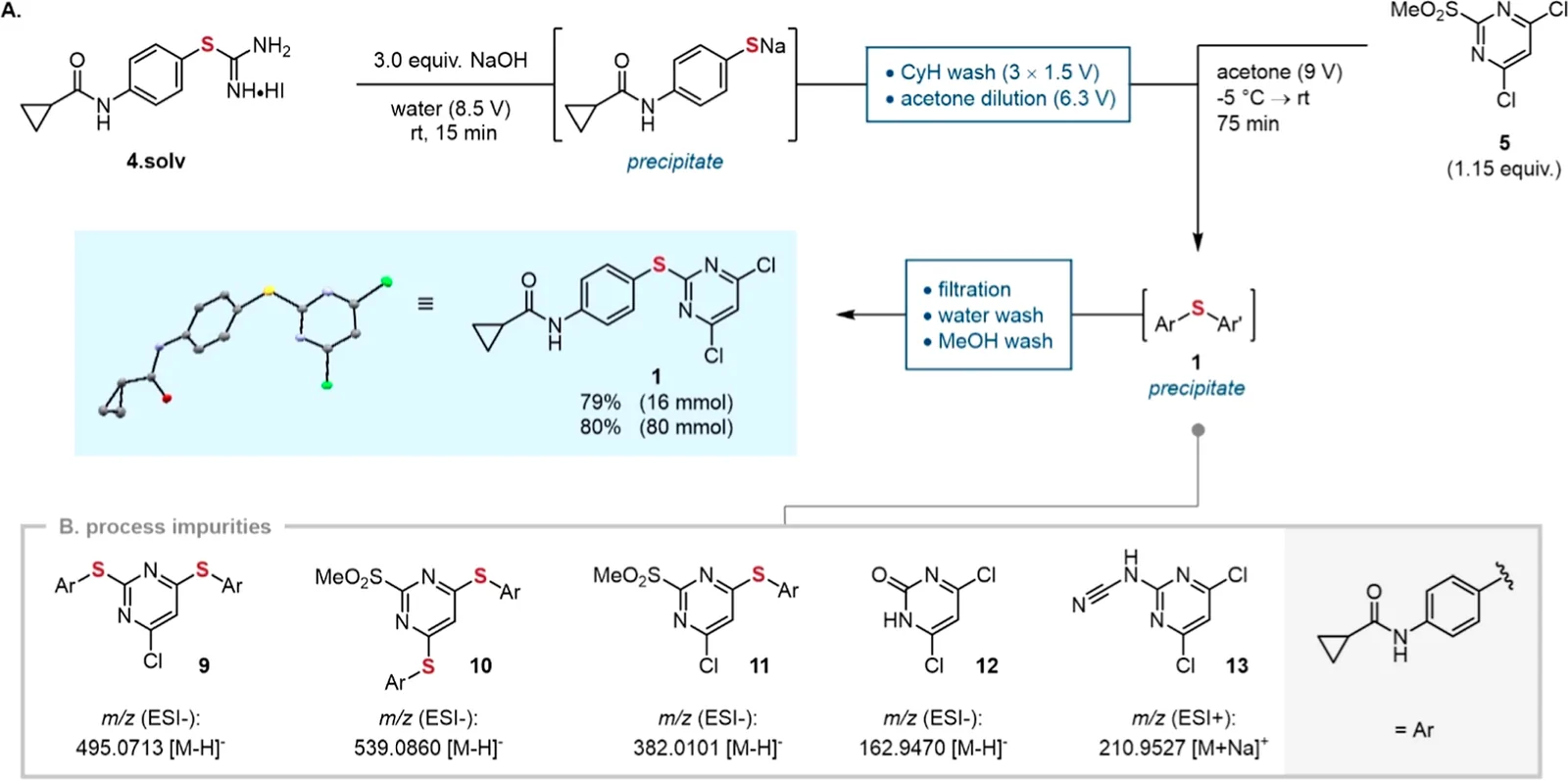
Telescoped Protocol for S-Deprotection/S_N_Ar Arylation of Isothiouronium Iodide **4** ^a^

a Yields refer to pure, isolated material. ORTEP image of **1**: H atoms omitted for clarity; thermal ellipsoids shown at 50% probability.

Isolation of the pure thioether **1** was achieved by filtration of the crude aqueous slurry. Performing both the filtration and the subsequent water wash under a nitrogen atmosphere prevented oxidation of the iodide coproduct, which otherwise led to discoloration of final product **1**. This telescoped deprotection/S_N_Ar process ultimately proved highly reproducible on increasing scales, affording thioether **1** in 79–80% yield (98% purity, as determined by qNMR), which is competitive with the patent yield.

## Conclusions

We have developed a concise route to thioether **1**—a key intermediate in the synthesis of tozasertib (VX-680)—consisting of a Ni-catalyzed C–S coupling and a telescoped S-deprotection/S_N_Ar sequence (Scheme 8). While one step longer than the original patent route, it avoids the need to directly manipulate thiophenols, uses cheaper starting materials,^54^ and gives stable, crystalline intermediates and products in an improved overall yield (73% from 4-iodoaniline vs 64% from 4-aminothiophenol).

**Scheme 8:**
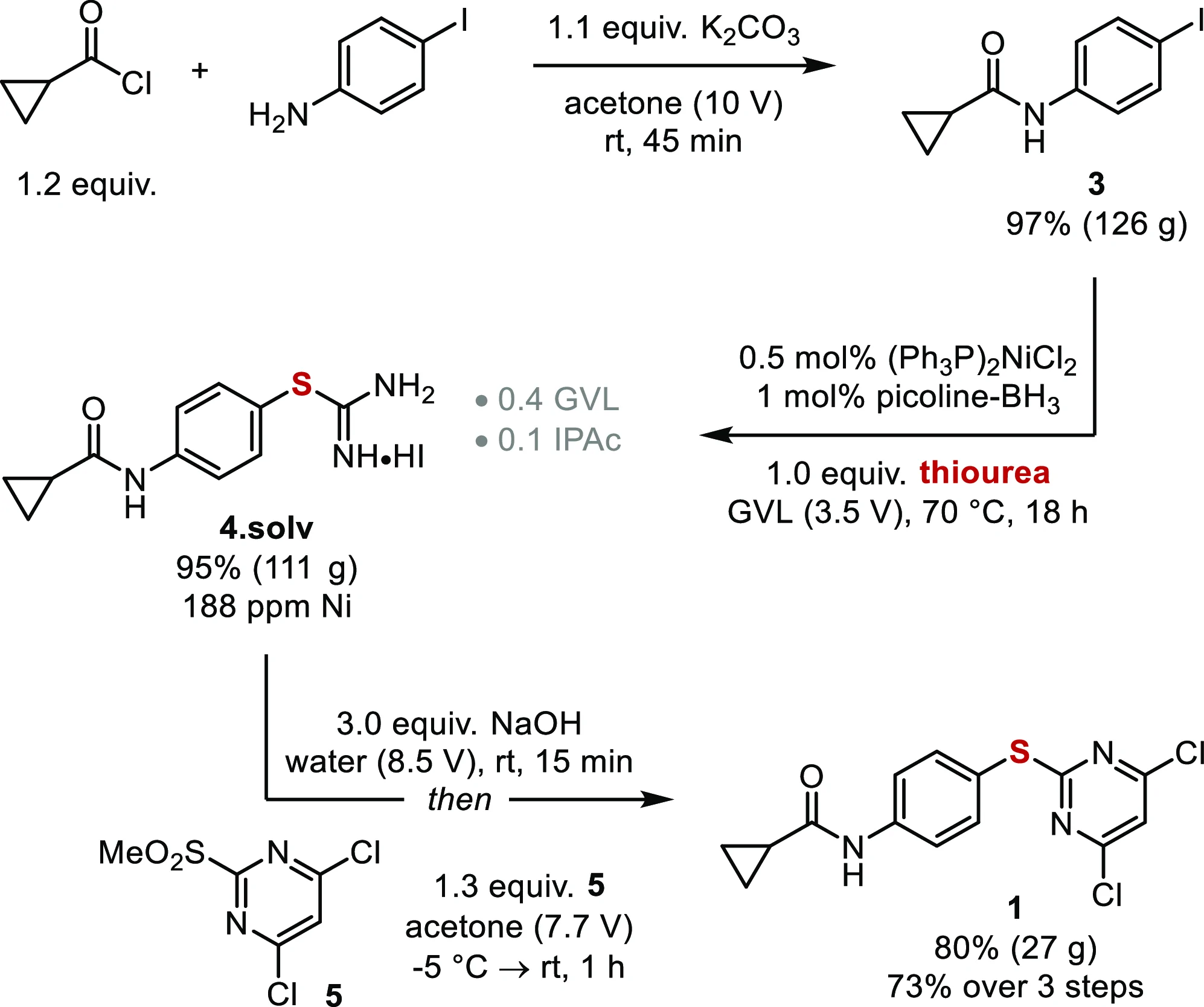
Summary of Route to Thioether **1**

As the main objective of this study, we demonstrated that the Ni-catalyzed coupling of aryl iodides and thiourea is a viable alternative to more conventional Migita-type couplings for process scale thiophenol synthesis. The translation from discovery-oriented methodology to process was informed by Design of Experiments, which allowed identification of robust and high-yielding conditions that operate at a reduced loading of a cheaper precatalyst, and in a more environmentally friendly solvent (GVL vs NMP). Thermal hazard analysis confirmed the safety concerns associated with the iodide-to-nitrobenzoate anion metathesis used in our earlier workflow, prompting development of a direct crystallization which additionally benefited atom efficiency and processability. Together, these changes have allowed for deployment of our C–S coupling methodology on a 100 g scale, giving isothiouronium salt **4** in excellent yield in a green solvent, and with a low loading of base metal catalyst.

The isothiouronium salt **4** produced by this cross-coupling was shown to be a viable pronucleophile for S_N_Ar, which gives key thioether **1** and intercepts the patent route to tozasertib (VX-680). Initial challenges in the deprotection/S_N_Ar sequence arose primarily due to the high reactivity of pyrimidine sulfone electrophile **5**, and are therefore anticipated to be reaction specific. Our observations in this regard highlight important considerations in any future translations of our methodology to novel targets.

## Experimental Section

### General Information

Please see the Supporting Information for details of the reagents, equipment and instrumentation, software, and general procedures employed in this research.

#### *N*-(4-Iodophenyl)­cyclopropanecarboxamide (**3**)

Solid K_2_CO_3_ (69.4 g, 502 mmol) was added to a mechanically stirred solution of 4-iodoaniline (100 g, 456 mmol) in acetone (1.0 L). The reaction vessel was immersed in an ice-water bath to achieve an internal temperature of 20 °C. Cyclopropanecarbonyl chloride (45.6 mL, 502 mmol) was added over 10 min (internal reaction temperature 20 °C → 26 °C), forming a thick suspension after *ca.* 30 mL of the addition. After 45 min, ice water (2.0 L) was added; the suspended solids largely dissolved before a large amount of precipitate formed. The suspension was stirred for 15 min, then the precipitate was collected by vacuum filtration on a sintered glass funnel. The filter cake was washed with water (2× 200 mL), dried under a flow of air, then further washed with cyclohexane (200 mL). Drying under a flow of air afforded the title compound as a microcrystalline colorless solid (126 g, 438 mmol, 97%). Characterization data were consistent with literature values: ^1^H NMR and m.p.^55,56^ ^1^H NMR (400 MHz, CDCl_3_): δ_H_ 7.65–7.61 (m, 2H), 7.32 (app. d, *J* = 8.4 Hz, 2H; overlaid with br. s from amide–H), 1.53–1.47 (m, 1H), 1.13–1.09 (m, 2H), 0.91–0.86 (m, 2H). ^1^H NMR (400 MHz, DMSO-*d*_6_): δ_H_ 10.29 (s, 1H), 7.61 (d, *J* = 8.7 Hz, 2H), 7.43 (d, *J* = 8.8 Hz, 2H), 1.85–1.64 (m, 1H), 0.87–0.71 (m, 4H). ^13^C­{^1^H} NMR (101 MHz, DMSO-*d*_6_): δ_C_ 171.8, 139.2, 137.3, 121.2, 86.2, 14.6, 7.3. HRMS (ESI): *m*/*z* calcd. for C_10_H_10_INONa^+^ [M + Na]^+^ 309.9699, found 309.9698. IR: ν_max_/cm^–1^ = 3280, 2361, 2344, 1649, 1525, 1401. m.p./°C: 220–222.

#### *S*-(4-(Cyclopropanecarboxamido)­phenyl) Isothiouronium Iodide (**4.solv**)

*N*-(4-Iodophenyl)­cyclopropanecarboxamide **3** (80.1 g, 282 mmol, 1.00 equiv), thiourea (21.5 g, 282 mmol, 1.00 equiv), (Ph_3_P)_2_NiCl_2_ (922 mg, 1.41 mmol, 0.50 mol %) and picoline-BH_3_ (302 mg, 2.82 mmol, 1.00 mol %) were added to a 500 mL three-neck round-bottom flask. The flask was equipped with a thermometer, septum, and a gas inlet adapter, then evacuated and backfilled with anhydrous dinitrogen three times. GVL (degassed by sparging with dinitrogen for 30 min; 282 mL, [**3**]_0_ = 1.0 M) was added *via* syringe. The reaction mixture was stirred at 520 rpm for 18 h at 70 °C (internal temperature). The reaction was then allowed to cool to room temperature, and the flask was opened to air atmosphere. A 0.05 mL aliquot of the reaction mixture was diluted with DMSO-*d*_6_, for ^1^H NMR spectroscopic analysis. The extent of conversion was calculated from the relative intensities of the signals of aryl iodide **3** and isothiouronium salt **4** in the ^1^H NMR spectrum. IPAc (560 mL) was added and the mixture was kept at 4 °C overnight. The precipitate was collected by vacuum filtration on a sintered glass funnel, then washed with MTBE (2× 150 mL). Drying under a flow of air, then *in vacuo*, afforded the title compound (110.8 g, 268 mmol, 95%; *M*_w_(solvate) = 413.43) as a 1:0.4:0.1 solvate of **4**/GVL/IPAc as a colorless solid. The w/w purity of **4.solv** was determined by ^1^H qNMR spectroscopic analysis (vs 1,3,5-trimethoxybenzene) to be >99%. X-ray diffraction data and ICP-OES analysis of **4.solv** are presented in the Supporting Information. ^1^H NMR (400 MHz, DMSO-*d*_6_): δ_H_ 10.56 (s, 1H), 8.93 (br. s, 2H), 8.59 (br. s, 2H), 7.81–7.77 (m, 2H), 7.61–7.58 (m, 2H), 1.85–1.79 (m, 1H), 0.88–0.82 (m, 4H). ^13^C­{^1^H} NMR (101 MHz, DMSO-*d*_6_): δ_C_ 172.8, 170.7, 143.0, 137.7, 120.7, 115.5, 15.2, 8.1. HRMS (ESI): *m*/*z* calcd. for C_11_H_14_N_3_OS^+^ [M – I]^+^ 236.0852, found 236.0850. IR: ν_max_/cm^–1^ = 3292, 3247, 3075, 1768, 1721, 1709, 1681, 1614, 1586, 1522, 1484, 1401, 1343, 1312, 1252. M.p./°C: 150–154.

#### *N*-(4-((4,6-Dichloropyrimidin-2-yl)­thio)­phenyl) Cyclopropanecarboxamide (**1**)

All solvents/aqueous reagents were degassed via sparging with anhydrous dinitrogen for a minimum of 1 h before use. Cyclohexane and acetone were degassed using metal needles, deionized water and aqueous 2 M NaOH were degassed using sintered glass filtration sticks. Before transferring degassed material, needles and syringes were purged with dinitrogen thrice. 4,6-Dichloro-2-methanesulfonylpyrimidine **5** was purified as described in Section 1: General Information of the Supporting Information.

##### Vessel 1

A 2.5 L Schlenk tube containing *S*-(4-(cyclopropanecarboxamido)­phenyl) isothiouronium iodide **4.solv** (41.3 g, 100 mmol) and a magnetic stir bar was evacuated and backfilled six times with anhydrous dinitrogen. Degassed aqueous NaOH (2 M; 150 mL, 300 mmol) was added to the Schlenk tube against a flow of dinitrogen over a 6 min period. The resultant slurry was stirred at 100 rpm during the addition, then the stirring was increased to 300 rpm. Degassed deionized water (200 mL) was then added against a flow of dinitrogen over a 10 min period, and the stirring was further increased to 550 rpm for 15 min.

The aqueous slurry of aryl thiolate was then washed with using degassed cyclohexane under inert atmosphere: cyclohexane (60 mL) was added to the Schlenk tube, and the biphasic reaction mixture was stirred at 650 rpm for 1 min. Stirring was then stopped to allow phase separation, before the cyclohexane layer was removed using a dinitrogen-flushed needle and syringe (Figure S5A). This process was performed a further two times. Degassed acetone (260 mL) was then added to the Schlenk tube, and the stirring rate was increased to 750 rpm. After 15 min all solid had dissolved, and the stirring rate was decreased to 650 rpm (Figure S5B).

##### Vessel 2

A 3 L three-neck RBF containing 4,6-dichloro-2-(methylsulfonyl)­pyrimidine (26.1 g, 115 mmol) and a magnetic stir-bar, and equipped with a 1 L pressure equalizing dropping funnel, thermometer, and septum, was evacuated and backfilled 10 times with anhydrous dinitrogen. Degassed acetone (200 mL) was added against a flow of dinitrogen, and the resulting mixture was stirred at 250 rpm until a solution was formed. The solution was cooled to an internal temperature of between −4 and −10 °C using an acetone/ice cooling bath with an external temperature of between −15 and −10 °C (Figure S5C). The thiolate solution from vessel 1 was transferred to the pressure equalizing dropping funnel using a dinitrogen flushed needle and syringe, before being added to the 4,6-dichloro-2-(methylsulfonyl)­pyrimidine solution over an 80 min period. Due to precipitation of the product during thiolate addition (Figure S5D), the stirring rate was increased to 350 rpm at 20 min, then 450 rpm at 30 min and 550 rpm at 75 min. Following complete addition of the thiolate solution, the reaction mixture was stirred in the cooling bath for a further 5 min. The cooling bath was then removed and the reaction mixture was left to stir at room temperature. After 35 min the recorded internal temperature was 5 °C, which increased to 15 °C after 75 min. To maximize product precipitation (Figure S5E), degassed water (300 mL) was added over a 10 min period, raising the internal temperature to 20 °C. The reaction mixture was stirred for a further 10 min.

##### Vessel 3

A 1 L two-neck RBF fitted with an in-line sintered glass filter stick was evacuated and backfilled 10 times with anhydrous dinitrogen. The reaction mixture was then transferred from vessel 2 to the in-line filter *via* PTFE cannula (Figure S5F). Degassed water (2× 50 mL) was added to the reaction vessel, then transferred *via* cannula to wash the material on the filter stick. Additional degassed deionized water (50 mL) was used to wash the material on the filter stick directly. The filter stick was then exposed to air and washed with MeOH (3× 50 mL, precooled to 0 °C). The solid material was then dried *in vacuo* at 40 °C to give the title compound (27.3 g, 80 mmol, 80%), as a colorless solid. The w/w purity of **1** was determined by ^1^H qNMR spectroscopic analysis (vs 1,3,5-trimethoxybenzene) to be 98%. ICP-OES analysis of **1** is discussed in the Supporting Information.

#### Characterization Data were Consistent with Literature Values

^13^C­{^1^H} NMR.^35^ ^1^H NMR (400 MHz, DMSO-*d*_6_): δ_H_ 10.46 (s, 1H), 7.75–7.67 (m, 3H), 7.56–7.49 (m, 2H), 1.86–1.72 (m, 1H), 0.87–0.80 (m, 4H). ^13^C­{^1^H} NMR (101 MHz, DMSO-*d*_6_): δ_C_ 172.9, 172.1, 161.2, 141.1, 136.0, 119.9, 119.6, 117.3, 14.7, 7.5. HRMS (ESI): *m*/*z* calcd. For C_14_H_11_Cl_2_N_3_OS^+^ [M + H]^+^: 340.0073, found 340.0061. IR: ν_max_/cm^–1^ = 3334, 3030, 2973, 1747, 1657, 1592, 1522, 1408, 1364, 1269, 1218, 1199, 1101, 960, 846, 830, 816, 675. M.p./°C: 204–208.

## Supplementary Material



## Notes

Experimental procedures, characterization data, and copies of NMR spectra (PDF)
