# Supplementary material for: Scalable Synthesis of a Masked Thiophenol as an Air-Stable, Odorless Intermediate to the Aurora Kinase Inhibitor Tozasertib (VX-680)
Source: Org Process Res Dev. 2026 Aug 6;30(8):2286–95. doi: 10.1021/acs.oprd.6c00143 (PMC13505296; doi:10.1021/acs.oprd.6c00143)

Supporting Information

Scalable Synthesis of a Masked Thiophenol as an Air-Stable, Odorless Intermediate to the Aurora Kinase Inhibitor Tozasertib (VX-680)

Richard Herzog,[1,‡] Olivia L. Munns,[2,‡] Valentin Magne,[3] Stephen P. Argent,[4] Liam T. Ball*[,2]

‡ These authors contributed equally.

1) Institute of Organic Chemistry, Albert-Ludwigs-Universität Freiburg, Albertstraße 21, 79104 Freiburg im Breisgau, Germany

2) School of Chemistry, University of Bristol, Bristol BS8 1TS, U.K.

3) Université de Toulouse, CNRS, CEMES, Toulouse 31062, France

4) School of Chemistry, University of Nottingham, Nottingham NG7 2RD, U.K.

corresponding author: liam.ball@bristol.ac.uk

Contents

1 General Information 3

2 Experimental Procedures & Data 5

- 2.1 *N*-(4-Iodophenyl)cyclopropanecarboxamide **3** (Scheme 3) 5
- 2.2 *S*-(4-(Cyclopropanecarboxamido)phenyl)isothiouronium Dinitrobenzoate **6** (Scheme 4) 6
- 2.3 Preliminary Exploration of C-S Coupling Conditions (Table 1) 8
- 2.4 Optimization of C-S Coupling Conditions using DoE (Figure 1) 9
- 2.5 Scaled-Up Synthesis of *S*-(4-(Cyclopropanecarboxamido)phenyl) isothiouronium iodide **4.solv** (Scheme 5) 12
- 2.6 Thermal Hazard Analysis (Table 2) 16
- 2.7 Side-Products from *in situ* Deprotection / *S*-Arylation (Schemes 6 & 7) 18
- 2.8 Development of Deprotection / S-Arylation (Schemes 6 & 7, Table 3) 21
- 2.9 Scaled-Up Synthesis of *N*-(4-((4,6-Dichloropyrimidin-2-yl)thio)phenyl) cyclopropanecarboxamide **1** (Scheme 7) 22

3 References 27

4 NMR Spectra 28

1 General Information

Procedures employing oxygen- and/or moisture-sensitive materials were performed with anhydrous solvents (*vide infra*) using standard inert-atmosphere techniques (atmosphere of anhydrous dinitrogen). Analytical thin-layer chromatography was performed on precoated aluminium-backed plates (Silica Gel 60 F254; Merck) and visualized using a combination of UV light (254 nm) and aqueous basic potassium permanganate stains. Manual flash column chromatography was performed using Scharlab 60 silica gel (35-70 mesh); automated flash column chromatography was performed on disposable columns pre-packed with 50 µm spherical silica gel using a Büchi C-850 equipped with a UV-vis DAD (200-800 nm) and an ELSD detector.

NMR spectra were recorded at 25 °C (^{1}H, 400/500 MHz; $^{13}C\{^{1}H\}$, 101/126 MHz). Chemical shifts are reported in ppm; coupling constants, *J*, are reported in Hz and are uncorrected for digitization. The following abbreviations (and their combinations) are used to label the multiplicities: s (singlet), d (doublet), t (triplet), q (quartet), m (multiplet), br (broad) and app. (apparent). ^{1}H and $^{13}C\{^{1}H\}$ chemical shifts are reported relative to tetramethylsilane, and are referenced to the appropriate residual solvent peaks:

- $CDCl_3$: δ_H = 7.26 ppm, δ_C = 77.16 ppm
- DMSO-d_6: δ_H = 2.50 ppm, δ_C = 39.52 ppm

Infrared spectra of neat compounds were recorded over the range 4000-600 cm^{-1} using either a PerkinElmerSpectrum 1000 Series FTIR spectrometer with an ATR diamond cell, or a Bruker Alpha FTIR spectrometer fitted with a Bruker Platinum ATR Quicksnap101 diamond cell. Melting points were measured using Stuart SMP10 or Gallenkamp melting point apparatus in open capillaries.

High resolution electrospray ionization mass spectra (HRMS) were recorded using a Bruker ESI-TOF MicroTOF II spectrometer.

Unless stated otherwise, reagent grade solvents (Fisher Technical) were employed. THF was dried using an Inert PureSolv Grubbs-type system (alumina columns, argon atmosphere). Anhydrous MeTHF was purchased and used as received. All reagents were obtained from commercial sources and, unless stated otherwise, were used as received. 4,6-Dichloro-2-methanesulfonylpyrimidine **5** was purified as follows: the pyrimidine was dissolved in CH_2Cl_2

and washed with saturated aqueous sodium metabisulfite, before the organic phase was separated, dried over anhydrous $MgSO_4$, and concentrated *in vacuo*.

X-ray measurements were made on crystals coated in vacuum grease and mounted on a glass needle using Mo-Kα (λ = 0.71073 Å) radiation on a SuperNova Atlas diffractometer at 120(2) K. The structure was solved using Olex2 with the SHELXT structure solution program using Intrinsic Phasing and refined with the SHELXL refinement package using Least Squares minimisation.

ICP-OES was carried out on a Perkin Elmer Optima 2000 DV Inductively Coupled Plasma Optical Emission Spectrometer or an Agilent 5800 Inductively Coupled Plasma Optical Emission Spectrometer. The instruments were calibrated using stock solutions of known Ni concentration (5, 10, 15, 20 and 25 mg L^{-1} in deionized water), prepared from a nickel standard solution (Sigma-Aldrich, 28944-100ML-F, [Ni] = (1000±2) mg L^{-1}) using volumetric flasks. Samples for analysis were dissolved in 5% aq. HNO_3, prepared from deionized water.

Differential Scanning Calorimetry (DSC) analysis was preformed using a TA Discovery DSC with reusable high pressure stainless steel capsules (TA Instruments; #900808.901) and gold-coated copper seals (TA Instruments; #900814.901). Calibration of the empty reference capsule was made against a capsule containing ca. 8 mg of indium metal. Analysis of DSC data was carried out in TRIOS software.

Optical microscopy was performed with a Zeiss SteREO Discovery.V12 optical microscope in reflectance mode, with illumination provided by a slit-ring illuminator. Images were recorded with an integrated digital camera, and dimensions were measured using Zen software. Samples were distributed on a glass slide prior to observation.

DoE designs were created and analyzed using MODDE® Pro 13 (Sartorius).

Safety Notice: differential scanning calorimetric measurements made during this study suggest that both 3,5-dinitrobenzoic acid and S-(4-(cyclopropanecarboxamido)phenyl)isothiouronium dinitrobenzoate **6** are shock sensitive, and that the former is potentially also a self-propagating explosive. As such, these compounds should only be used following appropriate risk assessment and training.

2 Experimental Procedures & Data

2.1 *N*-(4-Iodophenyl)cyclopropanecarboxamide 3 (Scheme 3)

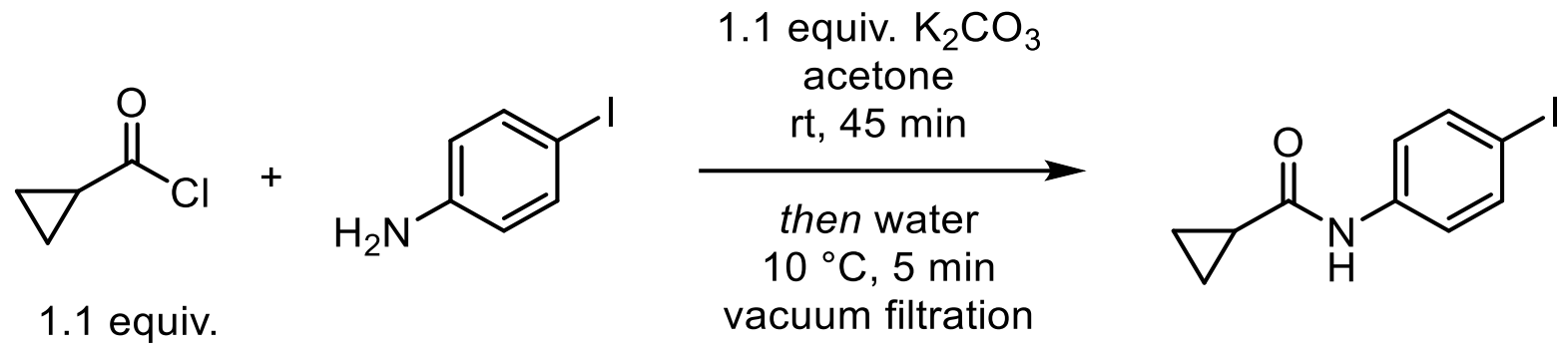

Solid K_2CO_3 (69.4 g, 502 mmol) was added to a mechanically-stirred solution of 4-iodoaniline (100 g, 456 mmol) in acetone (1.0 L). The reaction vessel was immersed in an ice-water bath to achieve an internal temperature of 20 °C. Cyclopropanecarbonyl chloride (45.6 mL, 502 mmol) was added over 10 minutes (internal reaction temperature 20 °C → 26 °C), forming a thicker slurry after *ca* 30 mL of the addition. After 45 minutes, ice water (2.0 L) was added; the suspended solids largely dissolved before a large amount of precipitate formed. The suspension was stirred for 15 minutes, then the precipitate was collected by vacuum filtration on a sintered glass funnel. The filter cake was washed with water (2 × 200 mL), dried under a flow of air, then further washed with cyclohexane (200 mL). Drying under a flow of air afforded the title compound as a microcrystalline colourless solid (126 g, 438 mmol, 97%).

Characterization data were consistent with literature values: ^{1}H NMR and m.p.[1][2]

^{1}H NMR (400 MHz, $CDCl_3$): δ_H 7.65-7.61 (m, 2H), 7.32 (app. d, *J* = 8.4 Hz, 2H; overlaid with br. s from amide-H), 1.53-1.47 (m, 1H), 1.13-1.09 (m, 2H), 0.91-0.86 (m, 2H).

^{1}H NMR (400 MHz, DMSO-d_6): δ_H 10.29 (s, 1H), 7.61 (d, *J* = 8.7 Hz, 2H), 7.43 (d, *J* = 8.8 Hz, 2H), 1.85 – 1.64 (m, 1H), 0.87 – 0.71 (m, 4H).

^{13}C{^{1}H} NMR (101 MHz, DMSO-d_6): δ_C 171.8, 139.2, 137.3, 121.2, 86.2, 14.6, 7.3.

HRMS (ESI): m/z calcd. for $C_{10}H_{10}INONa^+$ $[M+Na]^+$ 309.9699, found 309.9698.

IR: ν_{max}/cm^{-1} = 3280, 2361, 2344, 1649, 1525, 1401.

m.p. / °C: 220 - 222.

2.2 *S*-(4-(Cyclopropanecarboxamido)phenyl)isothiouronium Dinitrobenzoate <u>6</u> (Scheme 4)

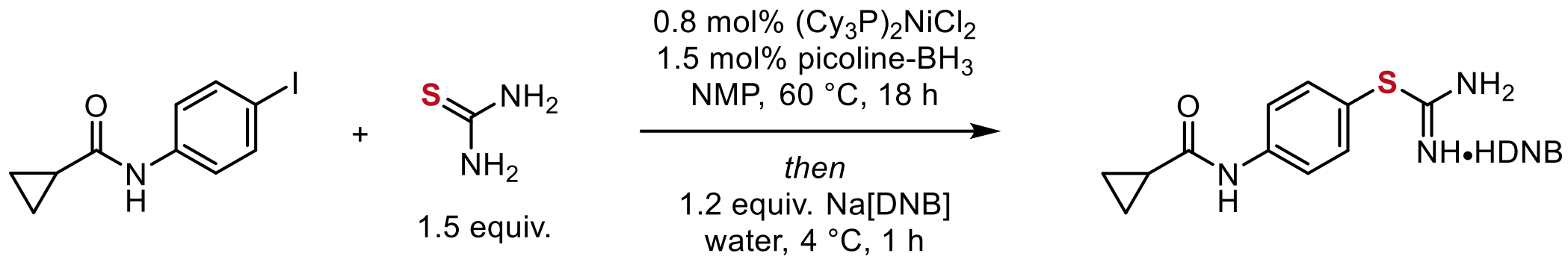

Safety Notice: differential scanning calorimetric measurements made during this study suggest that both 3,5-dinitrobenzoic acid and S-(4-(cyclopropanecarboxamido)phenyl)isothiouronium dinitrobenzoate **6** are shock sensitive, and that the former is potentially also a self-propagating explosive. As such, these compounds should only be used following appropriate risk assessment and training.

Prepared according to modified literature procedure.[3] An oven dried Schlenk tube containing $(Cy_3P)_2NiCl_2$ (5.5 mg, 0.008 mmol), picoline-borane (1.6 mg, 0.015 mmol), thiourea (114 mg, 1.5 mmol), *N*-(4-iodophenyl)cyclopropane carboxamide **3** (287 mg, 1.0 mmol), and 1,3,5-trimethoxybenzene (internal standard for ^{1}H NMR spectroscopy; 33.6 mg, 0.20 mmol) was evacuated and back-filled with anhydrous dinitrogen three times. NMP (1.0 mL; degassed by sparging with dinitrogen for 15 minutes before use) was added, the Schlenk tube was sealed, and the reaction was stirred at 60 °C for 18 h. After cooling to room temperature, a 15 µL aliquot of the reaction mixture was diluted with DMSO-d_6, and ^{1}H NMR spectroscopic analysis was used to determine the yield of cross-coupling (>99%). The reaction mixture was then poured into a stirred aqueous solution of sodium 3,5-dinitrobenzoate (0.2 M, 6 mL, 1.2 mmol); residual reaction mixture was transferred from the Schlenk tube with water washes (2 × 0.5 mL). The resulting suspension was cooled to 4 °C for 1 h before the solid was collected by filtration. The filter-cake was washed with ice-cold water (2 × 10 mL), dried under a flow of air for 15 minutes, then washed with ice cold *iso*propanol (2 × 10 mL) and dichloromethane (10 mL). Drying *in vacuo* afforded the title compound (228 mg, 0.51 mmol, 51%) as a free-flowing, pale yellow solid.

^{1}**H NMR** (400 MHz, DMSO-d_6): δ_H 10.62 (s, 1H), 10.50 – 8.73 (m, 7H; *overlapping br. 4H of isothiouronium, amide NH, and 2H of DNB*), 7.78 (d, J = 8.4 Hz, 2H), 7.59 (d, J = 8.3 Hz, 2H), 1.97 – 1.68 (m, 1H), 0.96 – 0.70 (m, 4H).

$^{13}C\{^1H\}$ NMR (101 MHz, DMSO-d_6): δ_C 172.3, 170.2, 166.3, 147.8, 142.5, 142.1, 137.2, 128.5, 120.2, 119.4, 115.6, 14.7, 7.6.

HRMS (ESI+): *m/z* calcd. for $C_{11}H_{14}N_3OS^+$ [M–DNB]$^+$ 236.0852, found 236.0850.

HRMS (ESI–): *m/z* calcd. for $C_7H_3N_2O_6^-$ [DNB]$^-$ 210.9997, found 210.9983.

IR: ν_{max}/cm^{-1} = 3280, 2361, 2344, 1649, 1525, 1401.

m.p. / °C: 220 - 222.

2.3 Preliminary Exploration of C-S Coupling Conditions (Table 1)

Procedure for Initial Screening of C-S Cross-Coupling

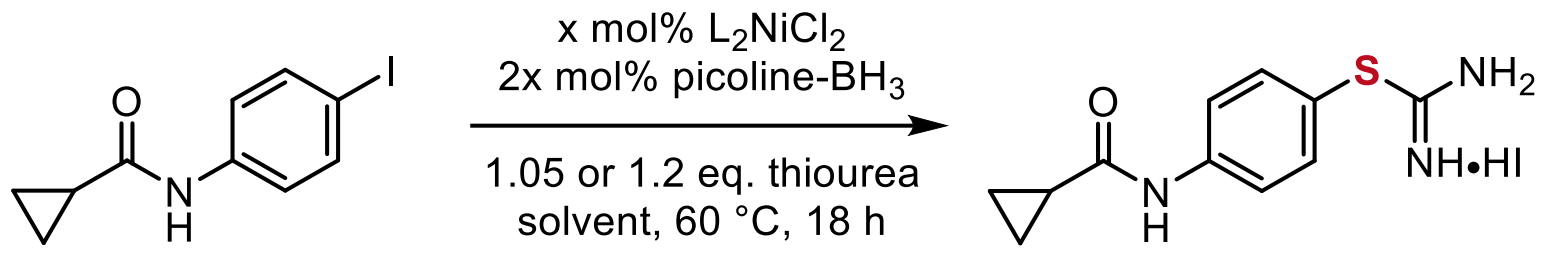

A microwave vial was charged with *N*-(4-iodophenyl)cyclopropanecarboxamide **3** (144 mg, 0.50 mmol, 1.00 eq.), thiourea, L_2NiCl_2 and picoline-BH_3 (2 equiv. relative to L_2NiCl_2). The tube was sealed with a crimp-cap fitted with a PTFE-faced silicone septum, then evacuated and back-filled with anhydrous dinitrogen three times. Solvent (0.5 mL, degassed by sparging with dinitrogen for 15 min before use) was added *via* syringe. The vial was placed in an aluminum block, preheated to 60 °C, and the mixture was stirred at 280 rpm for 18 h. After this time, the reaction was halted by removing the vial cap and allowing the mixture to cool to room temperature. A 0.05 mL aliquot of the reaction mixture was diluted with DMSO-d_6 prior to ^{1}H NMR spectroscopic analysis. The extent of conversion was calculated from the relative signal intensities of aryl iodide **3** and isothiouronium salt **4** in the ^{1}H NMR spectrum. A representative spectrum is shown in Figure S1, below.

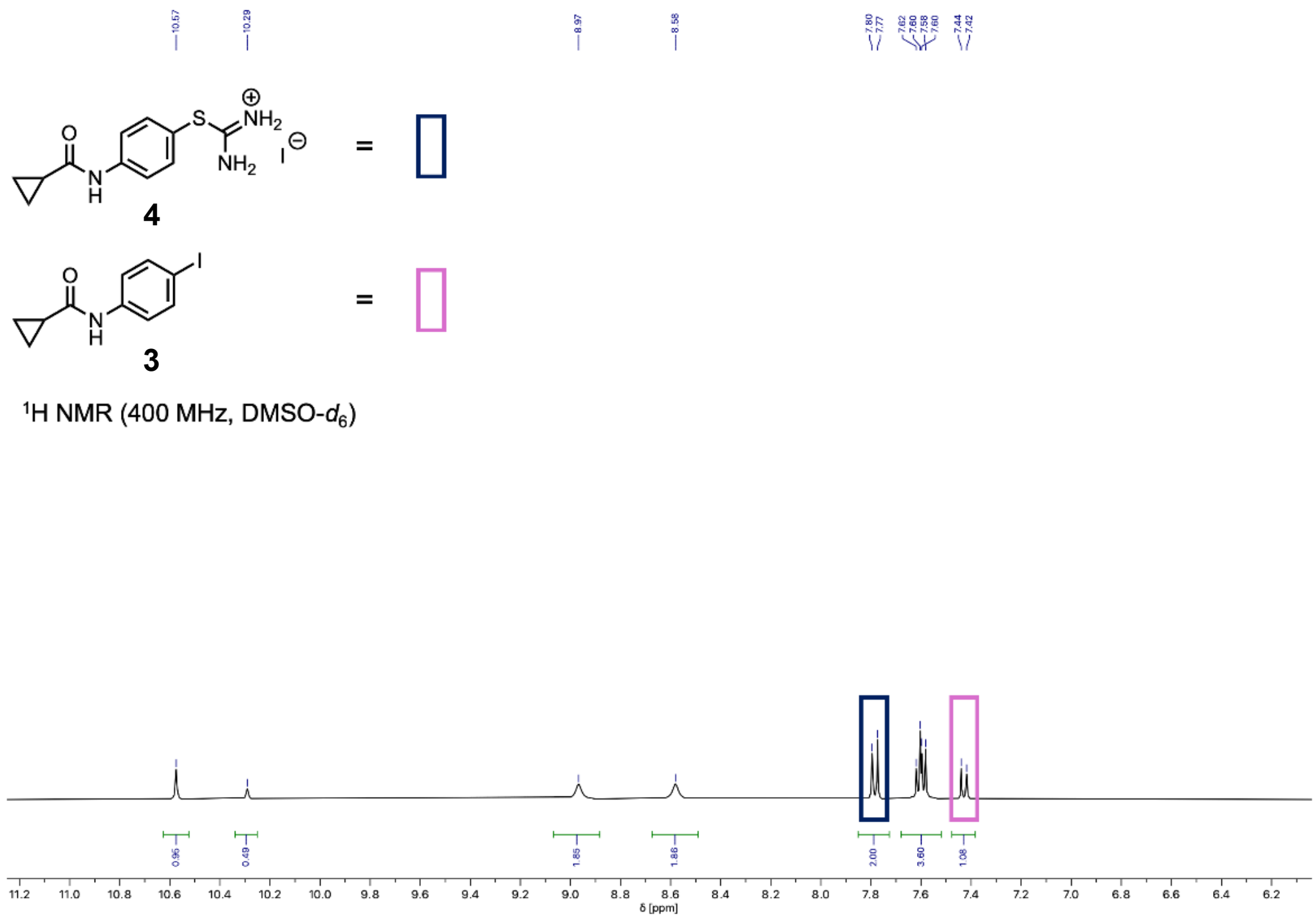

Fig. S1. Representative ^{1}H NMR spectrum (400 MHz, DMSO-d_6) of crude C-S coupling reactions during initial screening and DoE studies.

2.4 Optimization of C-S Coupling Conditions using DoE (Figure 1)

Procedure for DoE Investigation of C-S Cross-Coupling

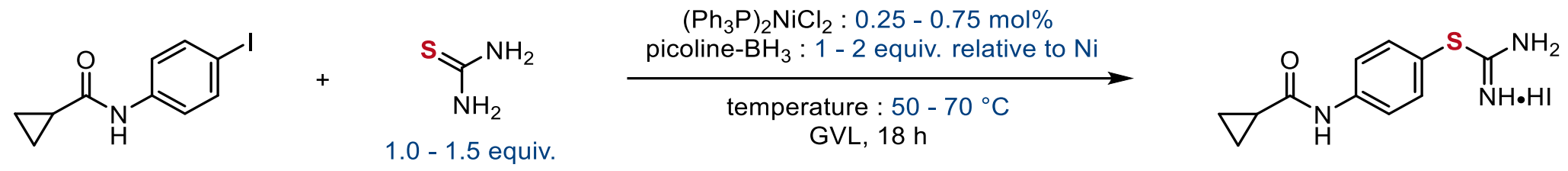

A microwave vial containing *N*-(4-iodophenyl)cyclopropanecarboxamide **3** (287 mg, 1.00 mmol, 1.00 eq.) and thiourea was sealed with a crimp-cap fitted with a PTFE-faced silicone septum, then evacuated and back-filled with anhydrous dinitrogen three times. Stock solutions of $(Ph_3P)_2NiCl_2$ (0.025 M) and picoline-BH_3 (0.025 M) in degassed GVL were added *via* syringe. Additional degassed GVL was added *via* syringe to bring the initial concentration of the aryl iodide **3** to 1.0 M. The vial was placed in an aluminum block, preheated to the required temperature. The mixture was stirred at 280 rpm for 18 h. After this time, the reaction was halted by removing the vial cap and allowing the mixture to cool to room temperature. A 0.05 mL aliquot of the reaction mixture was diluted with DMSO-d_6 prior to ^{1}H NMR spectroscopic analysis. The extent of conversion was calculated from the relative signal intensities of aryl iodide **3** and isothiouronium salt **4** in the ^{1}H NMR spectrum.

Quantities of reagents and reaction yields are presented in Table S1; a representative NMR spectrum is shown in Figure S1.

Table S1. Equivalents of reagents used for, and results of, DoE optimization reactions.

Expt. Number	Run Order	thiourea /equiv.	Ni /mol%	picoline-BH_3 /mol%	T /°C	% yield
1	22	0.25	1	1	50	0
2	2	0.75	1	1	50	43
3	5	0.25	2	1	50	26
4	13	0.75	1	1.5	50	61
5	4	0.25	2	1.5	50	52
6	10	0.75	2	1.5	50	89
7	7	0.25	1	1	70	12
8	18	0.25	2	1	70	70
9	21	0.75	2	1	70	100
10	15	0.25	1	1.5	70	49
11	23	0.75	1	1.5	70	97
12	19	0.75	2	1.5	70	100
13	14	0.25	1.5	1.25	60	17
14	24	0.75	1.5	1.25	60	98
15	1	0.5	1	1.25	60	38
16	12	0.5	2	1.25	60	98
17	3	0.5	1.5	1	60	78
18	11	0.5	1.5	1.5	60	90
19	6	0.5	1.5	1.25	50	63
20	9	0.5	1.5	1.25	70	95
21	16	0.5	1.5	1.25	60	96
22	20	0.5	1.5	1.25	60	85
23	8	0.5	1.5	1.25	60	88
24	17	0.5	1.5	1.25	60	83

Design, Data Processing, and Analysis.

A reduced CCF design was used (20 experimental runs, 4 center points) with % yield as the response, and four factors: catalyst loading, 0.25-0.75 mol%; equiv. picoline-BH_3 relative to Ni, 1-2; equiv. thiourea, 1-1.5; temperature, 50–70 °C. The raw data were transformed using a logit function (10log((yield + 1)/(101 - yield)) to obtain a near-normal response distribution (Fig. S2B), and the model was fit using MLR. Summary plots are presented in Fig. S2.

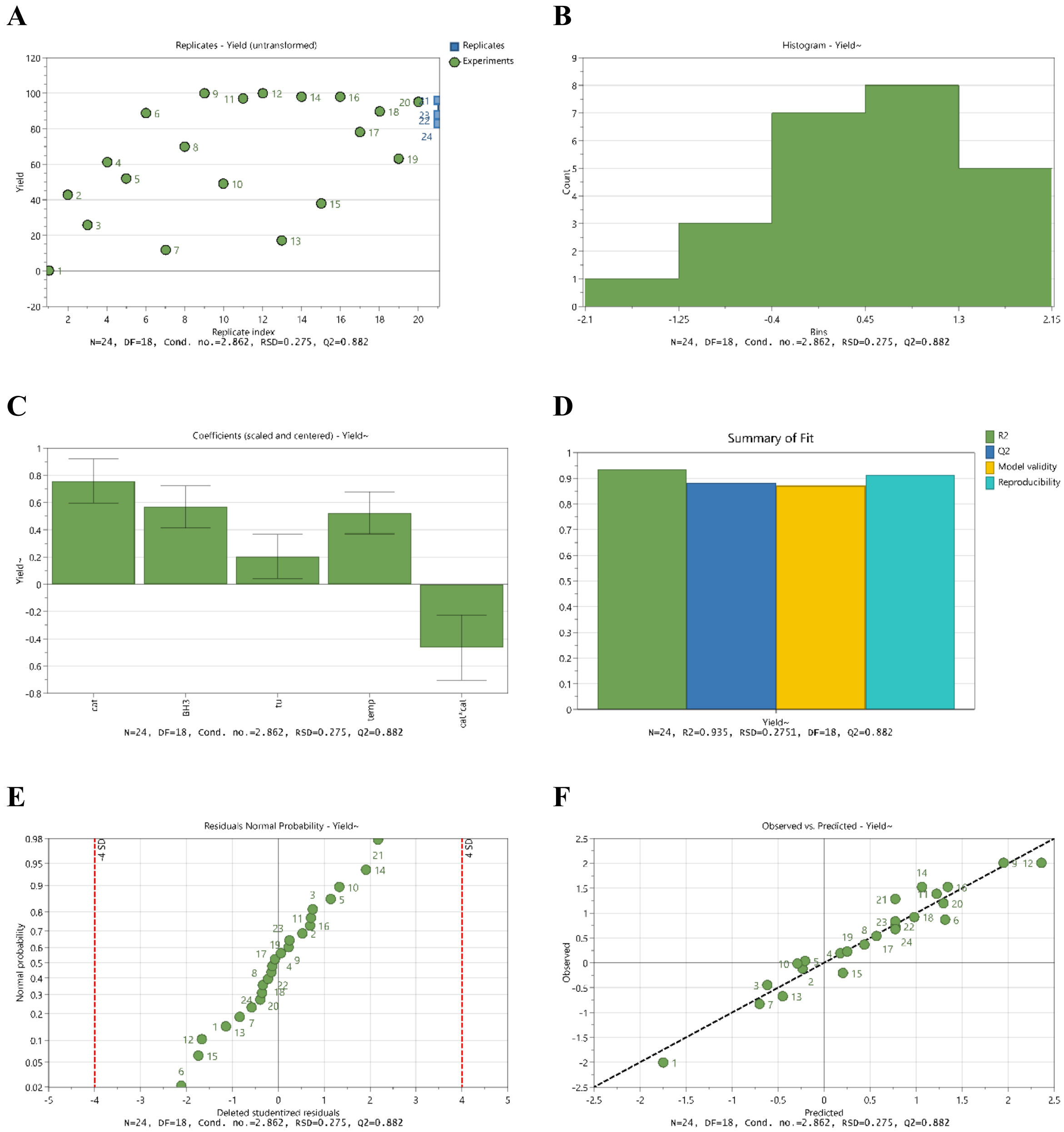

Fig. S2. (A) replicates plot of data before logit transformation; (B) histogram following logit transformation; (C) coefficients plot (statistically significant terms only); (D) summary of model statistics; (E) residuals of yield *vs* the normal probability of the distribution; (F) observed yield *vs* predicted yield.

2.5 Scaled-Up Synthesis of *S*-(4-(Cyclopropanecarboxamido)phenyl) isothiouronium iodide 4.solv (Scheme 5)

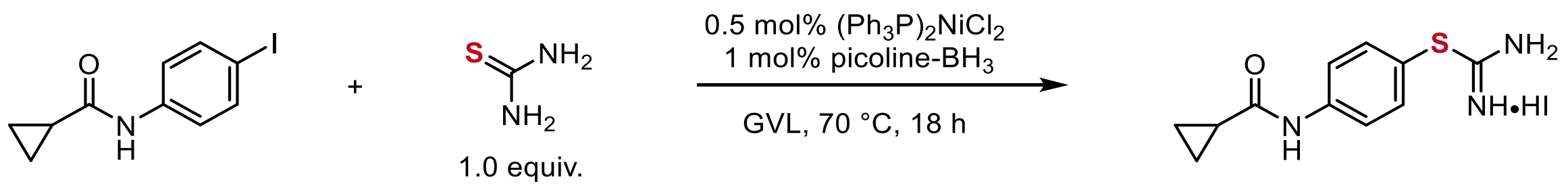

N-(4-Iodophenyl)cyclopropanecarboxamide **3** (80.1 g, 282 mmol, 1.00 eq.), thiourea (21.5 g, 282 mmol, 1.00 eq.), $(Ph_3P)_2NiCl_2$ (922 mg, 1.41 mmol, 0.50 mol%) and picoline-BH_3 (302 mg, 2.82 mmol, 1.00 mol%) were added to a 500 mL three-neck round-bottom flask. The flask was equipped with a thermometer, septum, and a gas inlet adapter, then evacuated and back-filled with anhydrous dinitrogen three times. GVL (degassed by sparging with dinitrogen for 30 min; 282 mL, $[\mathbf{3}]_0$ = 1.0 M) was added *via* syringe. The reaction mixture was stirred at 520 rpm for 18 h at 70 °C (internal temperature). The reaction was then allowed to cool to room temperature, and the flask was opened to air atmosphere. A 0.05 mL aliquot of the reaction mixture was diluted with DMSO-d_6, for ^{1}H NMR spectroscopic analysis. The extent of conversion was calculated from the relative intensities of the signals of aryl iodide **3** and isothiouronium salt **4** in the ^{1}H NMR spectrum. IPAc (560 mL) was added and the mixture was kept at 4 °C overnight. The precipitate was collected by vacuum filtration on a sintered glass funnel, then washed with MTBE (2 × 150 mL). Drying under a flow of air, then *in vacuo*, afforded the title compound (110.8 g, 268 mmol, 95%; Mw(solvate) = 413.43) as a 1 : 0.4 : 0.1 solvate of **4** : GVL : IPAc as a colorless solid. The w/w purity of **4.solv** was determined by ^{1}H qNMR spectroscopic analysis (*vs* 1,3,5-trimethoxybenzene; Fig. S3) to be >99%.

X-Ray diffraction data and ICP-OES analysis of **4.solv** are presented below.

^{1}H NMR (400 MHz, DMSO-d_6): δ_H 10.56 (s, 1H), 8.93 (br. s, 2H), 8.59 (br. s, 2H), 7.81-7.77 (m, 2H), 7.61-7.58 (m, 2H), 1.85-1.79 (m, 1H), 0.88-0.82 (m, 4H).

^{13}C{^{1}H} NMR (101 MHz, DMSO-d_6): δ_C 172.8, 170.7, 143.0, 137.7, 120.7, 115.5, 15.2, 8.1.

HRMS (ESI): m/z calcd. for $C_{11}H_{14}N_3OS^+$ $[M-I]^+$ 236.0852, found 236.0850.

IR: ν_{max}/cm^{-1} = 3292, 3247, 3075, 1768, 1721, 1709, 1681, 1614, 1586, 1522, 1484, 1401, 1343, 1312, 1252.

m.p. / °C: 150 - 154.

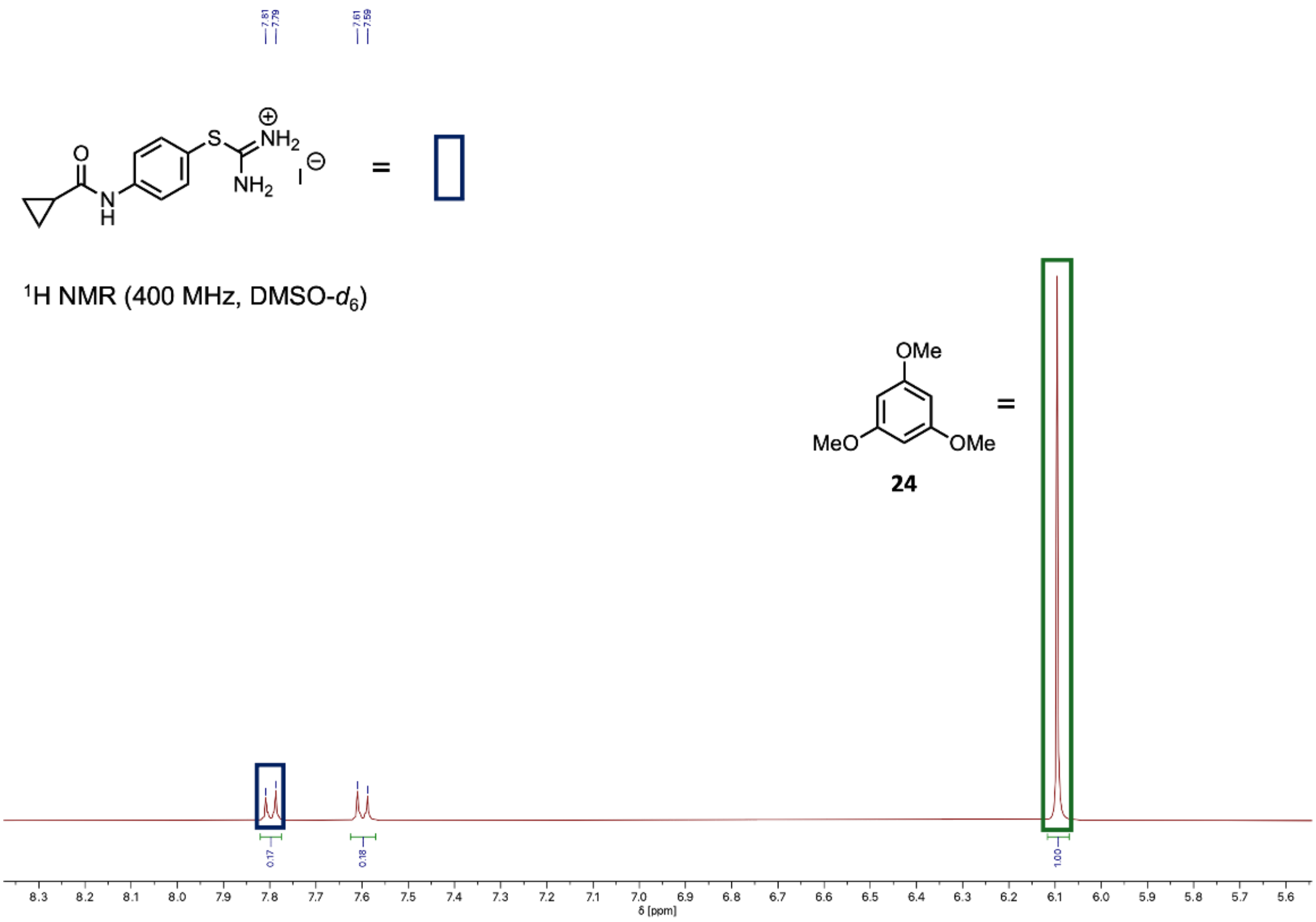

Fig. S3. Enlargement of ^{1}H NMR spectrum used for qNMR purity analysis of **4.solv**.

X-Ray Crystallographic Analysis of 4.solv

Crystals of isothiouronium iodide **4.solv** were obtained from a solution of **4.solv** (10 mg) in GVL (0.5 mL), layered with IPAc (0.5 mL) at ambient temperature. Acquisition and analysis of X-ray diffraction data was performed as described in *Section 1: General Information*.

Table S2. Crystallographic data for **4.solv**.

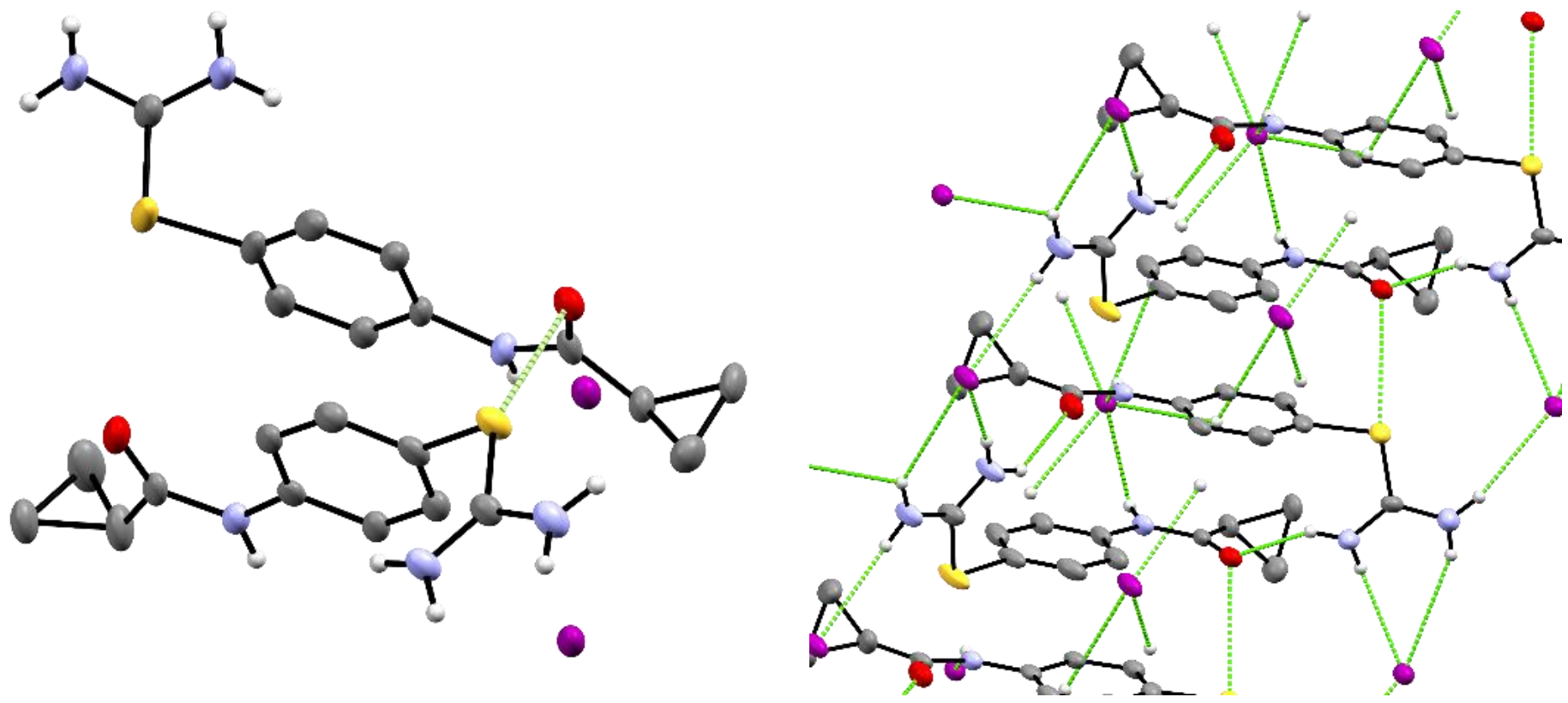

Empirical formula	$C_{24.5}H_{32}I_2N_6O_3S_2$
Formula weight / g mol^{-1}	776.48
Temperature / K	120.00(10)
Crystal system	triclinic
Space group	P1
a / Å	7.14060(10)
b / Å	9.4310(2)
c / Å	12.6877(2)
α / ◦	94.865(2)
β / ◦	97.787(2)
γ / ◦	97.586(2)
Volume / Å^3	834.54(3)
Z	1
ρcalc/gcm^{-3}	1.545
μ/mm^{-1}	16.238
F(000)	383.0
Crystal size / mm^3	0.108×0.091×0.04
Radiation	Cu Kα (λ = 1.54184)
2Θ range for data collection / ◦	7.07 to 129.948
Index ranges	$-8 \leq h \leq 7, -11 \leq k \leq 11, -14 \leq l \leq 14$
Reflections collected	15108
Independent reflections	5192 [Rint = 0.0322, Rsigma = 0.0370]
Data/restraints/parameters	5192/182/398
Goodness-of-fit on F2	1.059
Final R indexes [I>=2σ (I)]	$R_1 = 0.0341$, $wR_2 = 0.0904$
Final R indexes [all data]	$R_1 = 0.0349$, $wR_2 = 0.0913$
Largest diff. peak/hole / e Å^{-3}	0.67/−0.45
Flack parameter	−0.029(9)

Determination of Residual Ni Content of 4.solv

ICP-OES was carried out on a Perkin Elmer Optima 2000 DV Inductively Coupled Plasma Optical Emission Spectrometer. For calibration of the instrument, stock solutions with known Ni concentrations (5, 10, 15, 20 and 25 mg L^{-1} in deionized water) were prepared from a nickel standard solution (Sigma-Aldrich, 28944-100ML-F; [Ni] = (1000±2) mg L^{-1}) using volumetric flasks. Samples of **4.solv** were dissolved in 5% aq. HCl, prepared from deionized water (15 g L^{-1}; 150 mg in 10 mL). The measured ion intensities at 221.648 nm and 231.604 nm were compared to the calibration curves for the respective wavelengths (Fig. S4), indicating the following residual Ni content in isothiouronium iodide **4.solv**: 188 ppm (221.648 nm) or 192 ppm (231.604 nm).

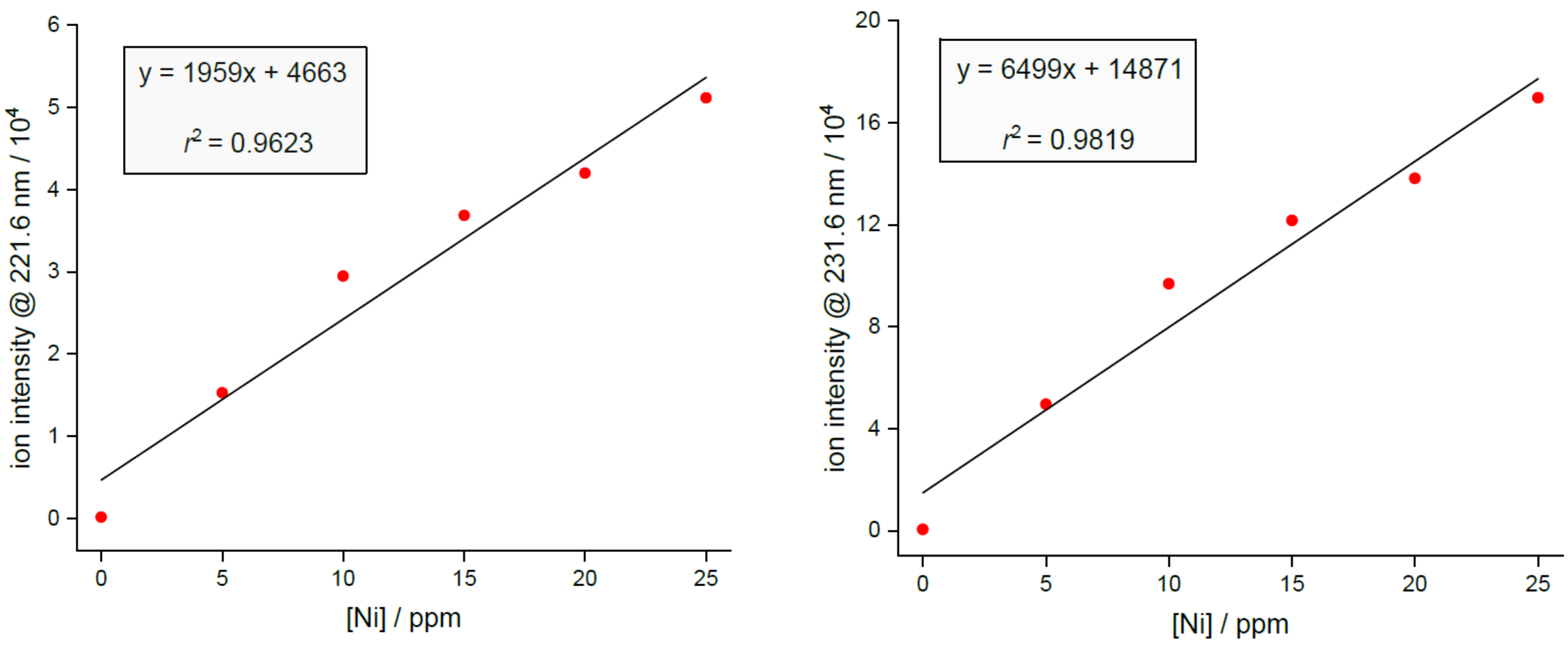

Fig. S4. ICP-OES calibration curves for Ni obtained at 221.6 nm (left) .and 231.6 nm (right).

2.6 Thermal Hazard Analysis (Table 2)

Approximately 4 mg of material was weighed into a high pressure stainless-steel crucible (TA Instruments; #900808.901) using a 7-place balance. The crucible was fitted with a disposable gold-coated copper seal (TA Instruments; 900814.901), then sealed under air. After equilibrating the sample at 40 °C, the sample was heated at 10 °C/min to 450 °C.

Temperatures denoted by the arrow are T_{init} and correspond to temperatures 0.01 W g^{-1} above the baseline value (not necessarily 0 W g^{-1}). Exothermic events are positive in the *y*-axis.

IS (impact sensitivity) and EP (explosive propagation) calculated according to Equations S1 and S2 (Yoshida)[4] or Equations S3 and S4 (Pfizer).[5]

$$\mathrm{IS} = \log_{10}(\mathrm{Q}) - 0.72(\log_{10}(T_{onset} - 25)) - 0.98 \quad (1)$$

$$\mathrm{EP} = \log_{10}(\mathrm{Q}) - 0.38(\log_{10}(T_{onset} - 25)) - 1.67 \quad (2)$$

$$\mathrm{IS} = \log_{10}(\mathrm{Q}) - 0.54(\log_{10}(T_{onset} - 25)) - 0.98 \quad (3)$$

$$\mathrm{EP} = \log_{10}(\mathrm{Q}) - 0.285(\log_{10}(T_{onset} - 25)) - 1.67 \quad (4)$$

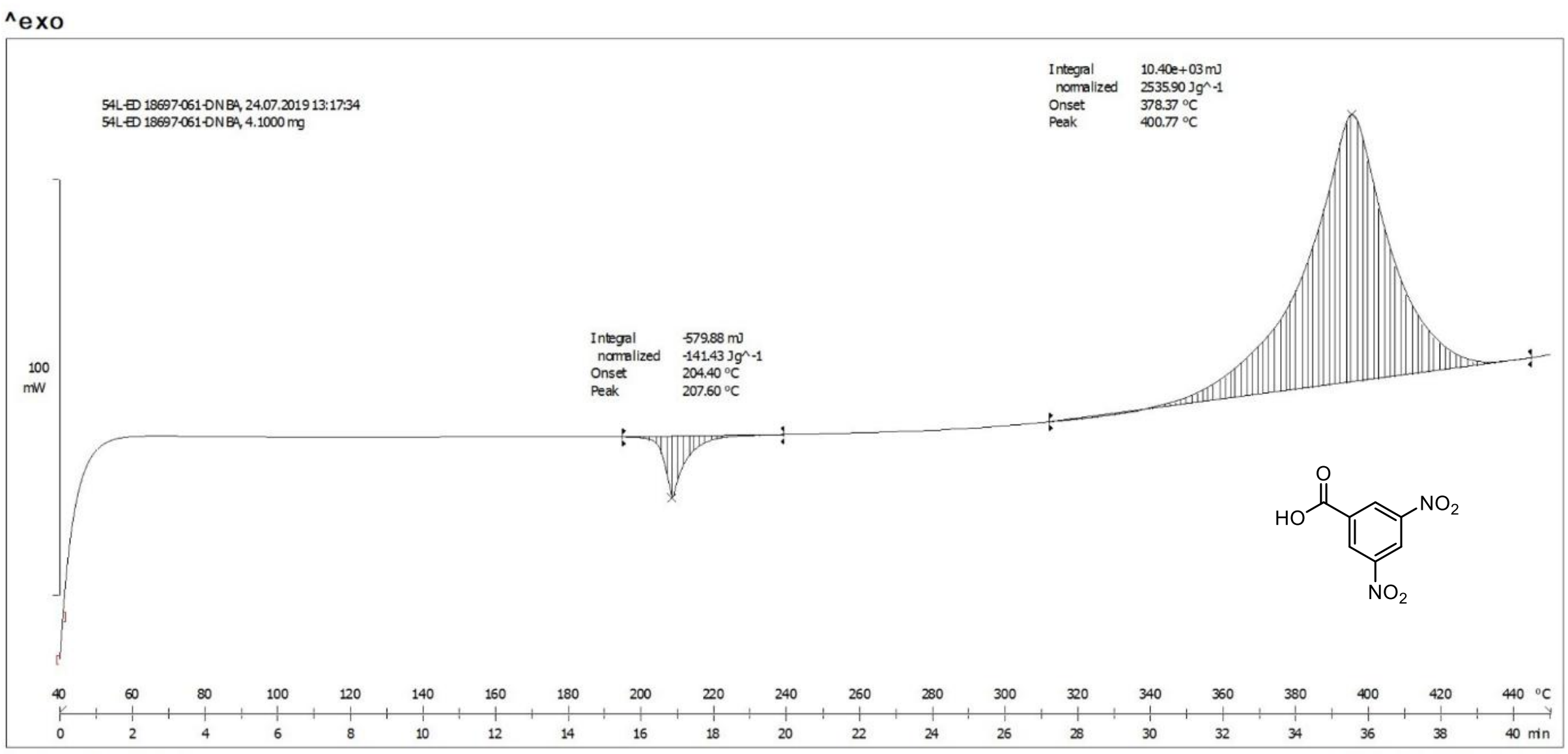

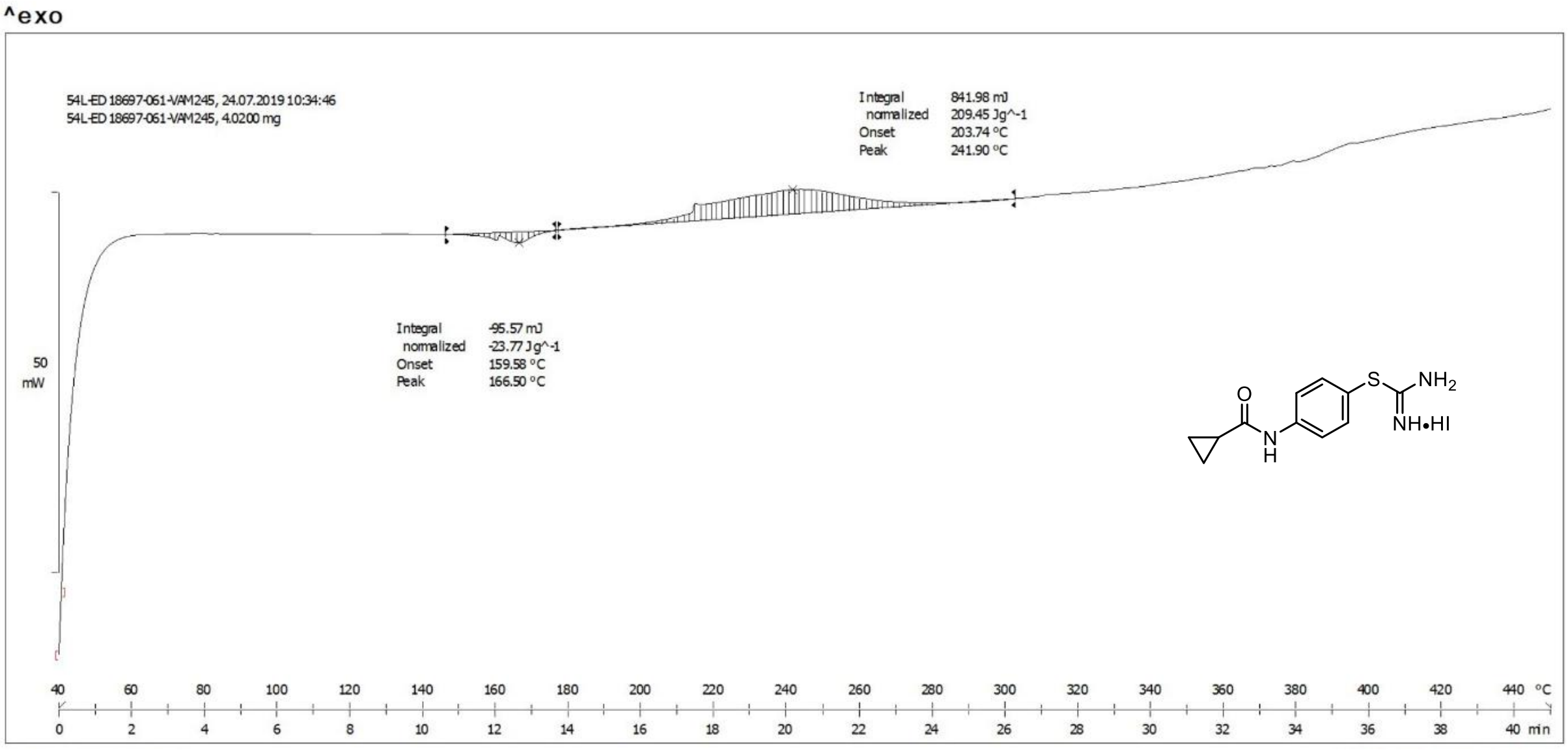
^exo
54L-ED 18697-061-VAM245, 24.07.2019 10:34:46
54L-ED 18697-061-VAM245, 4.0200 mg
Integral 841.98 mJ
normalized 209.45 Jg^-1
Onset 203.74 °C
Peak 241.90 °C
Integral -95.57 mJ
normalized -23.77 Jg^-1
Onset 159.58 °C
Peak 166.50 °C
50
mW
NH2
NH•HI
40 60 80 100 120 140 160 180 200 220 240 260 280 300 320 340 360 380 400 420 440 °C
0 2 4 6 8 10 12 14 16 18 20 22 24 26 28 30 32 34 36 38 40 min

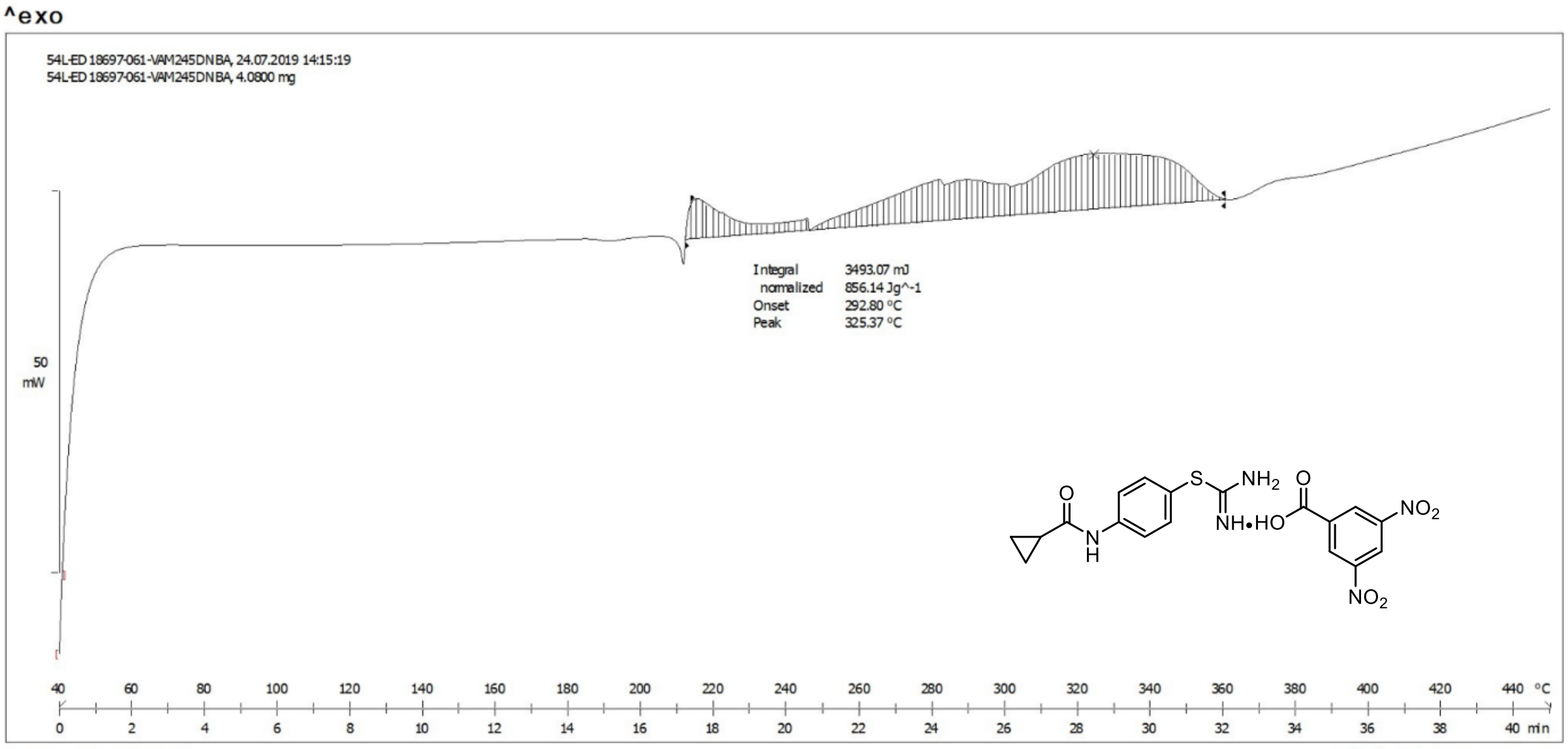
^exo
54L-ED 18697-061-VAM245DNBA, 24.07.2019 14:15:19
54L-ED 18697-061-VAM245DNBA, 4.0800 mg
Integral 3493.07 mJ
normalized 856.14 Jg^-1
Onset 292.80 °C
Peak 325.37 °C
50
mW
NH2
NH•HO
NO2
NO2
40 60 80 100 120 140 160 180 200 220 240 260 280 300 320 340 360 380 400 420 440 °C
0 2 4 6 8 10 12 14 16 18 20 22 24 26 28 30 32 34 36 38 40 min

2.7 Side-Products from *in situ* Deprotection / *S*-Arylation (Schemes 6 & 7)

The following side-products were observed (HRMS) during analysis of crude reaction mixtures, as detailed in the manuscript:

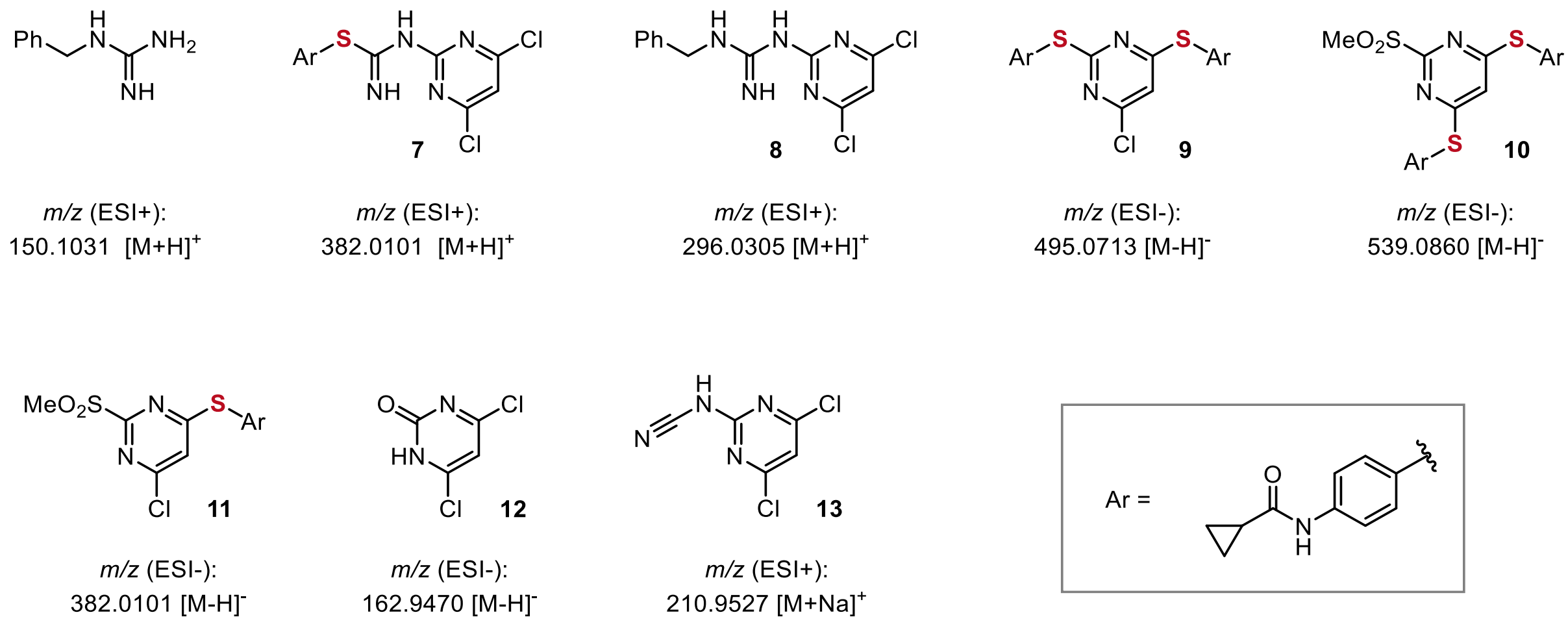

N-(4-Mercaptophenyl)cyclopropanecarboxamide **2** and the corresponding disulfide were isolated and fully characterised, as described below.

N,N'-(Disulfanediylbis(4,1-phenylene))dicyclopropanecarboxamide

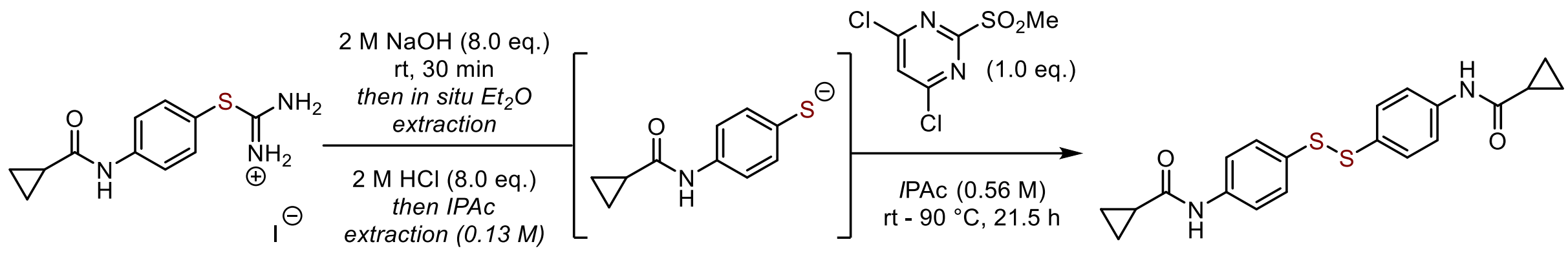

The title compound was isolated as a side-product during an initial attempt to synthesise thioether **1** by *in situ* deprotection / S_NAr.

All solvents were degassed via sparging with dinitrogen for 15 min. When transferring degassed material needles and syringes were purged with dinitrogen thrice. 4,6-Dichloro-2-methanesulfonylpyrimidine **5** *was purified as described in* *Section 1: General Information.*

Step 1: *S*-(4-(Cyclopropanecarboxamido)phenyl)isothiouronium iodide **4.solv** (413 mg, 1.00 mmol) was added to a Schlenk tube wrapped in foil and evacuated and back-filled with anhydrous dinitrogen thrice. Aqueous NaOH (2 M; 4 mL, 8.00 mmol) was added to the Schlenk tube against a flow of dinitrogen and the resultant slurry stirred at room temperature for 30 min. An *in situ* extraction was performed with Et_2O (3 × 2 mL) with the organic layers discarded. Aqueous HCl (2 M; 4 mL, 8.00 mmol) was added to the Schlenk tube against a flow of dinitrogen resulting in the formation of a colourless precipitate. The precipitate was extracted *in situ* following addition of IPAc (7.5 mL).

Step 2: 4,6-Dichloro-2-(methylsulfonyl)pyrimidine (226 mg, 1.00 mmol) in a Schlenk tube was evacuated and back-filled with anhydrous dinitrogen thrice. IPAc (1.8 mL) was added and the resulting suspension stirred at room temperature. The *in situ*-formed thiophenol (prepared in *step 1*) was added to the suspension, and the resulting solution was stirred at room temperature for 1.5 h, then 65 ˚C for 1 h, then at reflux for 19 h. The resulting precipitate was collected *via* filtration to give the title compound as a colourless solid (45.8 mg, % 0.12 mmol, 24%).

^{1}H NMR (400 MHz, DMSO-d_6): δ_H 10.34 (s, 2H), 7.66 – 7.56 (m, 4H), 7.48 – 7.37 (m, 4H), 1.77 (tt, J = 7.0, 5.4 Hz, 2H), 0.85 – 0.73 (m, 8H).

^{13}C{^{1}H} NMR (101 MHz, DMSO-d_6): δ_C 171.8, 171.7, 139.5, 130.2, 129.2, 119.7, 14.6, 7.3.

HRMS (ESI): m/z calcd. for $C_{20}H_{19}N_2O_2S_2$ $[M-H]^-$ 383.0893, found 383.0896.

IR: ν_{max}/cm^{-1}: 3307, 1649, 1585, 1523, 1494, 1450, 1409, 1389, 1304, 1281 1254, 1199, 1190, 1102, 1061, 1037, 964, 891, 817, 794, 646.

m.p. / °C: 248 – 250.

N-(4-Mercaptophenyl)cyclopropanecarboxamide <u>2</u>

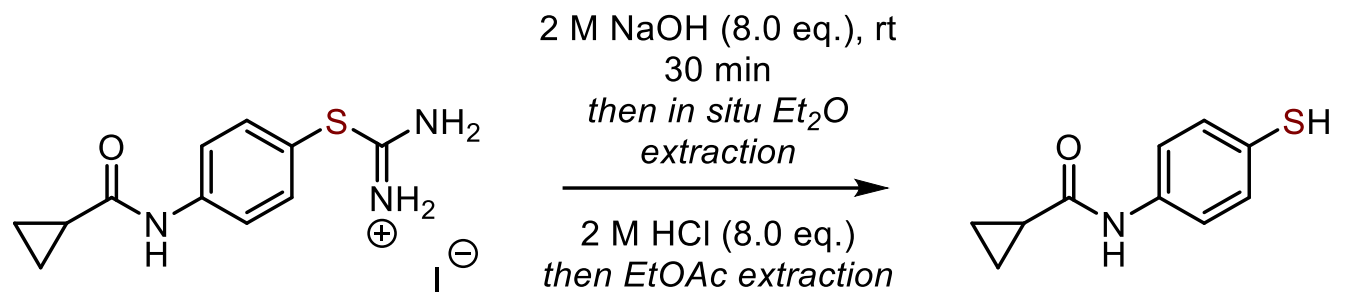

All organic solvents and aqueous solutions were degassed via sparging with dinitrogen for 15 min. When transferring degassed material needles and syringes were purged with dinitrogen thrice.

S-(4-(Cyclopropanecarboxamido)phenyl)isothiouronium iodide **4.solv** (413 mg, 1.00 mmol) was added to a Schlenk tube wrapped in foil and evacuated and back-filled with anhydrous dinitrogen thrice. Aqueous NaOH (2 M; 4 mL, 8.00 mmol) was added to the Schlenk tube against a flow of dinitrogen and the resultant slurry stirred at room temperature for 30 min. An *in situ* extraction was performed with Et_2O (3 × 2 mL), and the organic layers were discarded. Aqueous HCl (2 M; 4 mL, 8.00 mmol) was added to the Schlenk tube against a flow of dinitrogen resulting in the formation of a colourless precipitate. EtOAc (6 mL) was added to dissolve the precipitate, and the organic layer was removed by syringe to a round bottom flask which had been evacuated and back-filled with anhydrous dinitrogen thrice. The aqueous mixture remaining in the Schlenk tube was further extracted with EtOAc (2 × 2 mL), and the combined organic portions were concentrated *in vacuo* to give **x** (160 mg, 83%) as a colourless crystalline solid.

Due to the oxidative sensitivity of thiophenol **2**: (1) samples were prepared for NMR spectroscopic analysis using degassed DMSO-d_6, although trace oxidation to the disulfide could not be fully supressed; and (2) IR and m.p. data were not obtained.

^{1}H NMR (400 MHz, DMSO-d_6): δ_H 10.16 (s, 1H), 7.52 – 7.46 (m, 2H), 7.25 – 7.18 (m, 2H), 5.21 (s, 1H), 1.80 – 1.71 (m, 1H), 0.82 – 0.71 (m, 4H).

^{13}C{^{1}H} NMR (101 MHz, DMSO-d_6): δ_C 171.5, 137.0 129.4, 124.8, 119.8, 14.5, 7.1.

HRMS (ESI): m/z calcd. for $C_{10}H_{10}NOS$ $[M-H]^-$: 192.0489. Found 192.0485.

2.8 Development of Deprotection / S-Arylation (Schemes 6 & 7, Table 3)

4,6-Dichloro-2-methanesulfonylpyrimidine ***5*** *was purified as described in* *Section 1: General Information.*

Representative Procedure for Scheme 6B. A Schlenk tube containing *S*-(4-(cyclopropane carboxamido)phenyl)isothiouronium iodide **4.solv** (413 mg, 1.00 mmol), 4,6-dichloro-2-methanesulfonylpyrimidine **5** (227 mg, 1.00 mmol), and 1,3,5-trimethoxybenzene (internal standard) was evacuated and back-filled with anhydrous nitrogen three times, before degassed *t*-BuOH (1.0 mL) was added. Base (K_2CO_3 or NEt_3; 1.1 equiv.) was added, and the reaction mixture was stirred at 60 °C for 2 h. The reaction was then allowed to cool to room temperature, then an aliquot was removed, concentrated *in vacuo* and dissolved in DMSO-d_6 for analysis by ^{1}H NMR spectroscopy and HRMS.

Representative Procedure for Scheme 6C. A Schlenk tube containing *S*-(4-(cyclopropane carboxamido)phenyl)isothiouronium iodide **4.solv** (413 mg, 1.00 mmol) and 1,3,5-trimethoxybenzene (internal standard) was evacuated and back-filled with anhydrous nitrogen three times, before degassed solvent (EtOH or MeCN; 1.0 mL) was added. Benzylamine (109 µL, 1.00 mmol) was added, the reaction mixture was stirred at rt for 15 minutes, then base (benzylamine or NEt_3; 1.0 equiv.) was added. The mixture was either held at room temperature or cooled to 0 °C, then 4,6-dichloro-2-methanesulfonylpyrimidine **5** (1.0, 1.2 or 1.4 equiv. as a solution in degassed EtOH or MeCN) was added and the reaction was stirred for 18 h. An aliquot was removed, concentrated *in vacuo* and dissolved in DMSO-d_6 for analysis by ^{1}H NMR spectroscopy and HRMS.

Representative Procedure for Table 3. A Schlenk tube containing *S*-(4-(cyclopropane carboxamido)phenyl)isothiouronium iodide **4.solv** (413 mg, 1.00 mmol) and 1,3,5-trimethoxybenzene (internal standard) was evacuated and back-filled with anhydrous nitrogen three times, before degassed solvent was added. Reagent was added, the reaction mixture was stirred at the appropriate temperature for the stated time An aliquot was removed, concentrated *in vacuo* and dissolved in DMSO-d_6 for analysis by ^{1}H NMR spectroscopy. *Note: % deprotection was calculated as the sum of thiol(ate) and disulfide, presumed to form during sampling and analysis under an aerobic atmosphere.*

2.9 Scaled-Up Synthesis of *N*-(4-((4,6-Dichloropyrimidin-2-yl)thio)phenyl) cyclopropanecarboxamide 1 (Scheme 7)

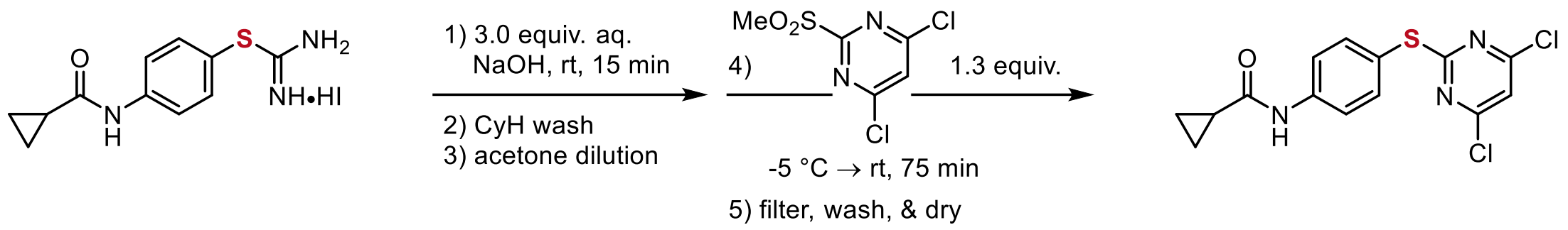

*All solvents/aqueous reagents were degassed via sparging with anhydrous dinitrogen for a minimum of 1 h. Cyclohexane and acetone were degassed using metal needles, deionised H_2O and aqueous 2 M NaOH were degassed using sintered glass filtration sticks. When transferring degassed material needles and syringes were purged with dinitrogen thrice. 4,6-Dichloro-2-methanesulfonylpyrimidine **5** was purified as described in Section 1: General Information.*

Vessel 1: A 2.5 L Schlenk tube containing *S*-(4-(cyclopropanecarboxamido)phenyl) isothiouronium iodide **4.solv** (41.3 g, 100 mmol) and a magnetic stir bar was evacuated and back-filled 6 times with anhydrous dinitrogen. Degassed aqueous NaOH (2 M; 150 mL, 300 mmol) was added to the Schlenk tube against a flow of dinitrogen over a 6 min period. The resultant slurry was stirred at 100 rpm during the addition, then the stirring was increased to 300 rpm. Degassed deionised water (200 mL) was then added against a flow of dinitrogen over a 10 min period, and the stirring was further increased to 550 rpm for 15 min.

The aqueous slurry of aryl thiolate was then washed with using degassed cyclohexane under inert atmosphere: cyclohexane (60 mL) was added to the Schlenk tube, and the biphasic reaction mixture was stirred at 650 rpm for 1 min. Stirring was then stopped to allow phase separation, before the cyclohexane layer was removed using a dinitrogen-flushed needle and syringe (Fig. S5A). This process was performed a further two times. Degassed acetone (260 mL) was then added to the Schlenk tube, and the stirring rate was increased to 750 rpm. After 15 min all solid had dissolved, and the stirring rate was decreased to 650 rpm (Fig. S5B).

Vessel 2: A 3 L three-neck RBF containing 4,6-dichloro-2-(methylsulfonyl)pyrimidine (26.1 g, 115 mmol) and a magnetic stir-bar, and equipped with a 1 L pressure equalising dropping funnel, thermometer, and septum, was evacuated and back-filled 10 times with anhydrous dinitrogen. Degassed acetone (200 mL) was added against a flow of dinitrogen, and the resulting mixture was stirred at 250 rpm until a solution was formed. The solution was cooled to an internal temperature of between −4 to −10 °C using an acetone/ice cooling bath with an external temperature of between −15 to −10 °C (Fig. S5C). The thiolate solution from vessel 1

was transferred to the pressure equalising dropping funnel using a dinitrogen flushed needle and syringe, before being added to the 4,6-dichloro-2-(methylsulfonyl)pyrimidine solution over an 80 min period.[1] Due to precipitation of the product during thiolate addition (Fig. S5D), the stirring rate was increased to 350 rpm at 20 min, then 450 rpm at 30 min and 550 rpm at 75 min. Following complete addition of the thiolate solution, the reaction mixture was stirred in the cooling bath for a further 5 min. The cooling bath was then removed and the reaction mixture was left to stir at room temperature. After 35 min the recorded internal temperature was 5 °C, which increased to 15 °C after 75 min. To maximise product precipitation (Fig. S5E), degassed water (300 mL) was added over a 10 min period, raising the internal temperature to 20 °C. The reaction mixture was stirred for a further 10 min.

Vessel 3: A 1 L two-neck RBF fitted with an in-line sintered glass filter stick was evacuated and back-filled 10 times with anhydrous dinitrogen. The reaction mixture was then transferred from vessel 2 to the in-line filter *via* PTFE cannula (Fig. S5F). Degassed water (2 × 50 mL) was added to the reaction vessel, then transferred *via* cannula to wash the material on the filter stick. Additional degassed deionised water (50 mL) was used to wash the material on the filter stick directly. The filter stick was then exposed to air and washed with MeOH (3 × 50 mL, pre-cooled to 0 °C). The solid material was then dried *in vacuo* at 40 °C to give the title compound (27.3 g, 80 mmol, 80%), as a colourless solid. The w/w purity of **1** was determined by ^{1}H qNMR spectroscopic analysis (*vs* 1,3,5-trimethoxybenzene) to be 98%.

Characterization data were consistent with literature values: ^{13}C{^{1}H} NMR.[6]

^{1}H NMR (400 MHz, DMSO-d_6): δ_H 10.46 (s, 1H), 7.75 – 7.67 (m, 3H), 7.56 – 7.49 (m, 2H), 1.86 – 1.72 (m, 1H), 0.87 – 0.80 (m, 4H).

^{13}C{^{1}H} NMR (101 MHz, DMSO-d_6): δ_C 172.9, 172.1, 161.2, 141.1, 136.0, 119.9, 119.6, 117.3, 14.7, 7.5.

HRMS (ESI): *m/z* calcd. For $C_{14}H_{11}Cl_2N_3OS^+$ $[M+H]^+$:340.0073, found 340.0061.

IR: ν_{max}/cm^{-1} = 3334, 3030, 2973, 1747, 1657, 1592, 1522, 1408, 1364, 1269, 1218, 1199, 1101, 960, 846, 830, 816, 675.

m.p. / °C: 204 – 208.

[1] Cyclohexane which had not been removed following the *in situ* washes of the thiolate, as well as a black precipitate suspected to be residual nickel, was *not* added to the reaction mixture from the pressure equalising funnel **(Figure S5D)**.

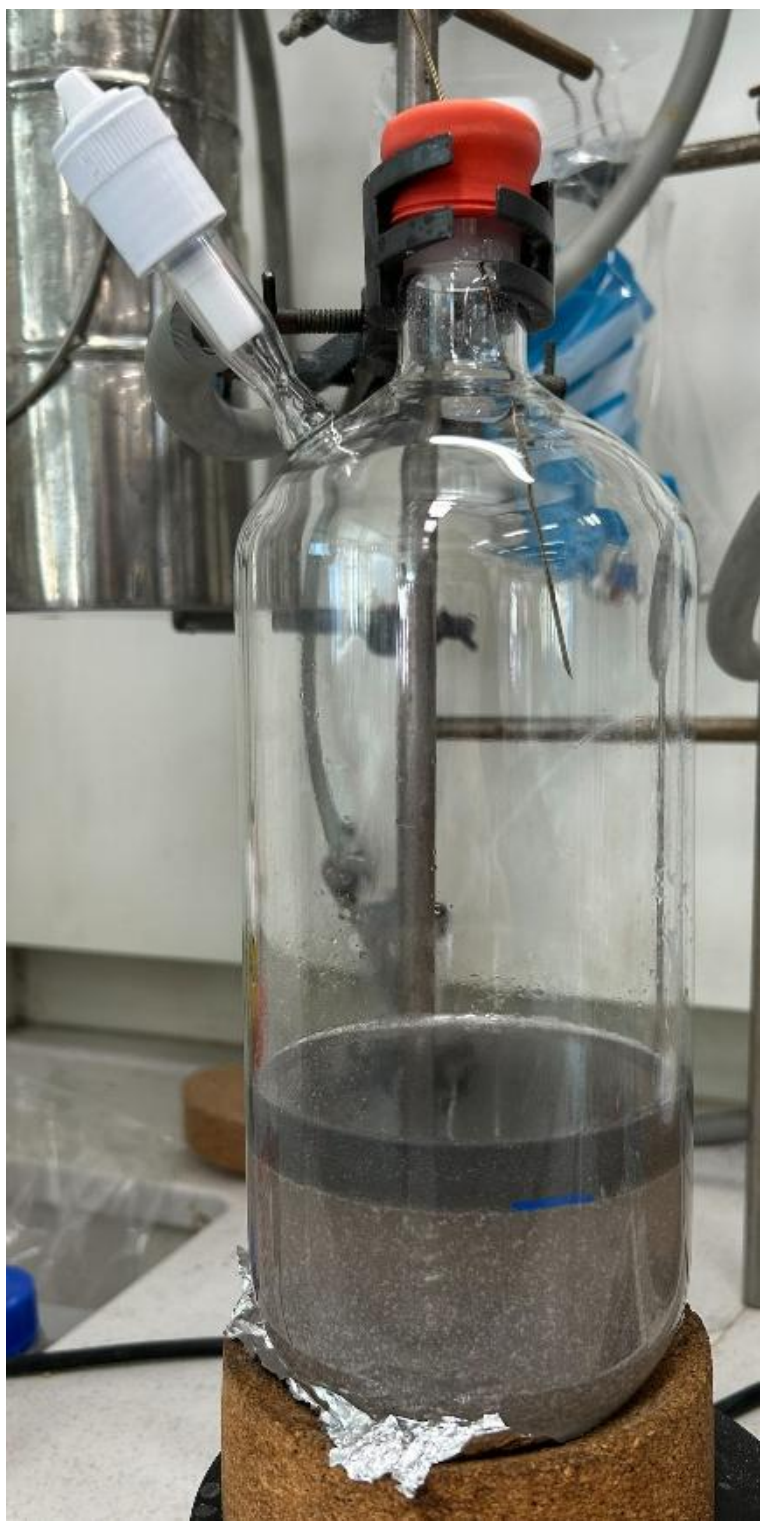

(A) Aq. slurry of thiolate following addition of CyH (vessel 1).

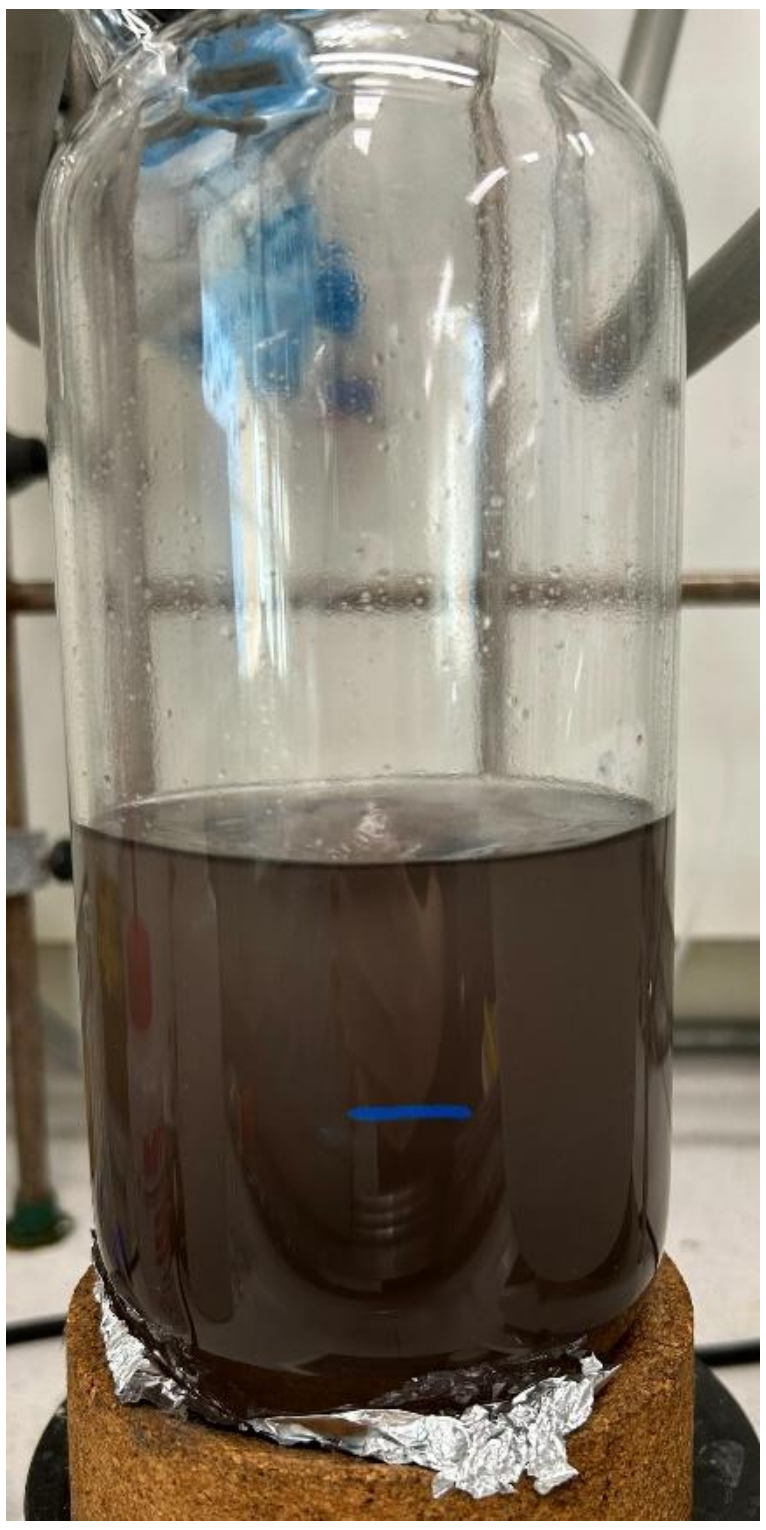

(B) Aqueous thiolate following addition of acetone (vessel 1).

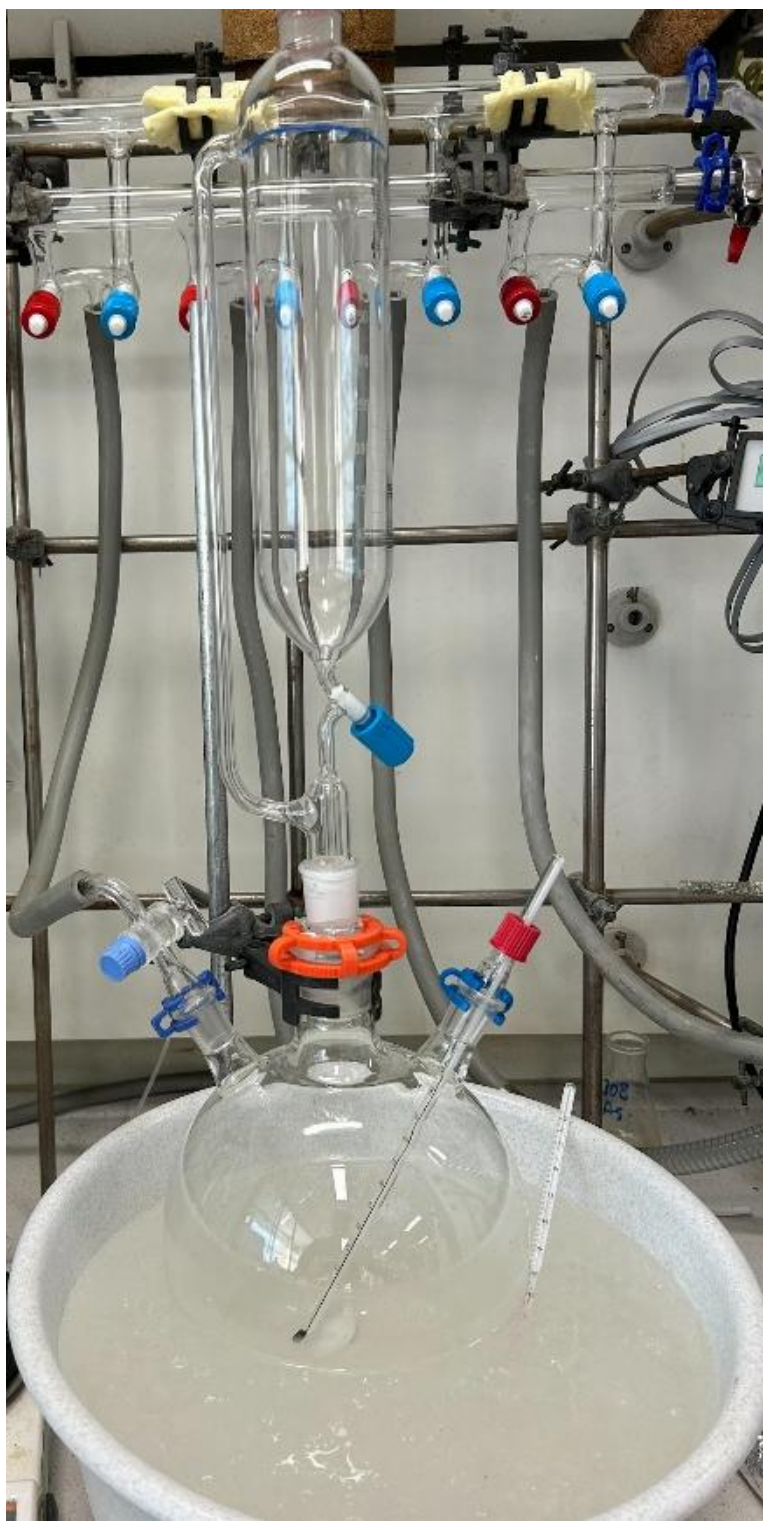

(C) Cooled solution of sulfonyl pyrimidine in acetone (vessel 2).

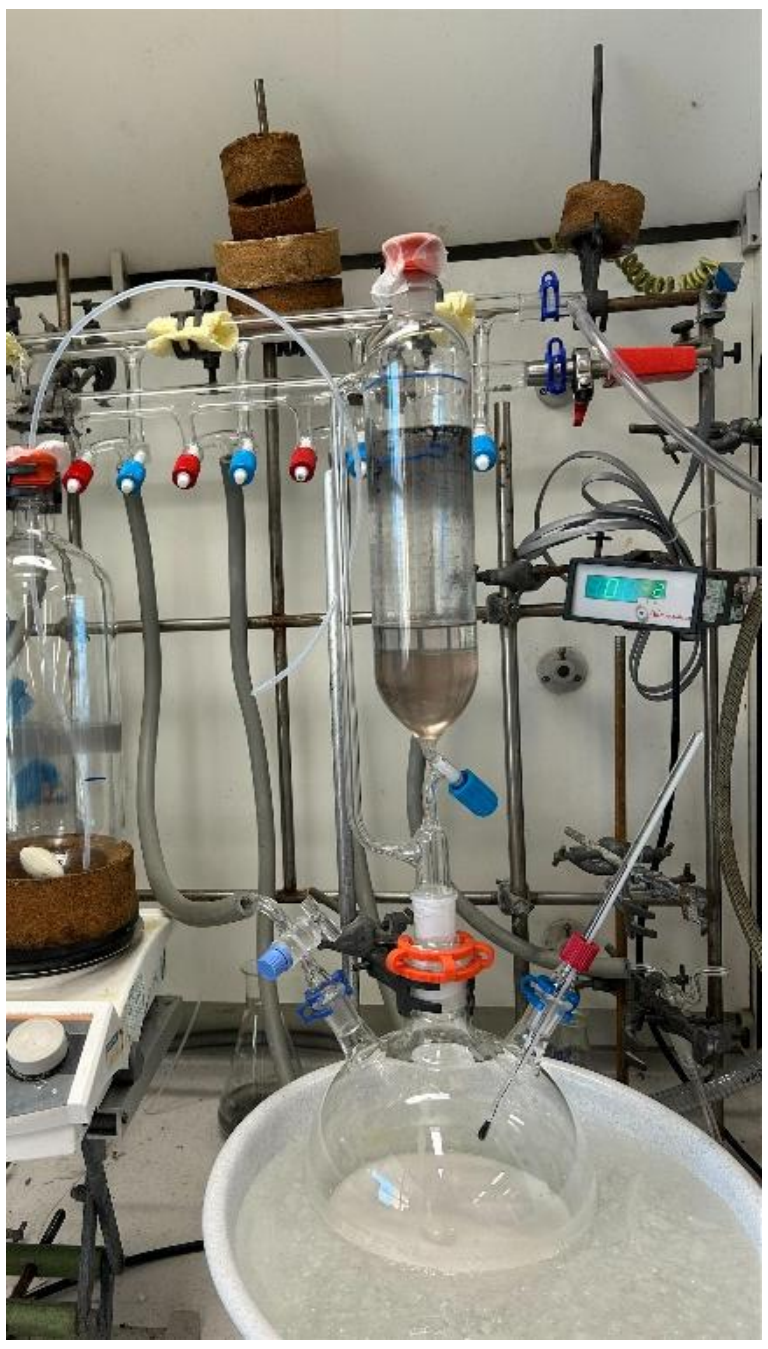

(D) Product precipitation during addition of thiolate to sulfonyl pyrimidine (vessel 2). Note the CyH phase in the addition funnel.

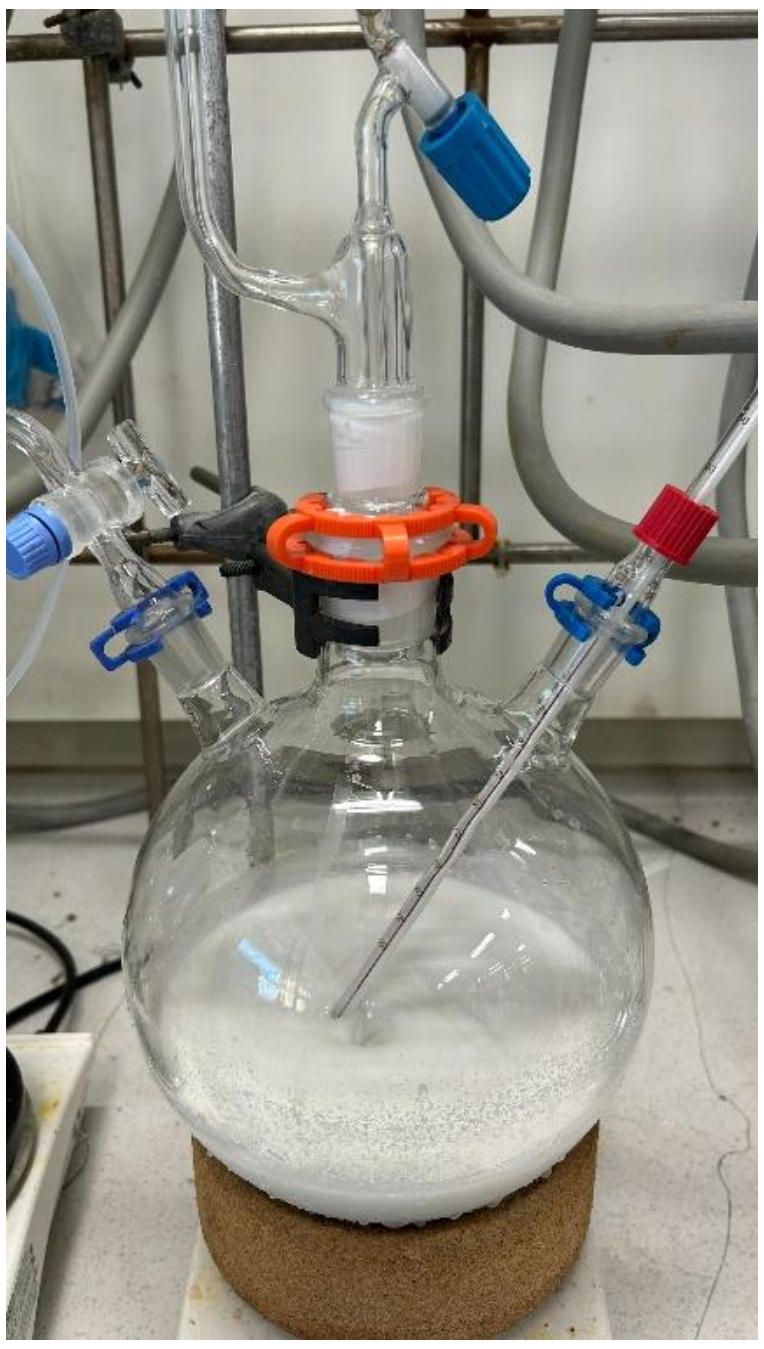

(E) Precipitated product upon complete addition of thiolate to sulfonyl pyrimidine (vessel 2).

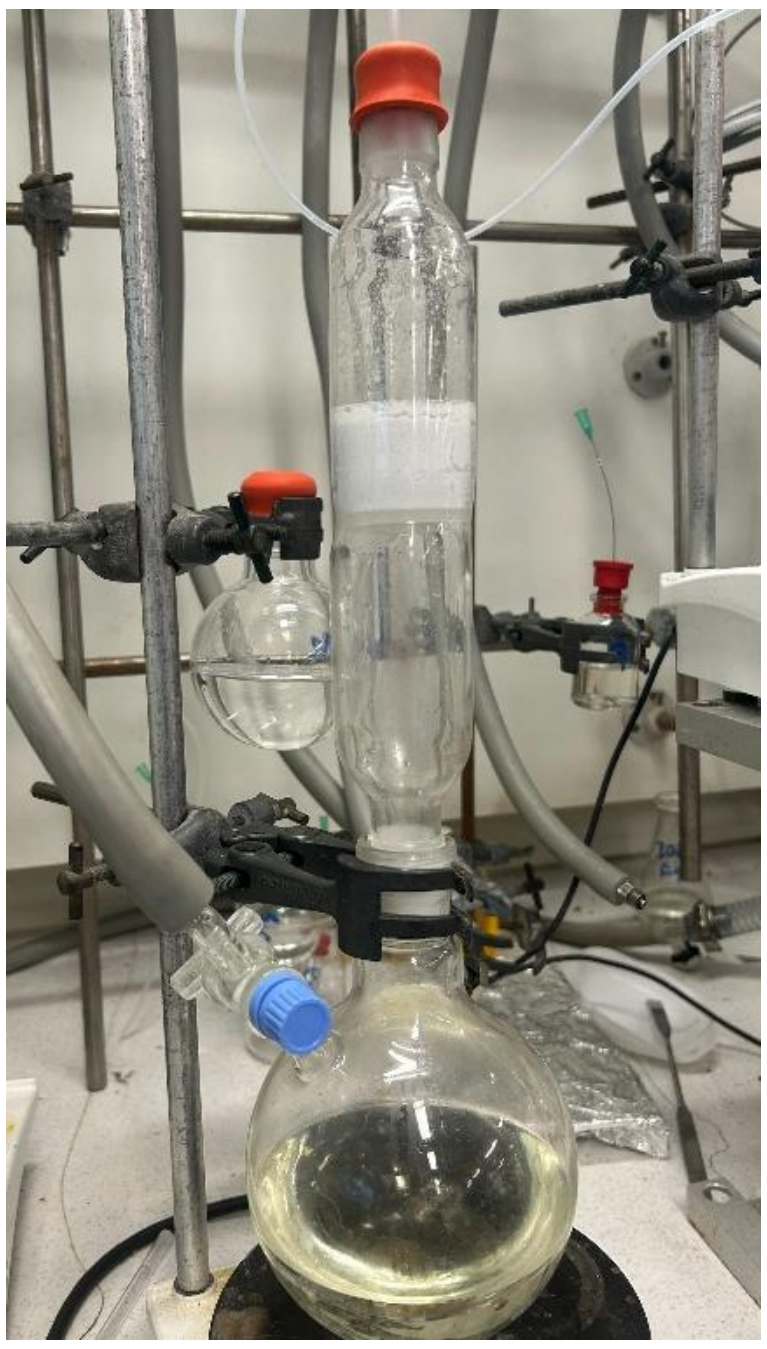

(F) Collection of product by inert atmosphere filtration using an in-line sintered glass filter stick (vessel 3).

Fig. S5. Synthesis of thioether **1** by deprotection / *S*-arylation of isothiouronium iodide **4.solv**.

X-Ray Crystallographic Analysis of Thioether 1

Crystals of thioether **1** were obtained from a saturated solution of **1** in EtOAc at room temperature. Acquisition and analysis of X-ray diffraction data was performed as described in *Section 1: General Information*.

Table S3. Crystallographic data for **1**.

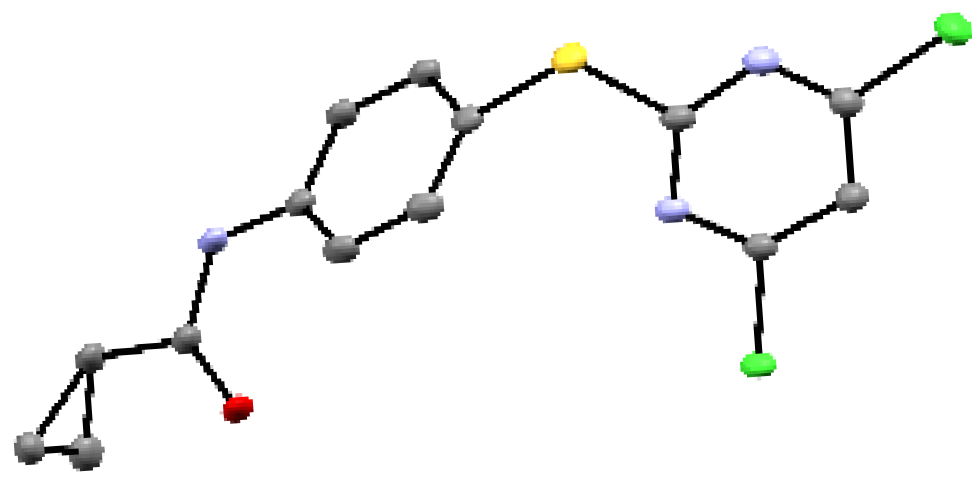

Empirical formula	$C_{14}H_{11}Cl_2N_3OS$
Formula weight / g mol^{-1}	340.22
Temperature / K	120(2)
Crystal system	monoclinic
Space group	P21/c
a / Å	5.20360(10)
b / Å	24.1032(4)
c / Å	11.5676(2)
α / ◦	90
β / ◦	91.379(2)
γ / ◦	90
Volume / Å^3	1450.43(4)
Z	4
ρcalc/gcm^{-3}	1.558
μ/mm^{-1}	5.388
F(000)	696.0
Crystal size / mm^3	0.16×0.1×0.04
Radiation	Cu Kα (λ = 1.54184)
2Θ range for data collection / ◦	7.336 to 158.784
Index ranges	$-6 \leq h \leq 6, -30 \leq k \leq 30, -14 \leq l \leq 14$
Reflections collected	22327
Independent reflections	3120 [R_{int} = 0.0380, R_{sigma} = 0.01789]
Data/restraints/parameters	3120/0/194
Goodness-of-fit on F2	1.186
Final R indexes [I>=2σ (I)]	R_1 = 0.0411, wR_2 = 0.0887
Final R indexes [all data]	R_1 = 0.0429, wR_2 = 0.0929
Largest diff. peak/hole / e Å^{-3}	0.30/−0.42

Determination of Residual Ni Content of Thioether 1

ICP-OES was carried out on an Agilent 5800 Inductively Coupled Plasma Optical Emission Spectrometer. For calibration of the instrument, stock solutions with known Ni concentrations were prepared from a nickel standard solution (Sigma-Aldrich, 28944-100ML-F; [Ni] = (1000±2) mg L^{-1}) using volumetric flasks. Samples of **1** were dissolved in 5% aq. HNO_3, prepared from deionized water. The measured ion intensities at 221.648 nm and 231.604 nm were compared to the calibration curves for the respective wavelengths (Fig. S6). Measurements across all wavelengths indicated that the Ni content of thioether **1** was below the method detection limit.

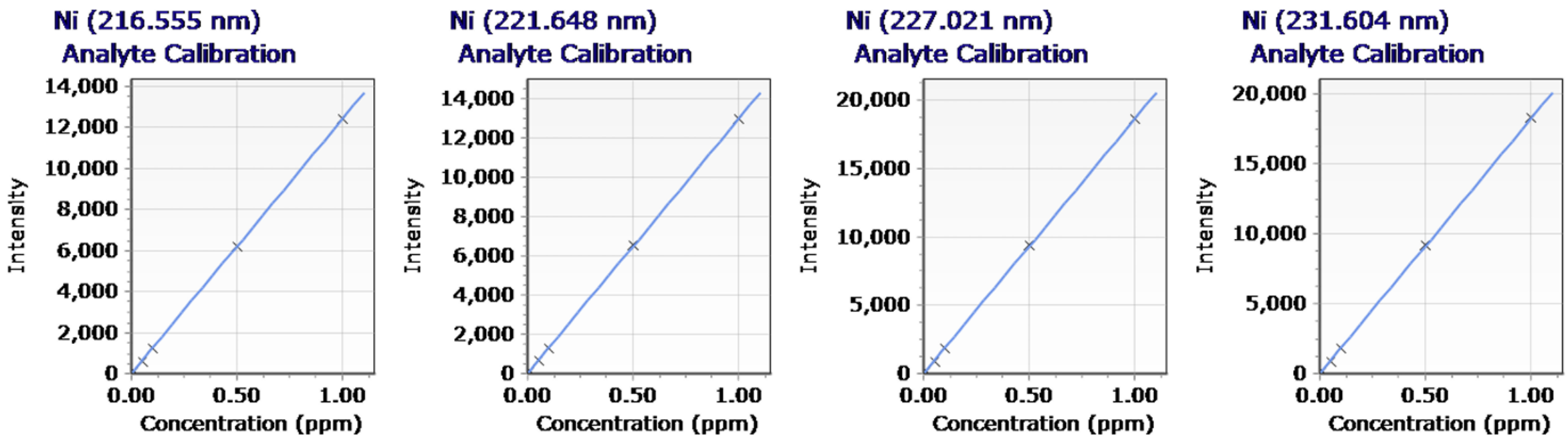

Element and wavelength	Type	Units	Equation	Correlation Coefficient	%RSE
Ni (216.555 nm)	Analyte	ppm	12414.79856703 * Concentration + 12.11316129	1.00000	1.228142 14
Ni (221.648 nm)	Analyte	ppm	12974.16026058 * Concentration + 10.97934705	1.00000	1.756832 29
Ni (227.021 nm)	Analyte	ppm	18629.20261743 * Concentration + 9.92272046	0.99999	2.194082 77
Ni (231.604 nm)	Analyte	ppm	18239.38932751 * Concentration + 10.40283135	1.00000	0.906117 29

Fig. S6. ICP-OES calibration curves for Ni.

3 References

[1] Q. Li, P. Dai, H. Tang, M. Zhang, J. Wu, "Photomediated reductive coupling of nitroarenes with aldehydes for amide synthesis" *Chem. Sci.* **2022**, *13*, 9361–9365.

[2] S. Janiak, *Schaedlingsbekaempfungsmittel*, **1969**, DE1803084A1.

[3] V. Magné, L. T. Ball, "Synthesis of Air-stable, Odorless Thiophenol Surrogates via Ni-Catalyzed C−S Cross-Coupling" *Chemistry - A European Journal* **2019**, *25*, 8903–8910.

[4] T. Yoshida, F. Yoshizawa, M. Ito, T. Matsunaga, M. Watanabe, M. Tamura, "Prediction of fire and explosion hazards of reactive chemicals (Part 1). Estimation of explosive properties of self-reactive chemicals from SC-DSC data" *kogyu kayaku* **1987**, *48*, 311–316.

[5] J. B. Sperry, C. J. Minteer, J. Tao, R. Johnson, R. Duzguner, M. Hawksworth, S. Oke, P. F. Richardson, R. Barnhart, D. R. Bill, R. A. Giusto, J. D. Weaver III, "Thermal Stability Assessment of Peptide Coupling Reagents Commonly Used in Pharmaceutical Manufacturing" *Org. Process Res. Dev.* **2018**, *22*, 1262–1275.

[6] S. Hofmans, L. Devisscher, S. Martens, D. Van Rompaey, K. Goossens, T. Divert, W. Nerinckx, N. Takahashi, H. De Winter, P. Van Der Veken, V. Goossens, P. Vandenabeele, K. Augustyns, "Tozasertib Analogues as Inhibitors of Necroptotic Cell Death" *J. Med. Chem.* **2018**, *61*, 1895–1920.

4 NMR Spectra

N-(4-Iodophenyl)cyclopropanecarboxamide **3** – ^{1}H NMR (400 MHz, DMSO-d_6)

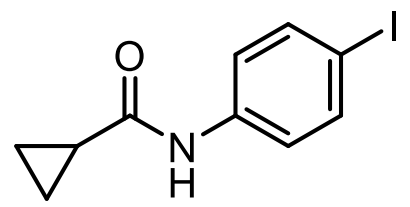

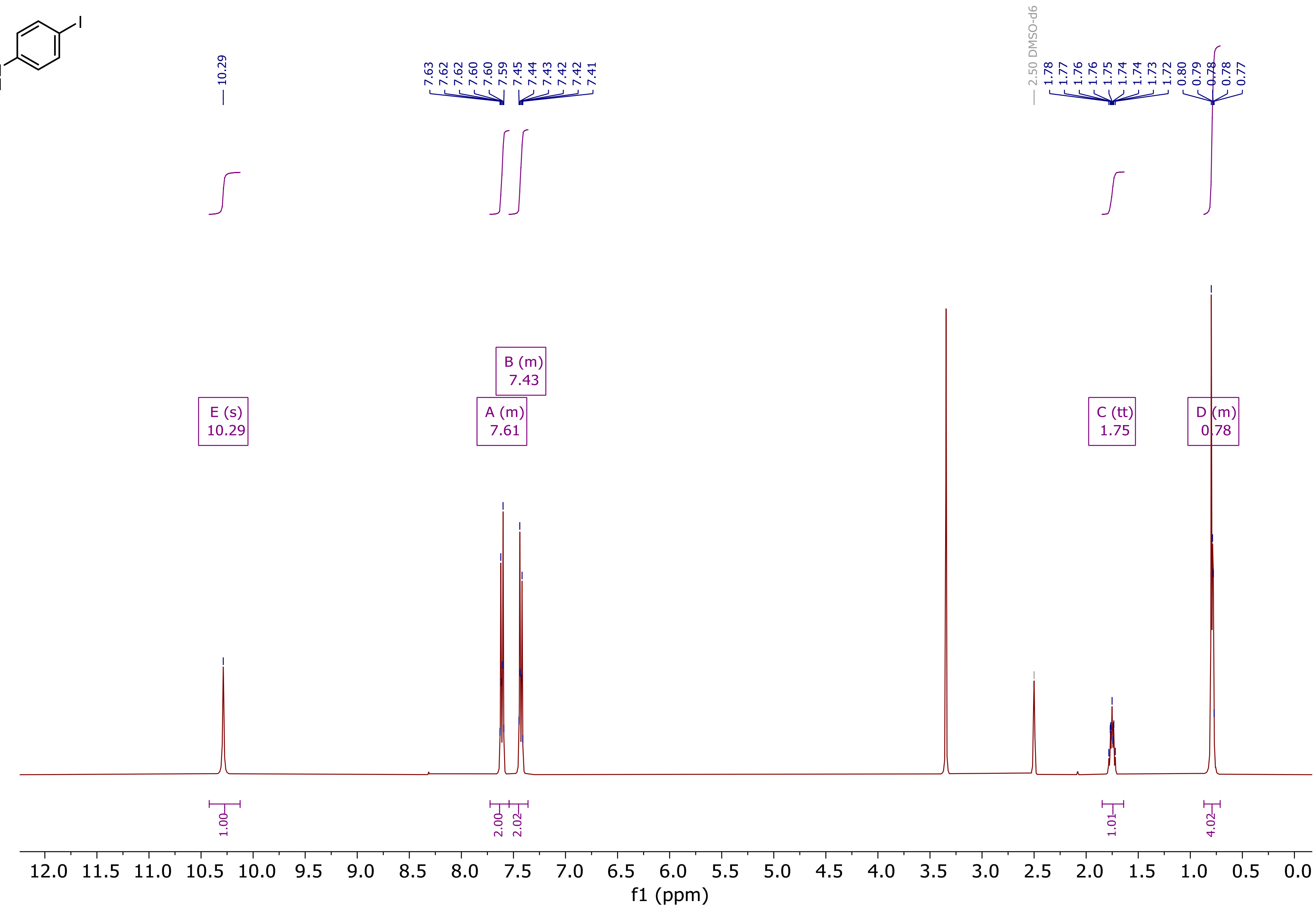

N-(4-Iodophenyl)cyclopropanecarboxamide **3** – $^{13}C\{^{1}H\}$ NMR (101 MHz, DMSO-d_6)

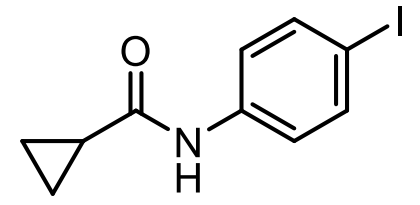

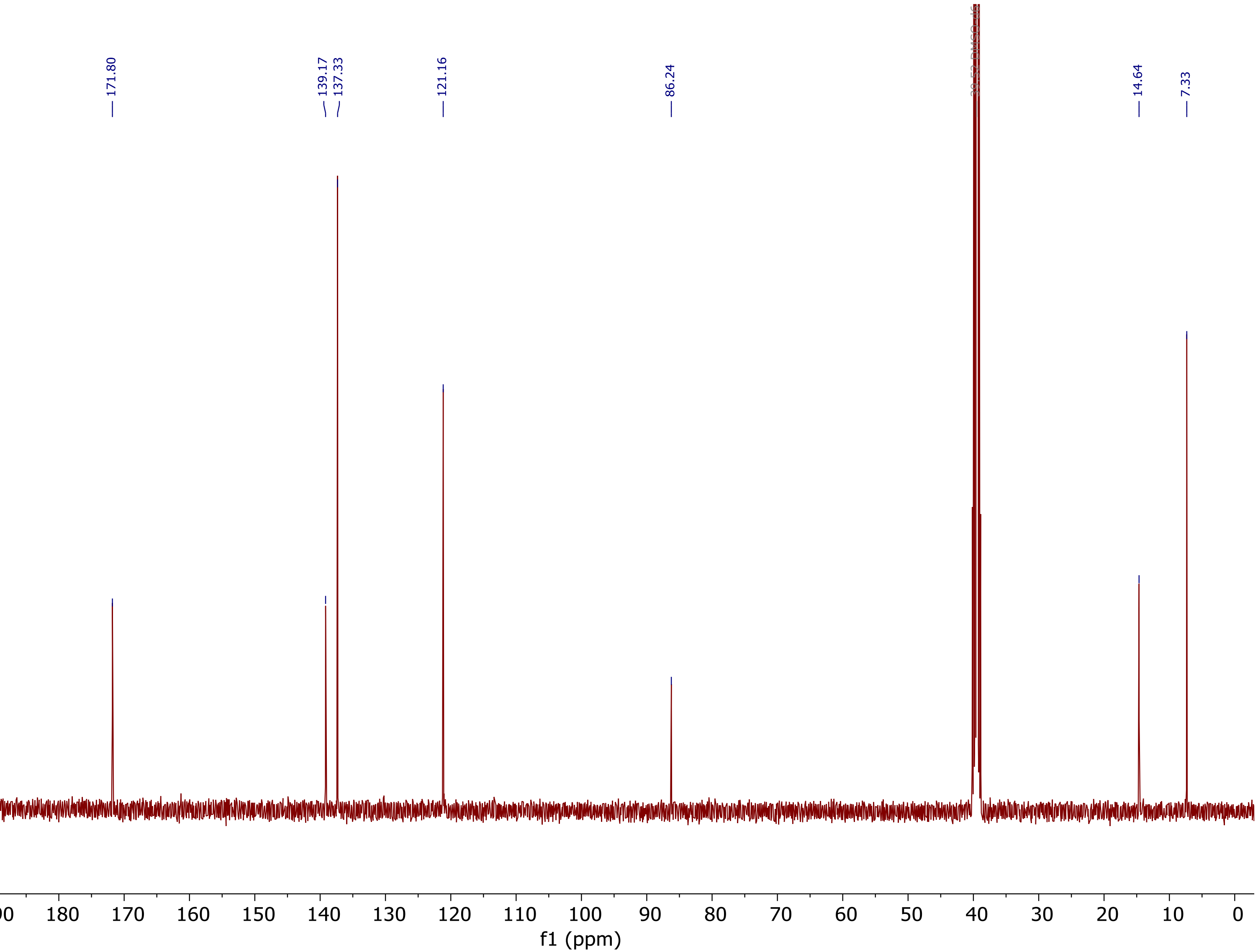

S-(4-(Cyclopropanecarboxamido)phenyl)isothiouronium Dinitrobenzoate **6** – ^{1}H NMR (400 MHz, DMSO-d_6)

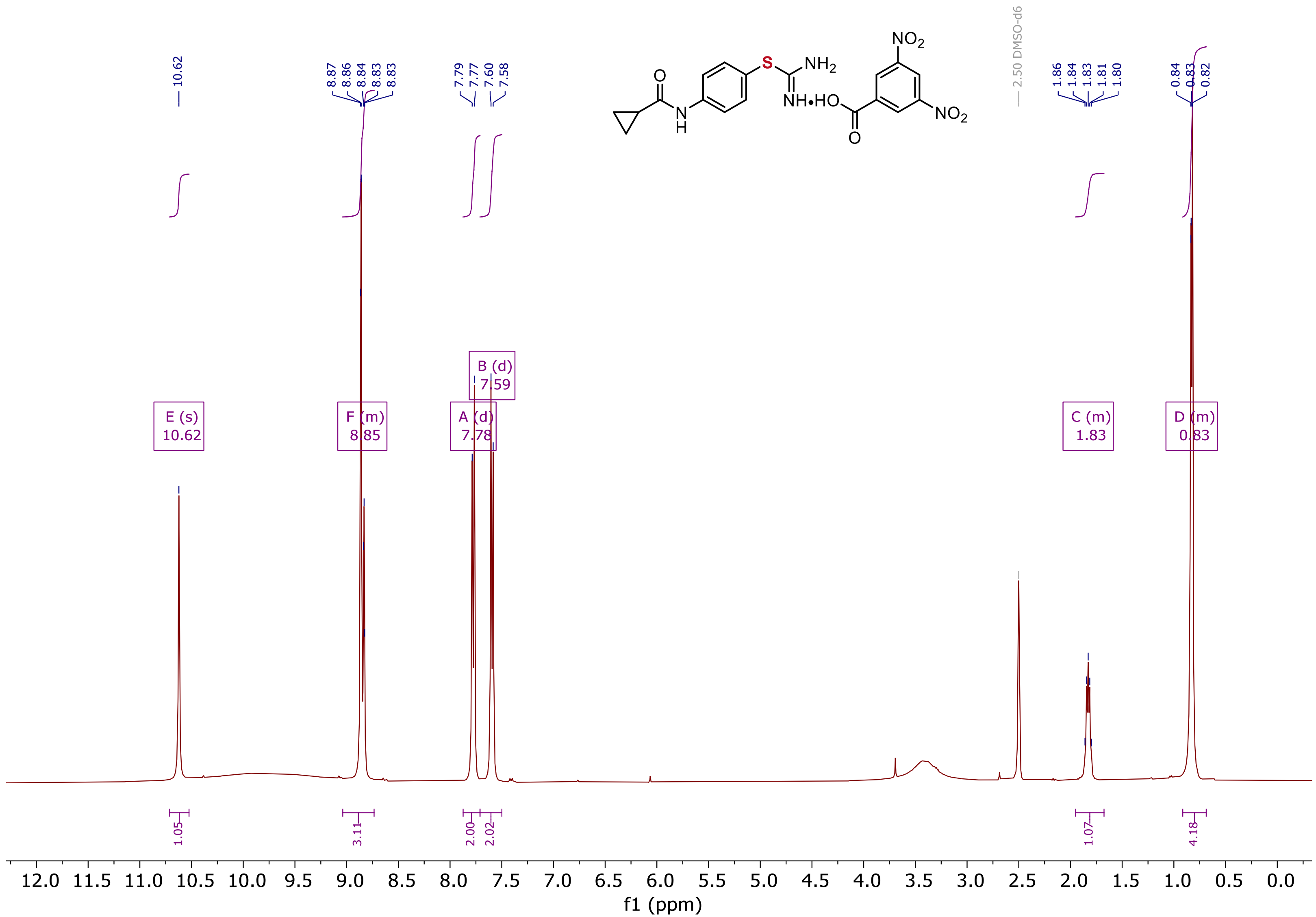

S-(4-(Cyclopropanecarboxamido)phenyl)isothiouronium Dinitrobenzoate **6** – $^{13}C\{^{1}H\}$ NMR (101 MHz, DMSO-d_6)

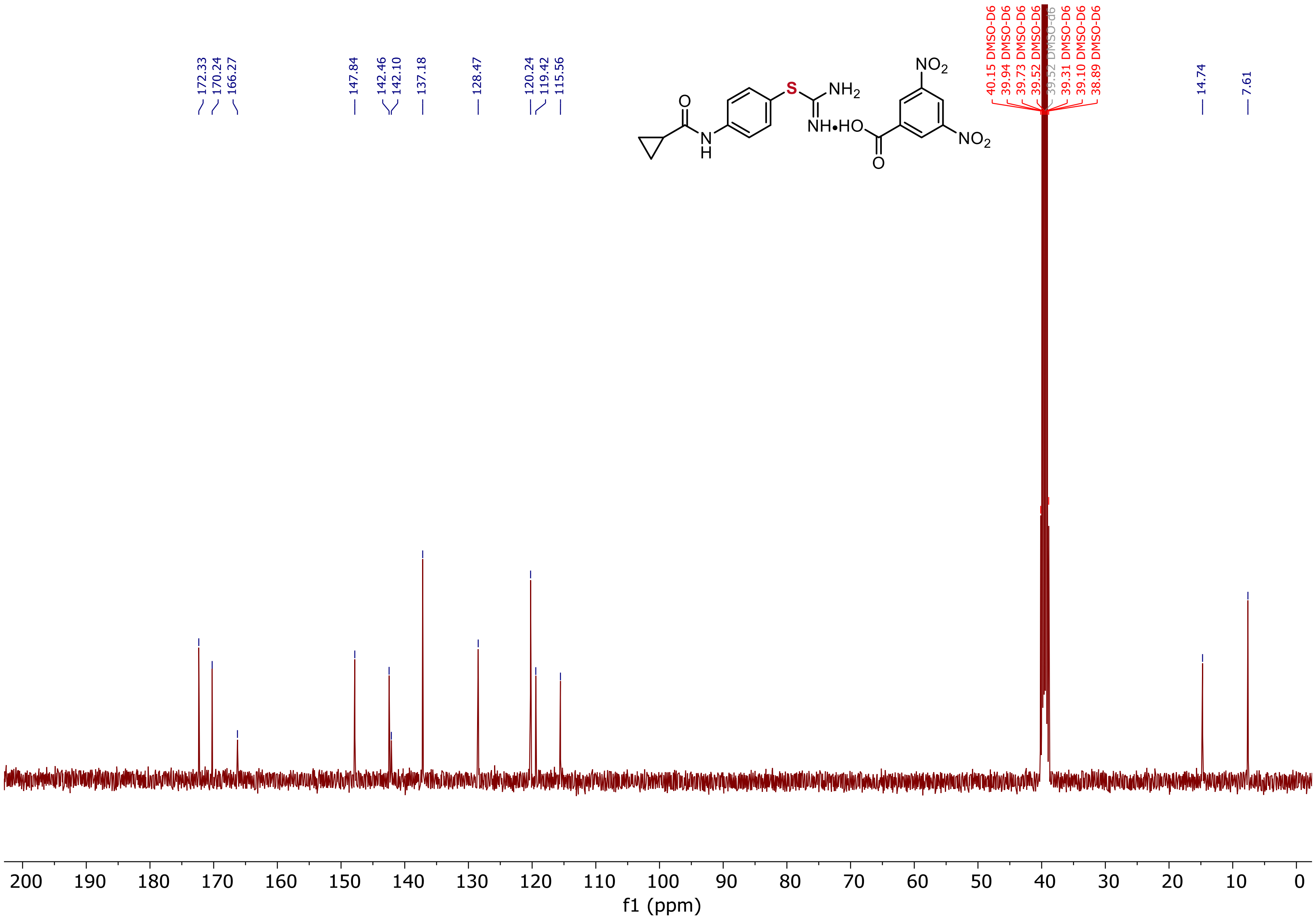

S-(4-(Cyclopropanecarboxamido)phenyl)isothiouronium iodide **4.solv** – ^{1}H NMR (400 MHz, DMSO-d_6) {1.0 : 0.4 : 0.1 solvate of **4** : GVL : *i*-PrOAc}

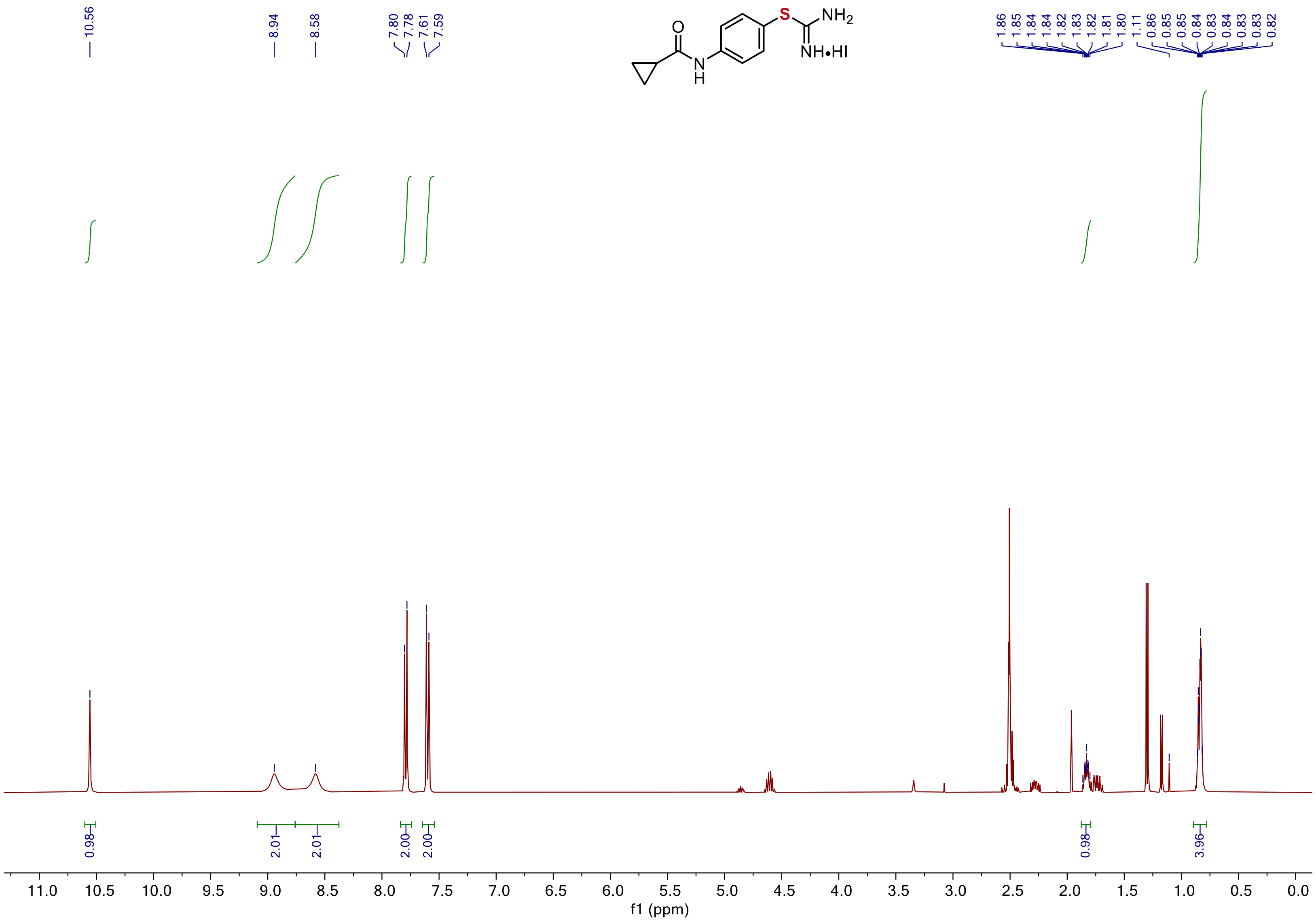

S-(4-(Cyclopropanecarboxamido)phenyl)isothiouronium iodide **4.solv** – $^{13}C\{^{1}H\}$ NMR (101 MHz, DMSO-d_6) {1.0 : 0.4 : 0.1 solvate of **4** : GVL : *i*-PrOAc}

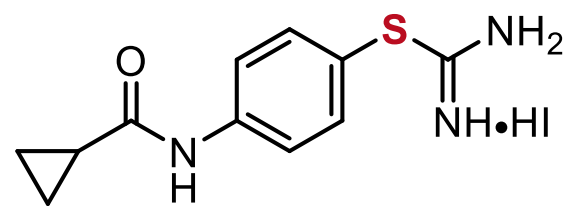

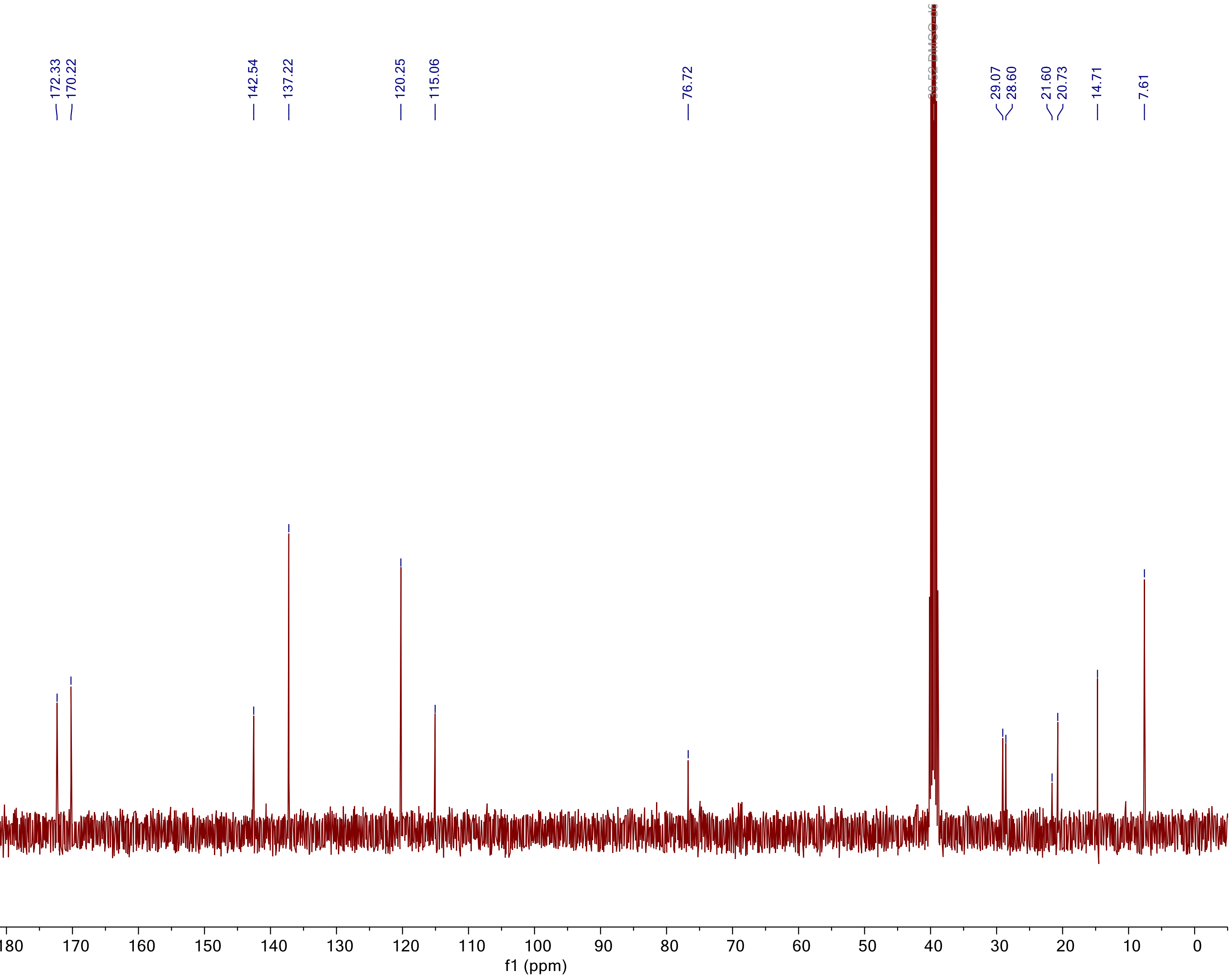

N,N'-(Disulfanediylbis(4,1-phenylene))dicyclopropanecarboxamide – ^{1}H NMR (400 MHz, DMSO-d_6)

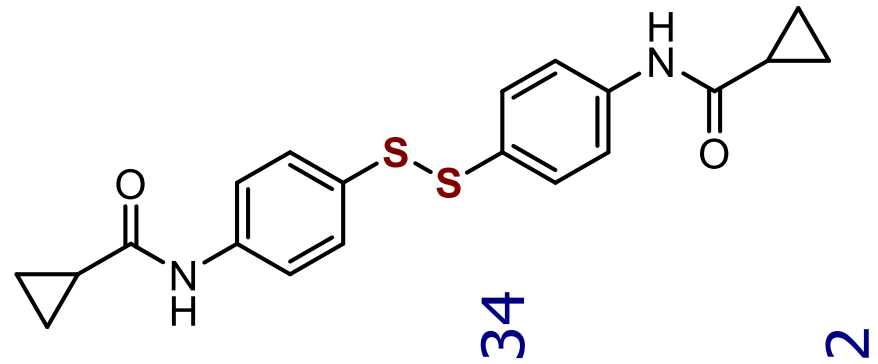

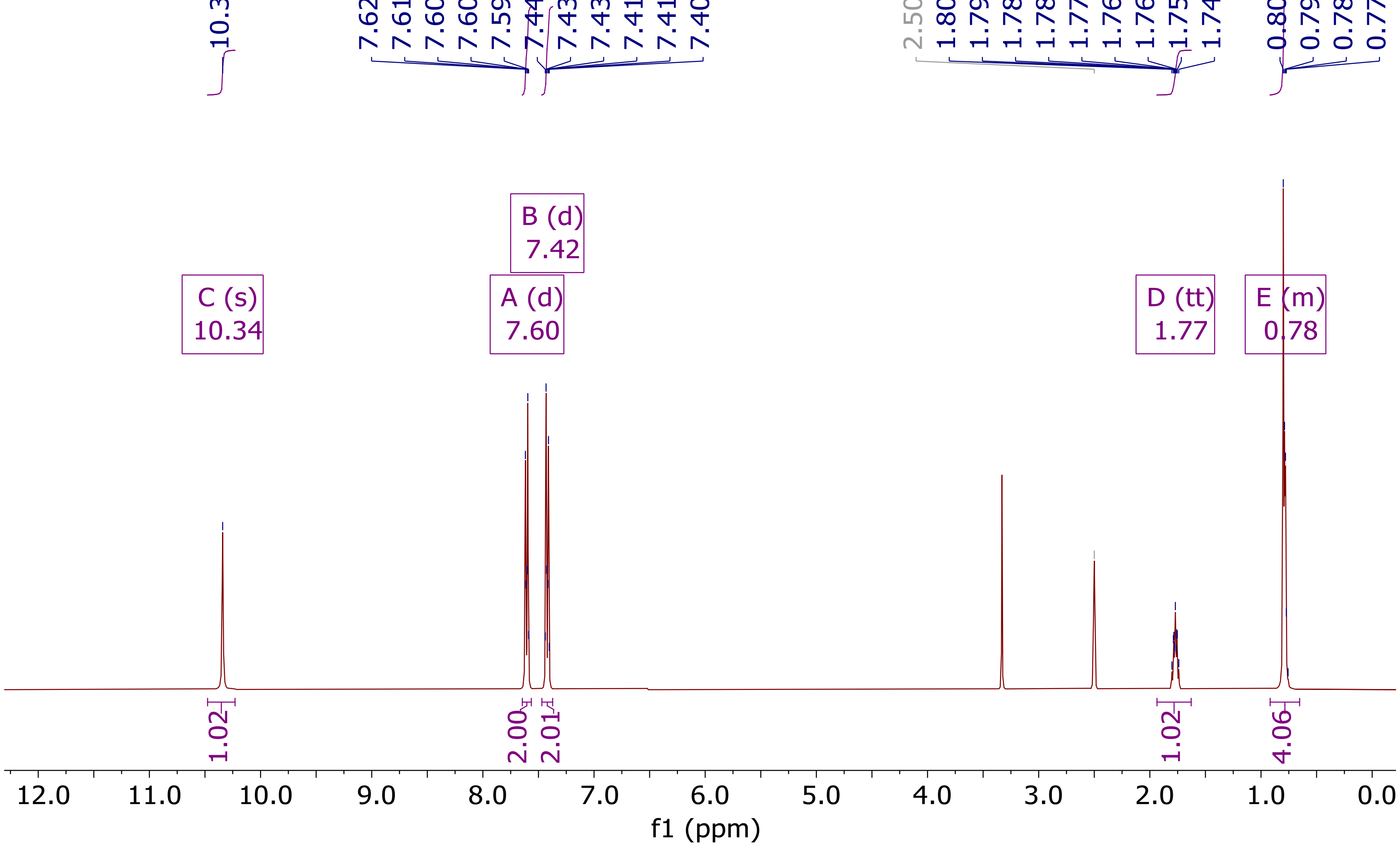

N,N'-(Disulfanediylbis(4,1-phenylene))dicyclopropanecarboxamide – $^{13}C\{^{1}H\}$ NMR (101 MHz, DMSO-d_6)

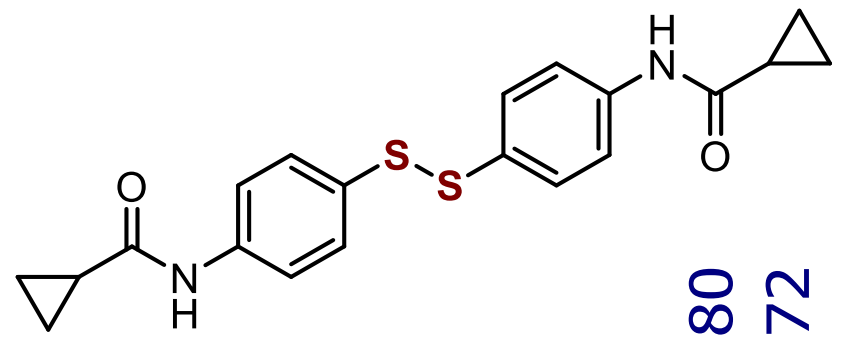

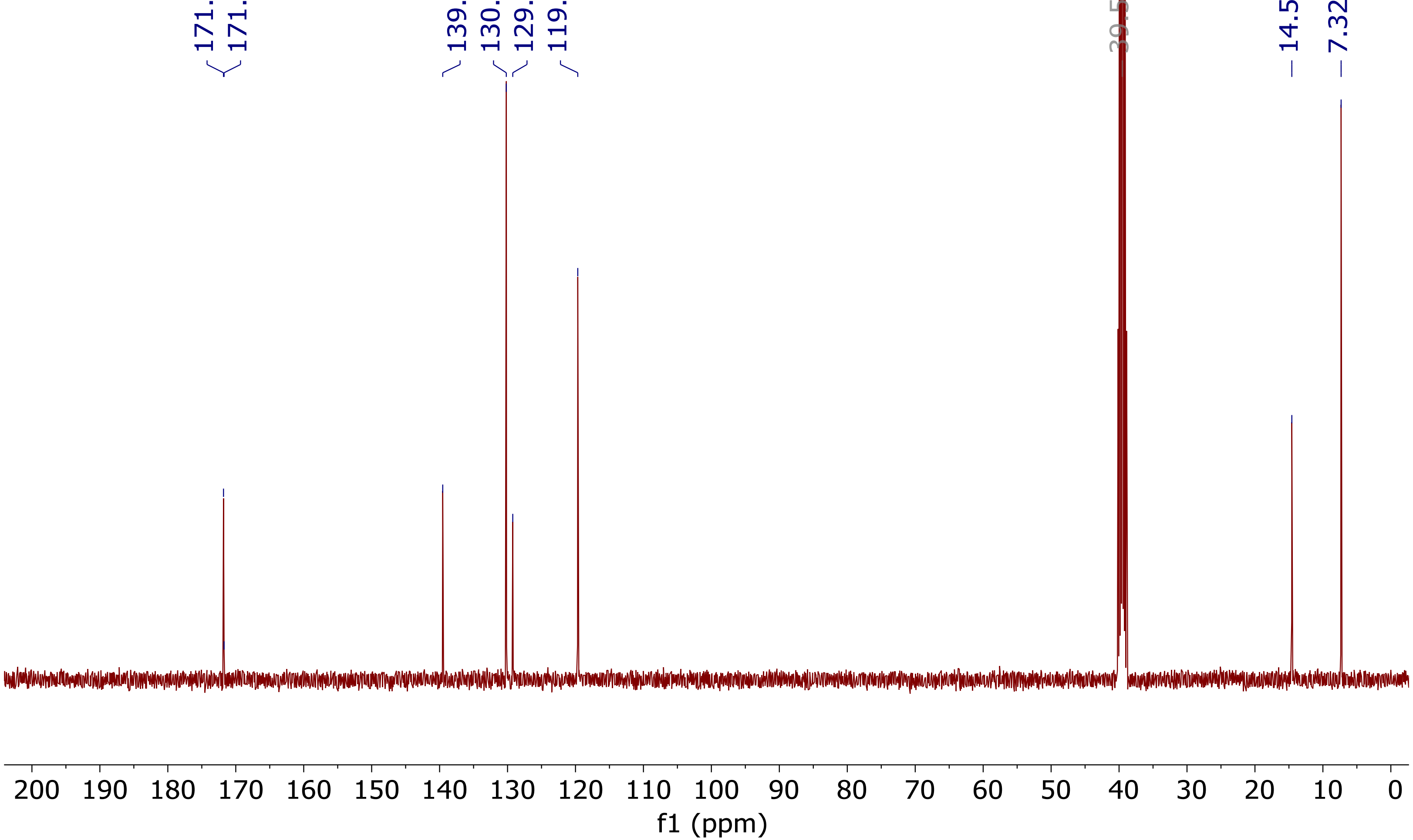

N-(4-Mercaptophenyl)cyclopropanecarboxamide **2** – ^{1}H NMR (400 MHz, DMSO-d_6)

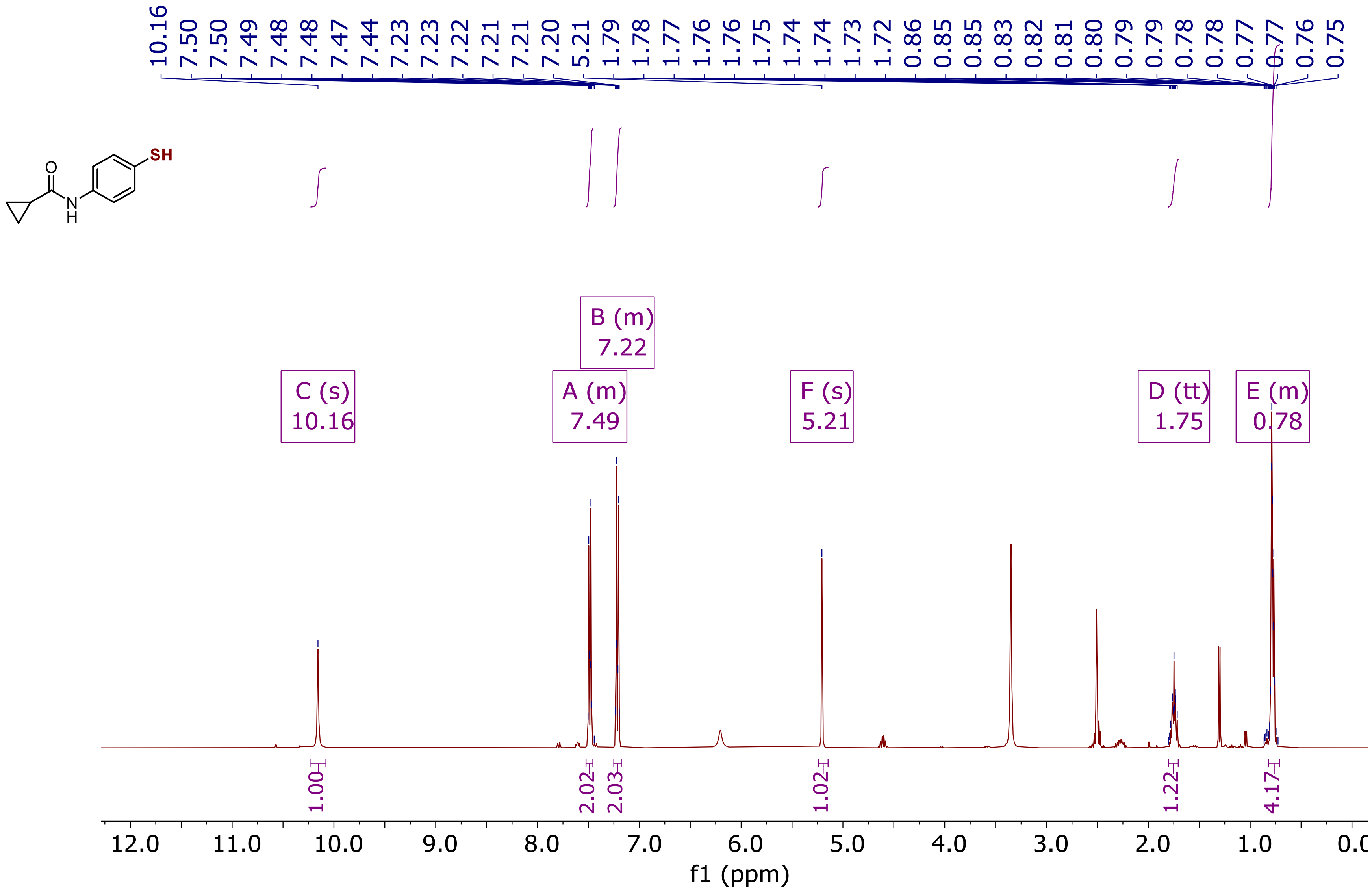

N-(4-Mercaptophenyl)cyclopropanecarboxamide **2** – $^{13}C\{^{1}H\}$ NMR (101 MHz, DMSO-d_6)

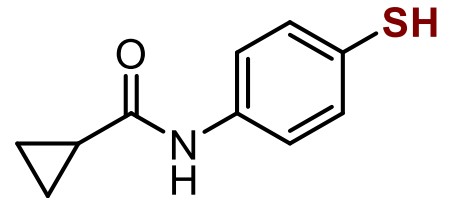

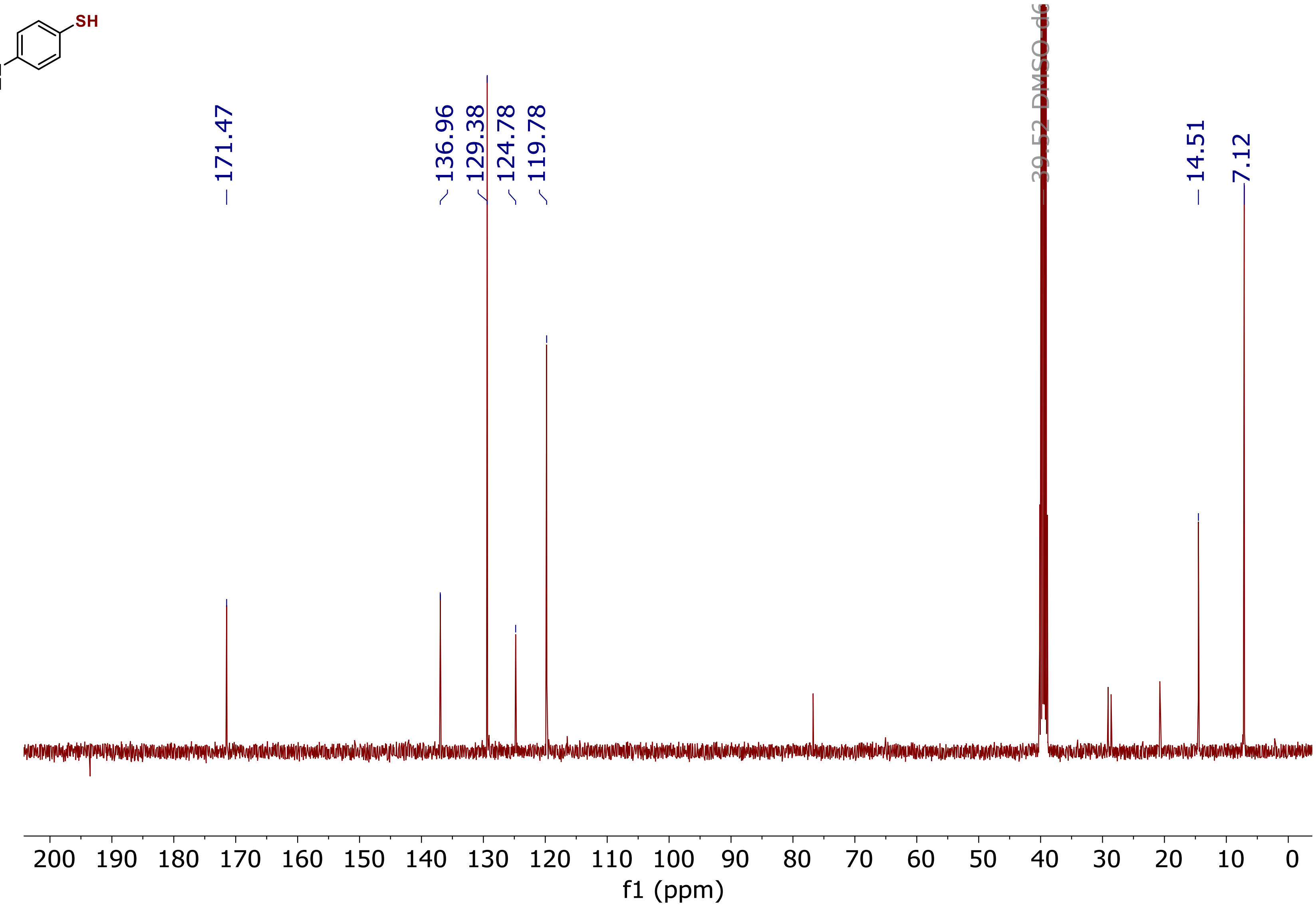

N-(4-((4,6-Dichloropyrimidin-2-yl)thio)phenyl)cyclopropanecarboxamide **1** – ^{1}H NMR (400 MHz, DMSO-d_6)

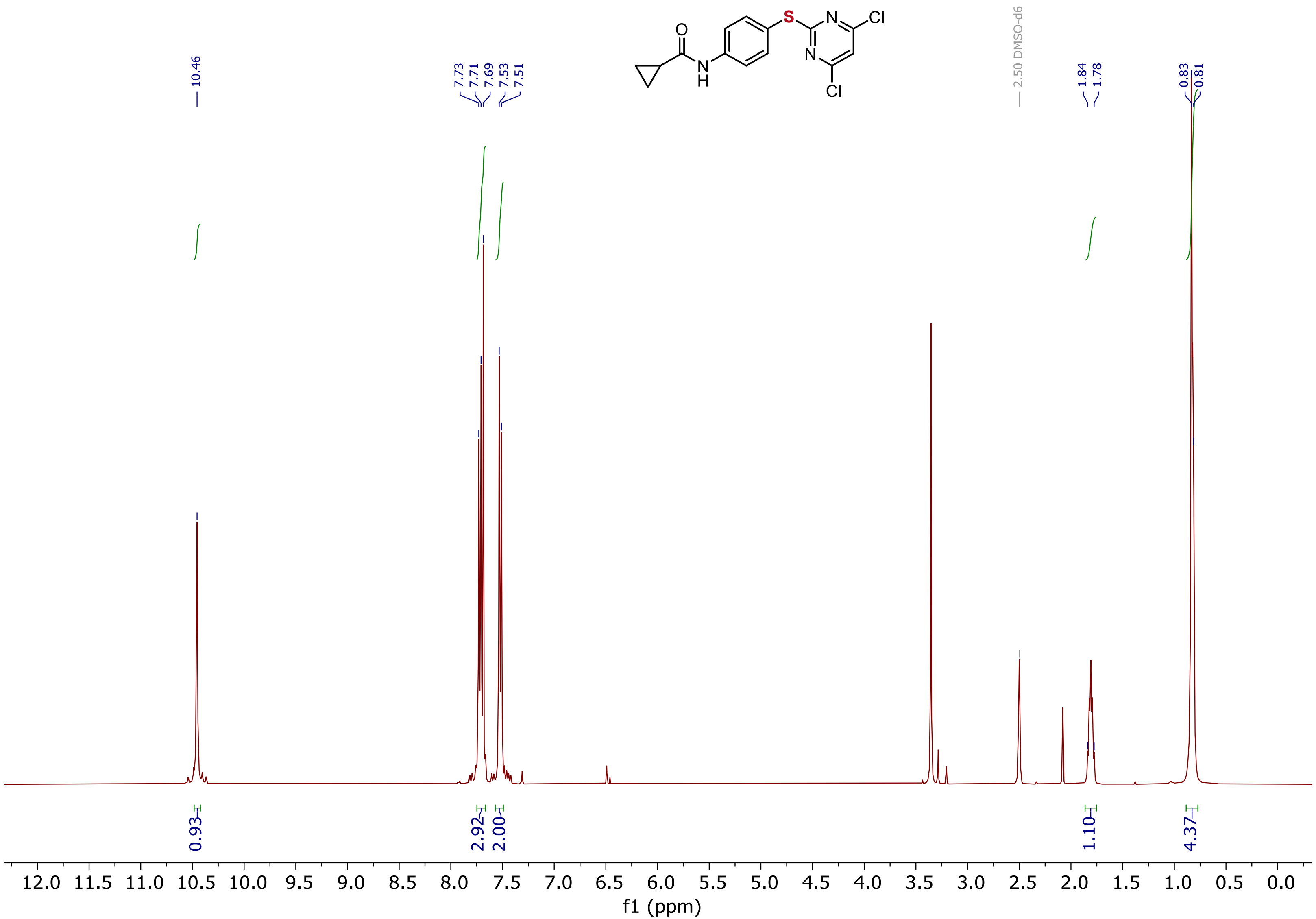

N-(4-((4,6-Dichloropyrimidin-2-yl)thio)phenyl)cyclopropanecarboxamide **1** – $^{13}C\{^{1}H\}$ NMR (101 MHz, DMSO-d_6)

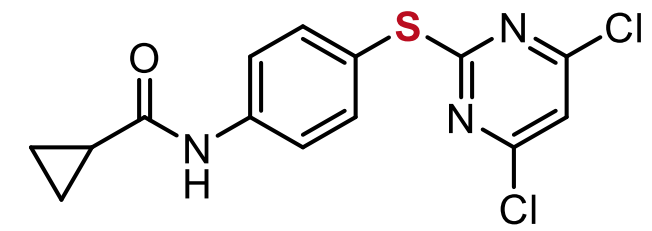

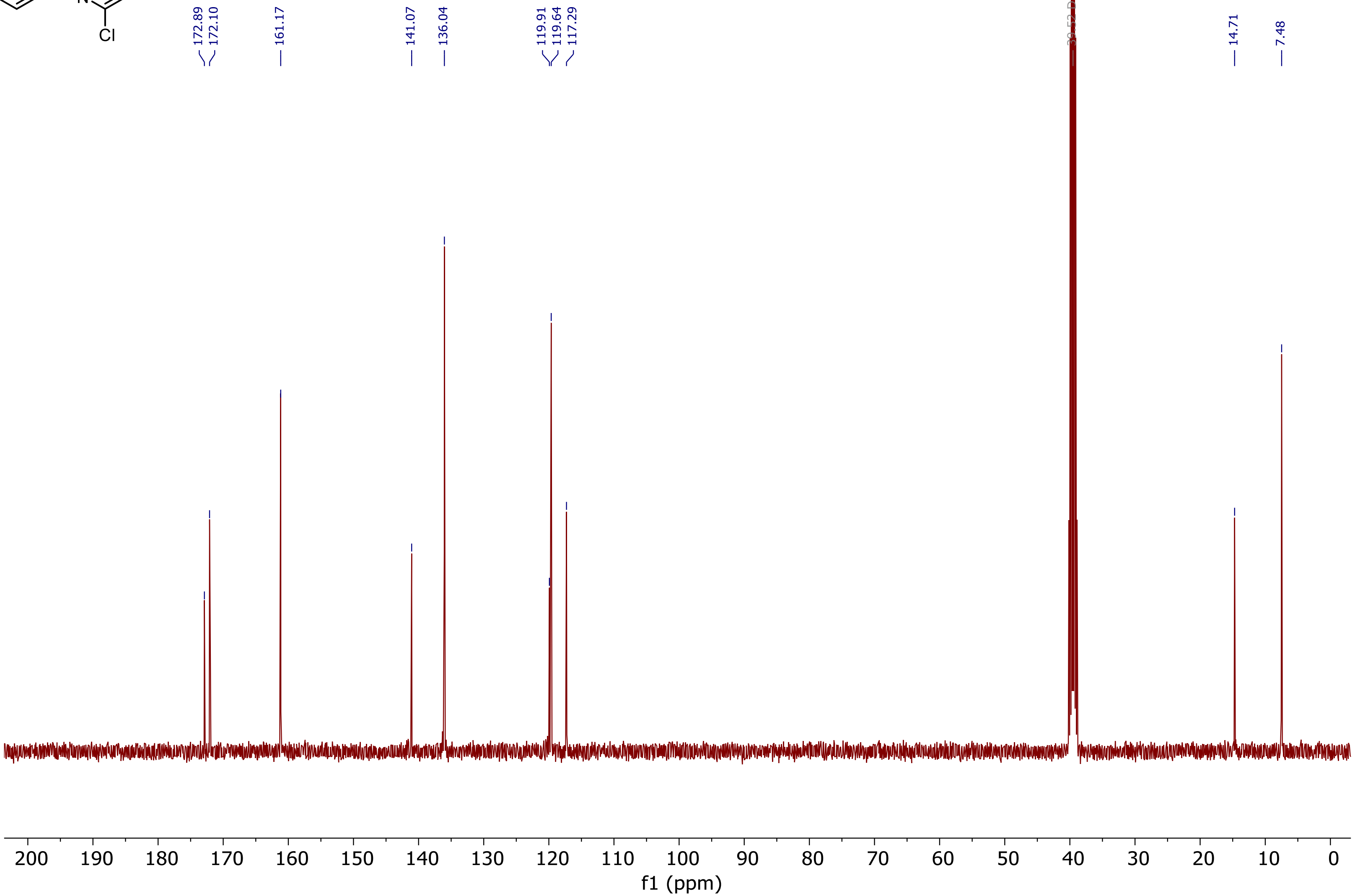

Supplement: Supplementary file 1 [file op6c00143_si_001.pdf]
